# Supplementary material for: Phenotypic covariance across the entire spectrum of relatedness for 86 billion pairs of individuals
Source: Nat Commun. 2021 Feb 16;12:1050. doi: 10.1038/s41467-021-21283-4 (PMC7886899; doi:10.1038/s41467-021-21283-4)
Supplement: Supplementary file 1 — Supplementary File [file 41467_2021_21283_MOESM1_ESM.pdf]

Phenotypic covariance across the entire spectrum of relatedness for 86 billion pairs of individuals

Kemper et al.

Supplementary File

This file contains:

Supplementary Figures 2
Supplementary Tables 46
Supplementary Notes 51
Supplementary References 71

Supplementary Figures

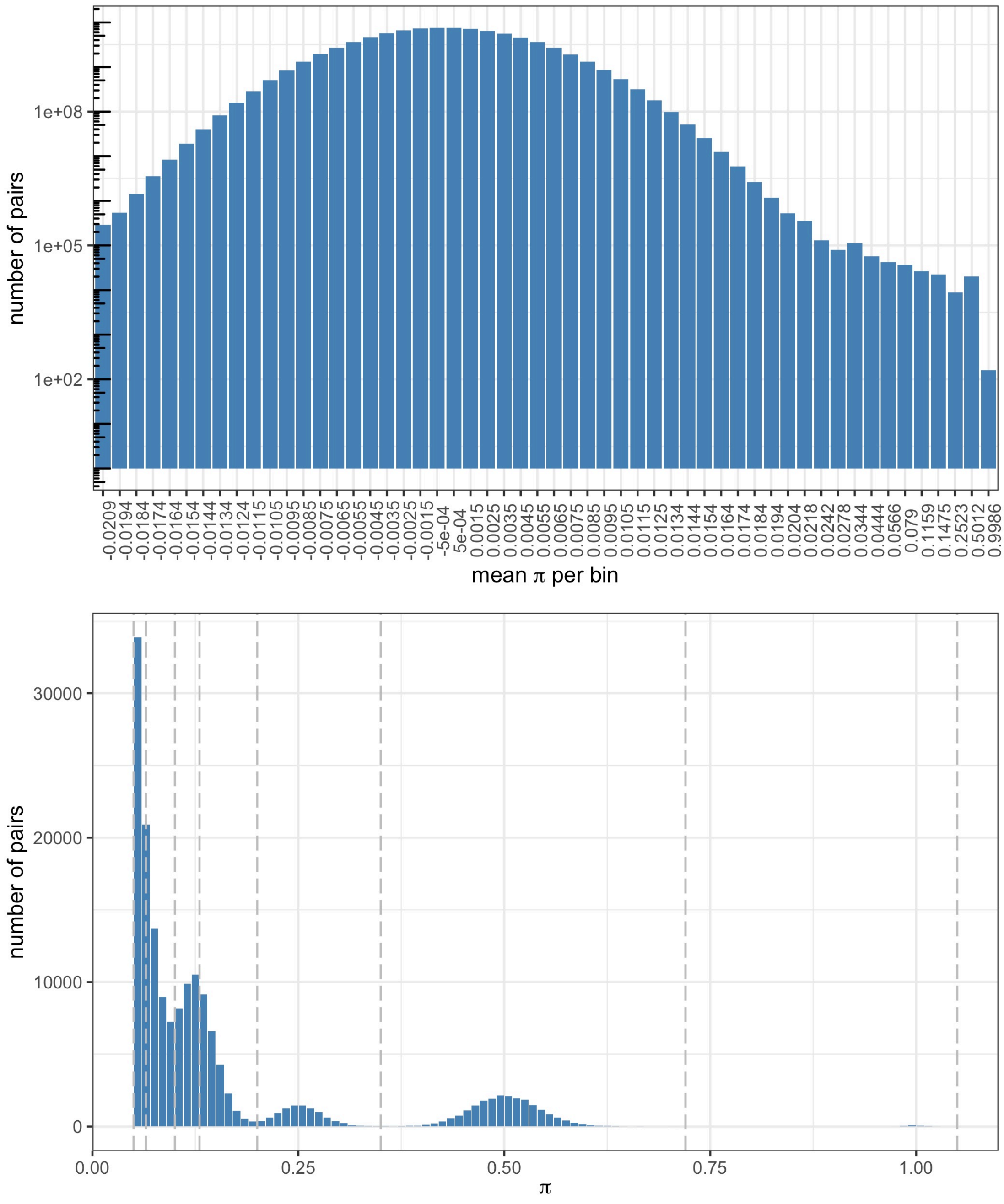

Supplementary Figure 1. Number of pairs per bin from the genomic relationship matrix in the UK Biobank. Shown are the 54 relationship bins (top, log scale) and just close relatives (bottom, observed scale), with bin boundaries marked by vertical lines (bottom).

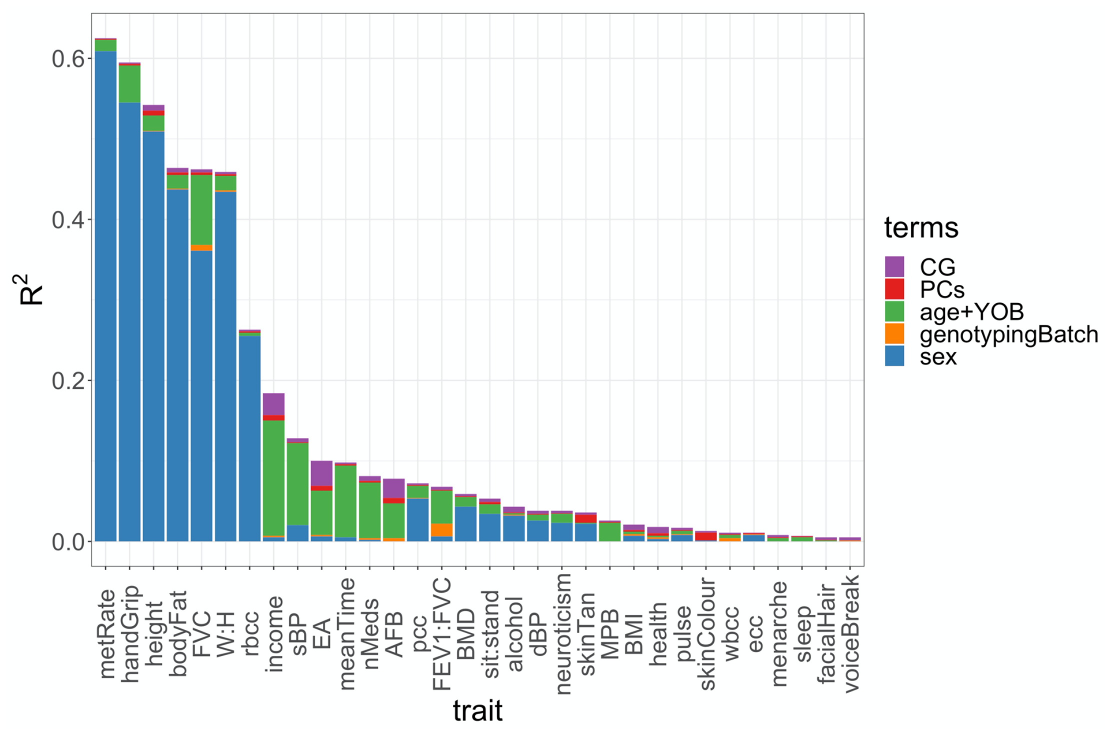

Supplementary Figure 2. Variance explained (R^2) by fixed effects for 32 quantitative and ordered categorical traits in the UK Biobank. Fixed effects were fitted in the order of sex (2 levels), genotyping batch (106 levels), age at assessment and year of birth (age+YOB, 65 levels), genetic stratification (25 principal components, PCs) and geographic stratification (birth contemporary group, CG; 378 levels).

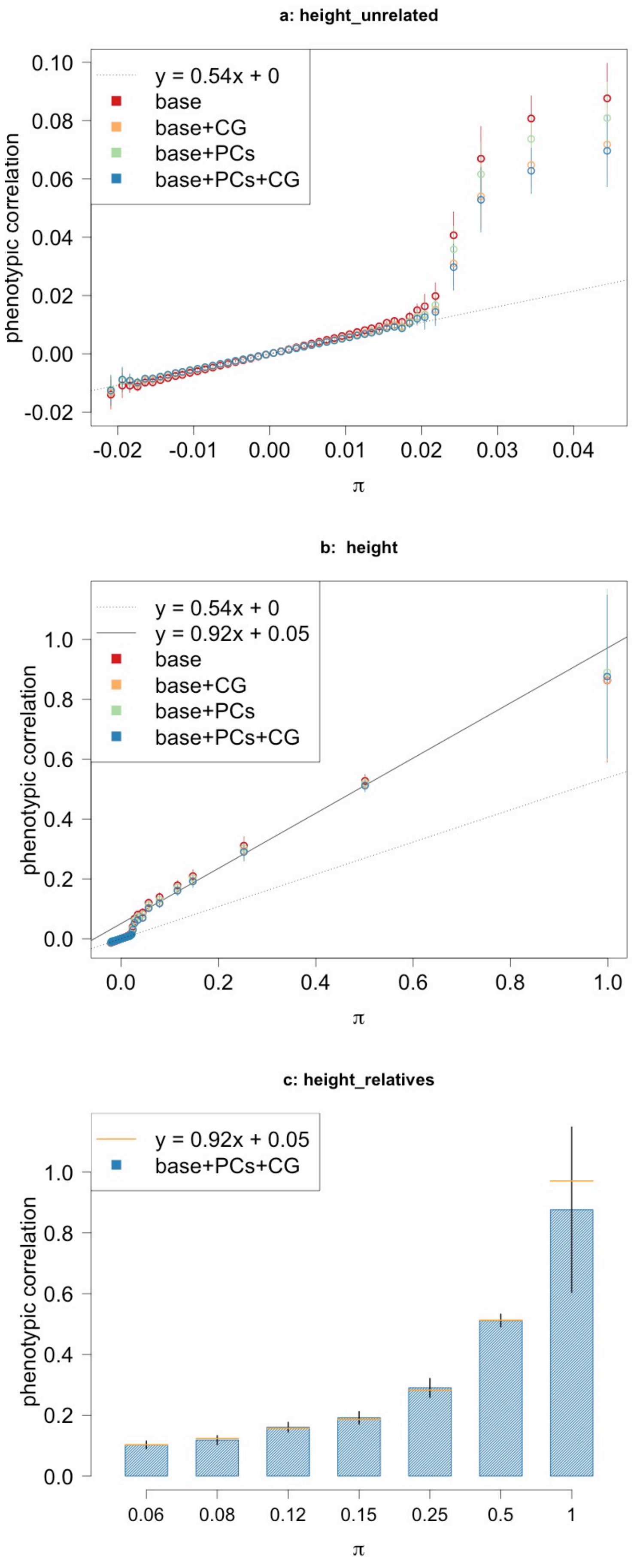
a: height_unrelated
y = 0.54x + 0
base
base+CG
base+PCs
base+PCs+CG
phenotypic correlation
π
b: height
y = 0.54x + 0
y = 0.92x + 0.05
base
base+CG
base+PCs
base+PCs+CG
phenotypic correlation
π
c: height_relatives
y = 0.92x + 0.05
base+PCs+CG
phenotypic correlation
0.06
0.08
0.12
0.15
0.25
0.5
1
π

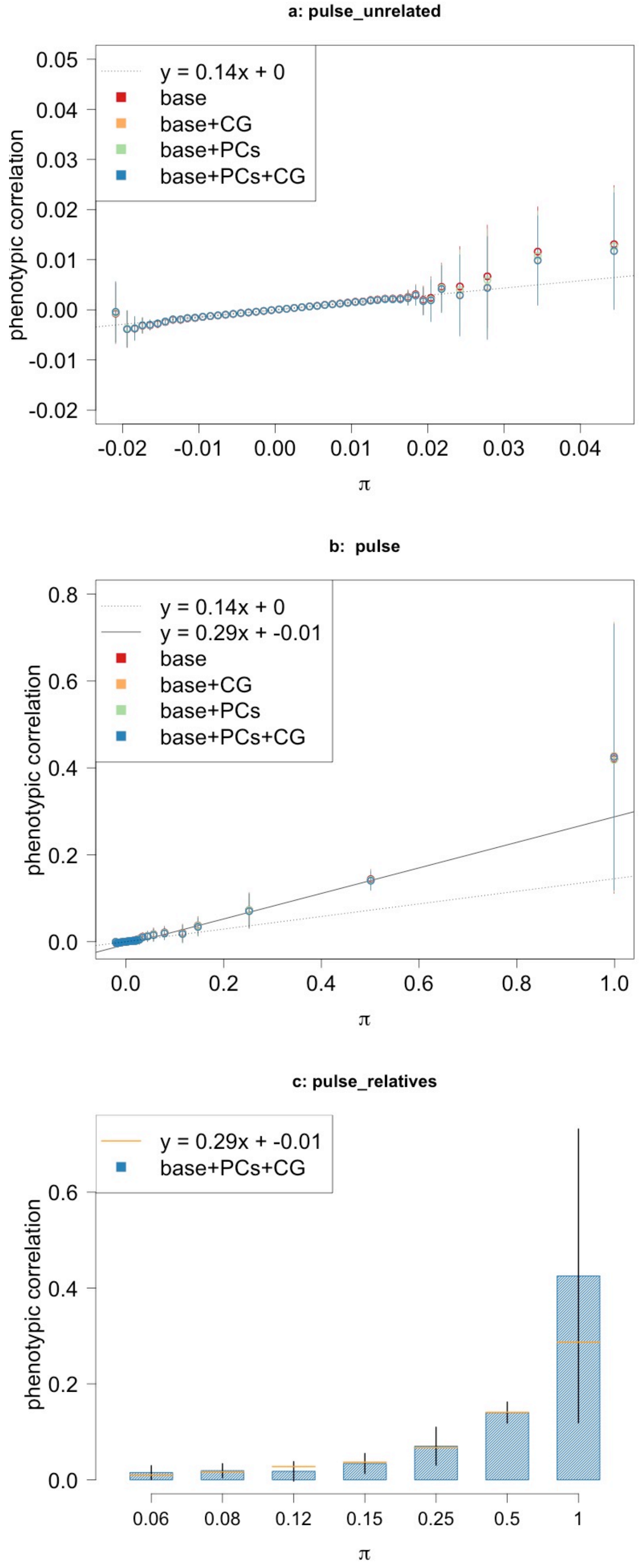
a: pulse_unrelated
y = 0.14x + 0
base
base+CG
base+PCs
base+PCs+CG
phenotypic correlation
0.05
0.04
0.03
0.02
0.01
0.00
-0.01
-0.02
-0.02
-0.01
0.00
0.01
0.02
0.03
0.04
π
b: pulse
y = 0.14x + 0
y = 0.29x + -0.01
base
base+CG
base+PCs
base+PCs+CG
phenotypic correlation
0.8
0.6
0.4
0.2
0.0
0.0
0.2
0.4
0.6
0.8
1.0
π
c: pulse_relatives
y = 0.29x + -0.01
base+PCs+CG
phenotypic correlation
0.6
0.4
0.2
0.0
0.06
0.08
0.12
0.15
0.25
0.5
1
π

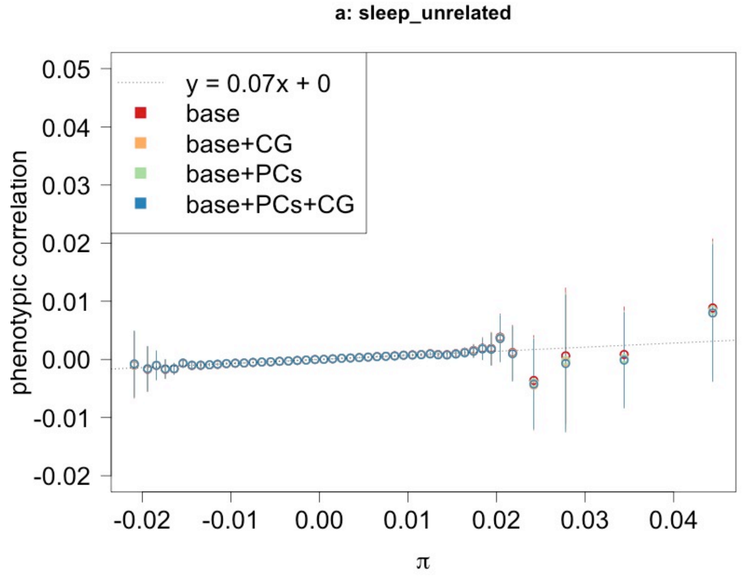
a: sleep_unrelated
y = 0.07x + 0
base
base+CG
base+PCs
base+PCs+CG
phenotypic correlation
π

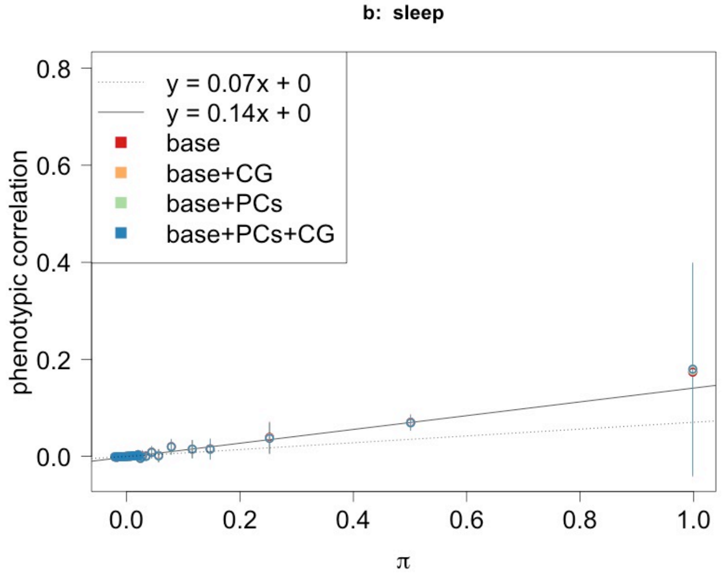
b: sleep
y = 0.07x + 0
y = 0.14x + 0
base
base+CG
base+PCs
base+PCs+CG
phenotypic correlation
π

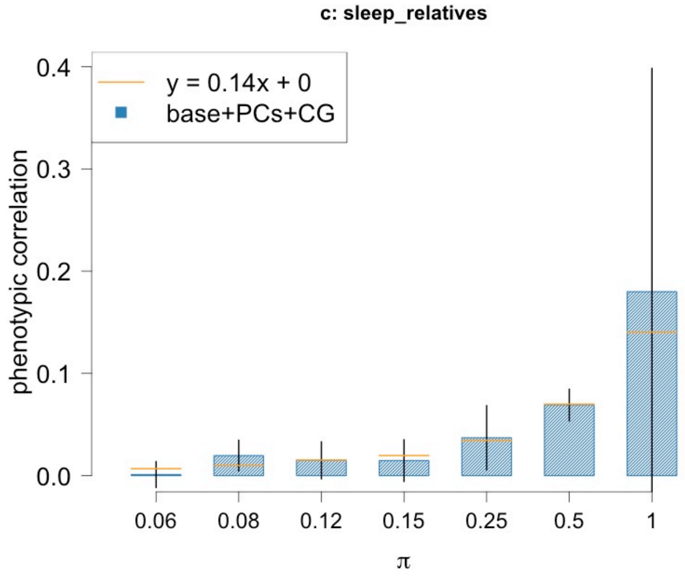
c: sleep_relatives
y = 0.14x + 0
base+PCs+CG
phenotypic correlation
0.06
0.08
0.12
0.15
0.25
0.5
1
π

a: dBP_unrelated

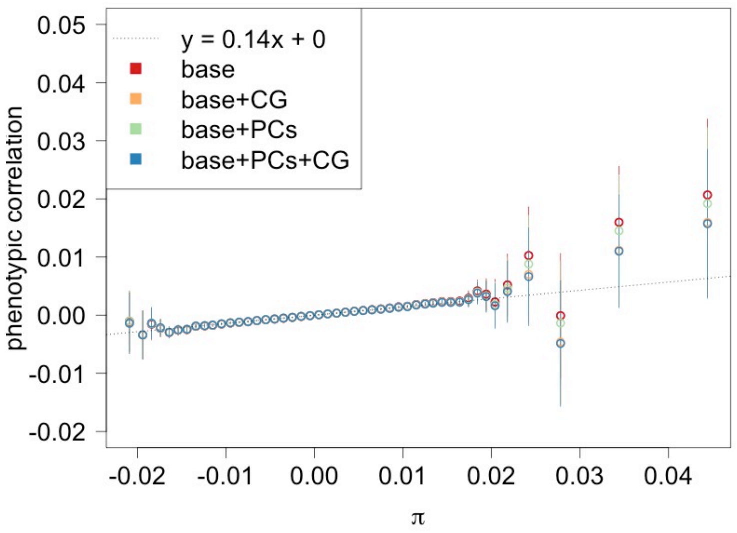

b: dBP

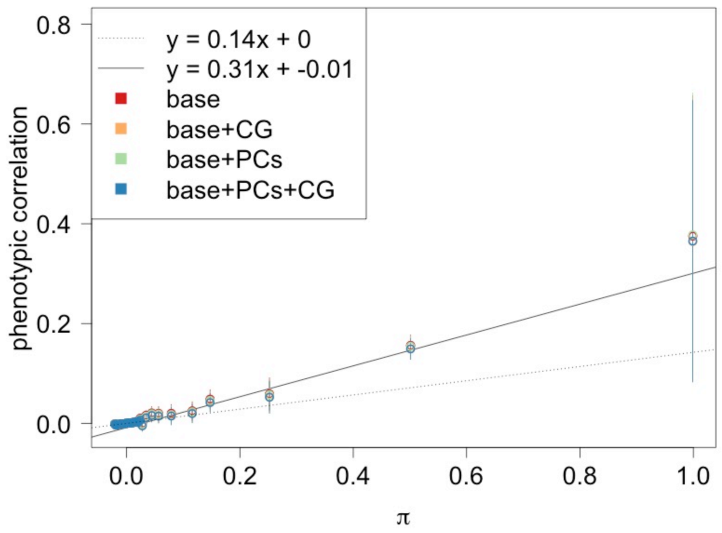

c: dBP_relatives

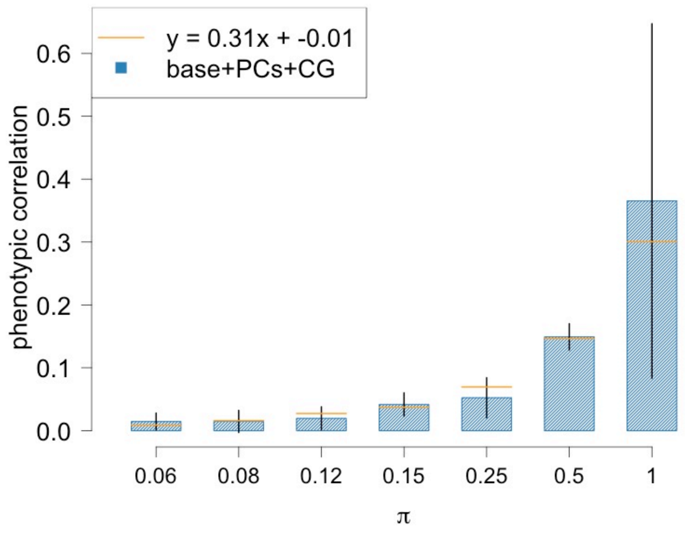

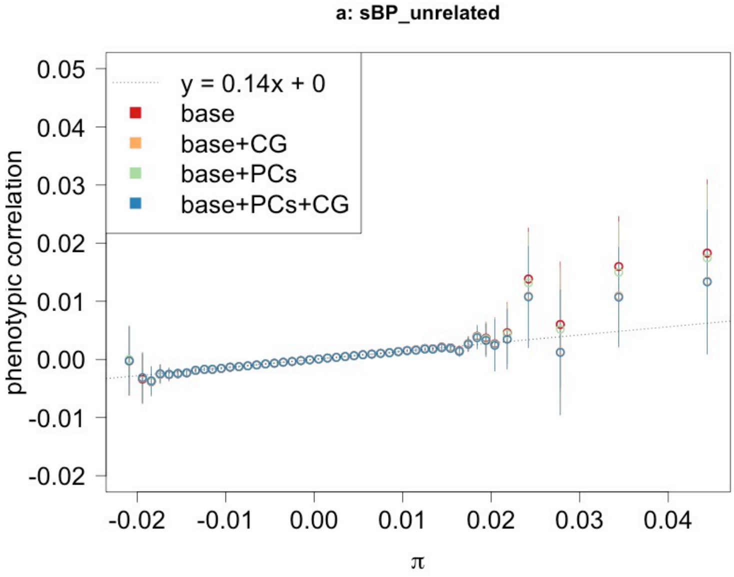
a: sBP_unrelated
y = 0.14x + 0
base
base+CG
base+PCs
base+PCs+CG
phenotypic correlation
0.05
0.04
0.03
0.02
0.01
0.00
-0.01
-0.02
-0.02
-0.01
0.00
0.01
0.02
0.03
0.04
π

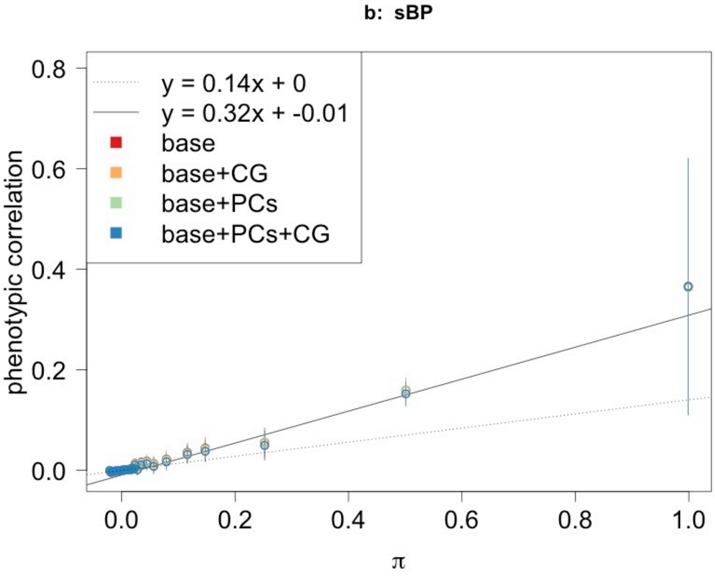
b: sBP
y = 0.14x + 0
y = 0.32x + -0.01
base
base+CG
base+PCs
base+PCs+CG
phenotypic correlation
0.8
0.6
0.4
0.2
0.0
0.0
0.2
0.4
0.6
0.8
1.0
π

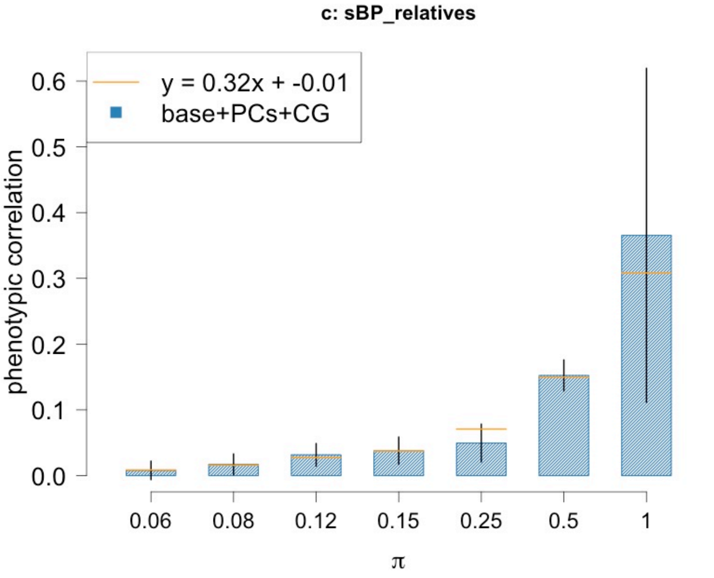
c: sBP_relatives
y = 0.32x + -0.01
base+PCs+CG
phenotypic correlation
0.6
0.5
0.4
0.3
0.2
0.1
0.0
0.06
0.08
0.12
0.15
0.25
0.5
1
π

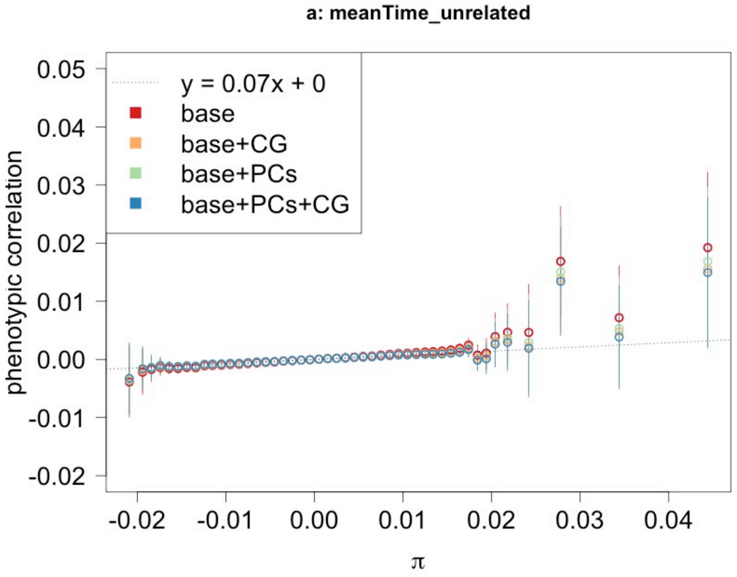
a: meanTime_unrelated
y = 0.07x + 0
base
base+CG
base+PCs
base+PCs+CG
phenotypic correlation
π

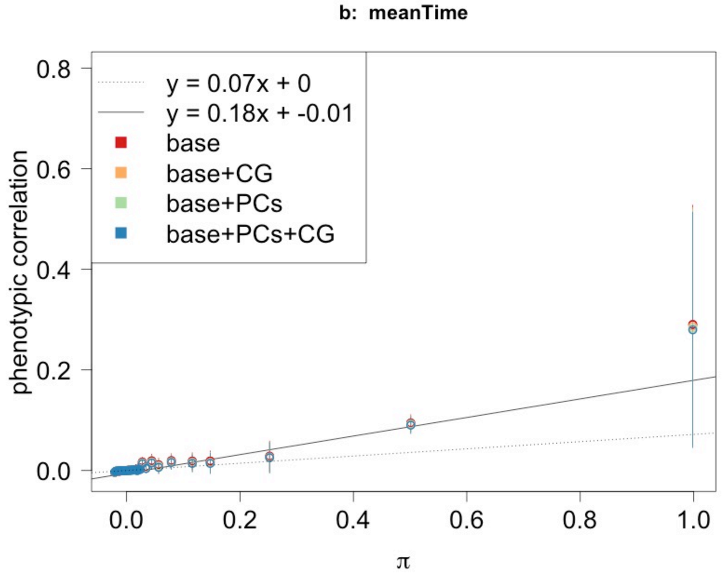
b: meanTime
y = 0.07x + 0
y = 0.18x + -0.01
base
base+CG
base+PCs
base+PCs+CG
phenotypic correlation
π

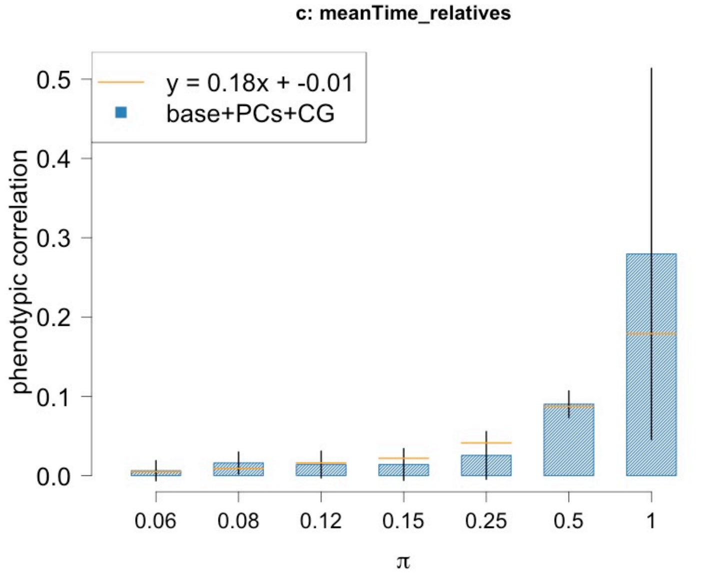
c: meanTime_relatives
y = 0.18x + -0.01
base+PCs+CG
phenotypic correlation
π

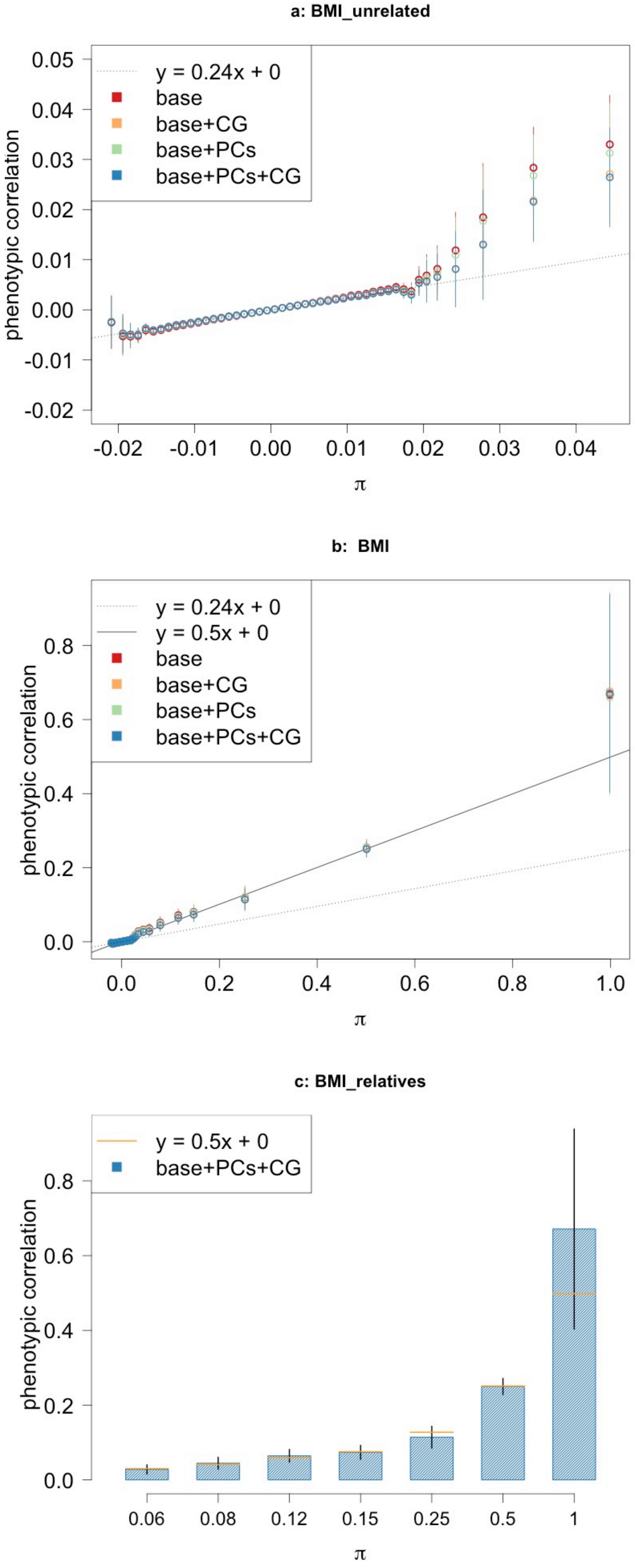
a: BMI_unrelated
y = 0.24x + 0
base
base+CG
base+PCs
base+PCs+CG
phenotypic correlation
0.05
0.04
0.03
0.02
0.01
0.00
-0.01
-0.02
-0.02
-0.01
0.00
0.01
0.02
0.03
0.04
π
b: BMI
y = 0.24x + 0
y = 0.5x + 0
base
base+CG
base+PCs
base+PCs+CG
phenotypic correlation
0.8
0.6
0.4
0.2
0.0
0.0
0.2
0.4
0.6
0.8
1.0
π
c: BMI_relatives
y = 0.5x + 0
base+PCs+CG
phenotypic correlation
0.8
0.6
0.4
0.2
0.0
0.06
0.08
0.12
0.15
0.25
0.5
1
π

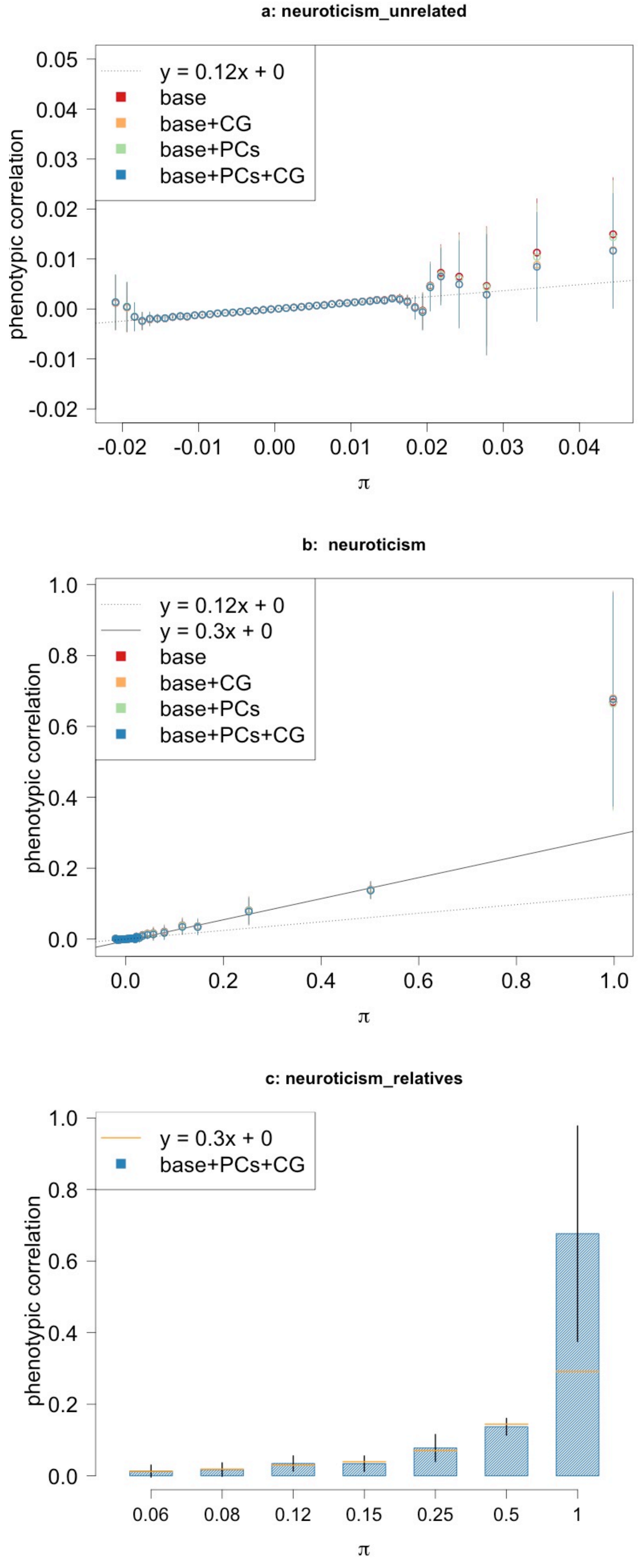
a: neuroticism_unrelated
y = 0.12x + 0
base
base+CG
base+PCs
base+PCs+CG
phenotypic correlation
0.05
0.04
0.03
0.02
0.01
0.00
-0.01
-0.02
-0.02
-0.01
0.00
0.01
0.02
0.03
0.04
π
b: neuroticism
y = 0.12x + 0
y = 0.3x + 0
base
base+CG
base+PCs
base+PCs+CG
phenotypic correlation
1.0
0.8
0.6
0.4
0.2
0.0
0.0
0.2
0.4
0.6
0.8
1.0
π
c: neuroticism_relatives
y = 0.3x + 0
base+PCs+CG
phenotypic correlation
1.0
0.8
0.6
0.4
0.2
0.0
0.06
0.08
0.12
0.15
0.25
0.5
1
π

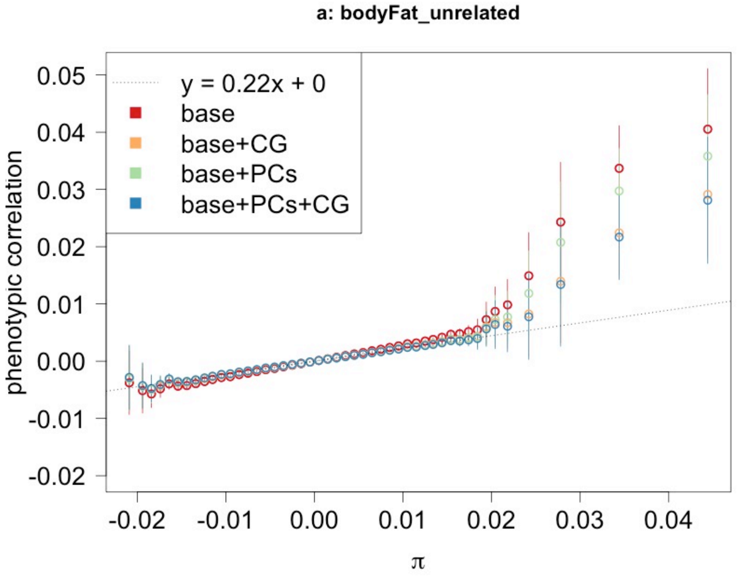

a: bodyFat_unrelated
y = 0.22x + 0
base
base+CG
base+PCs
base+PCs+CG
phenotypic correlation
π

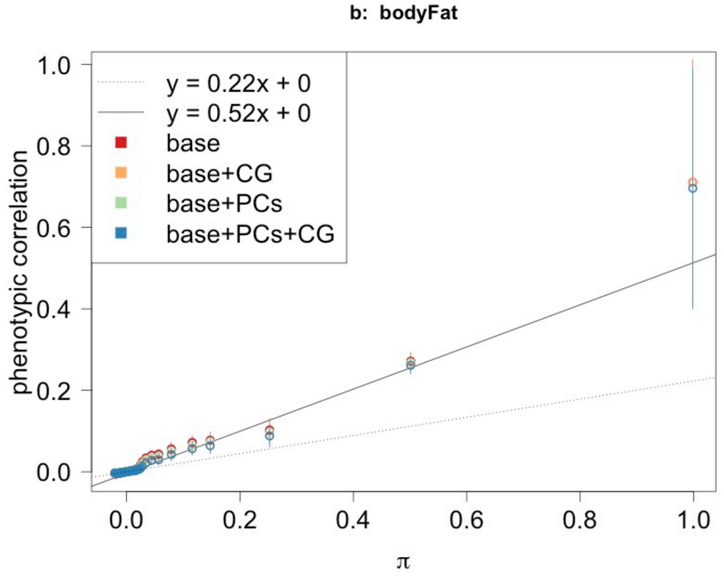

b: bodyFat
y = 0.22x + 0
y = 0.52x + 0
base
base+CG
base+PCs
base+PCs+CG
phenotypic correlation
π

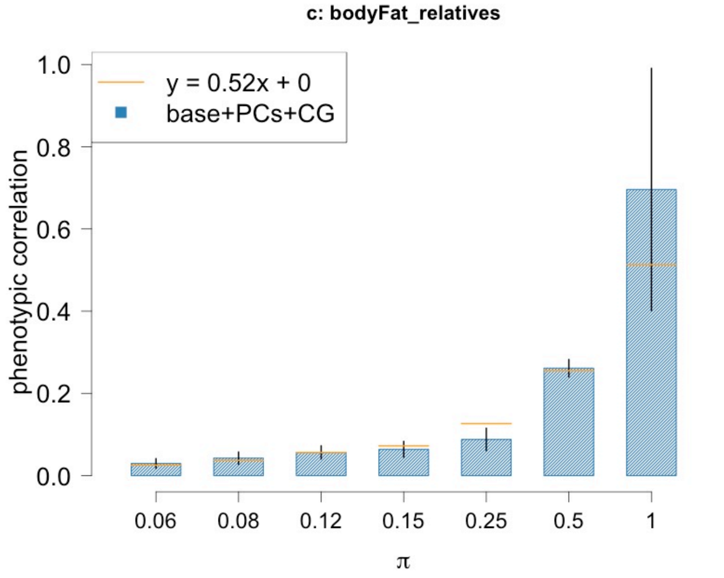

c: bodyFat_relatives
y = 0.52x + 0
base+PCs+CG
phenotypic correlation
π

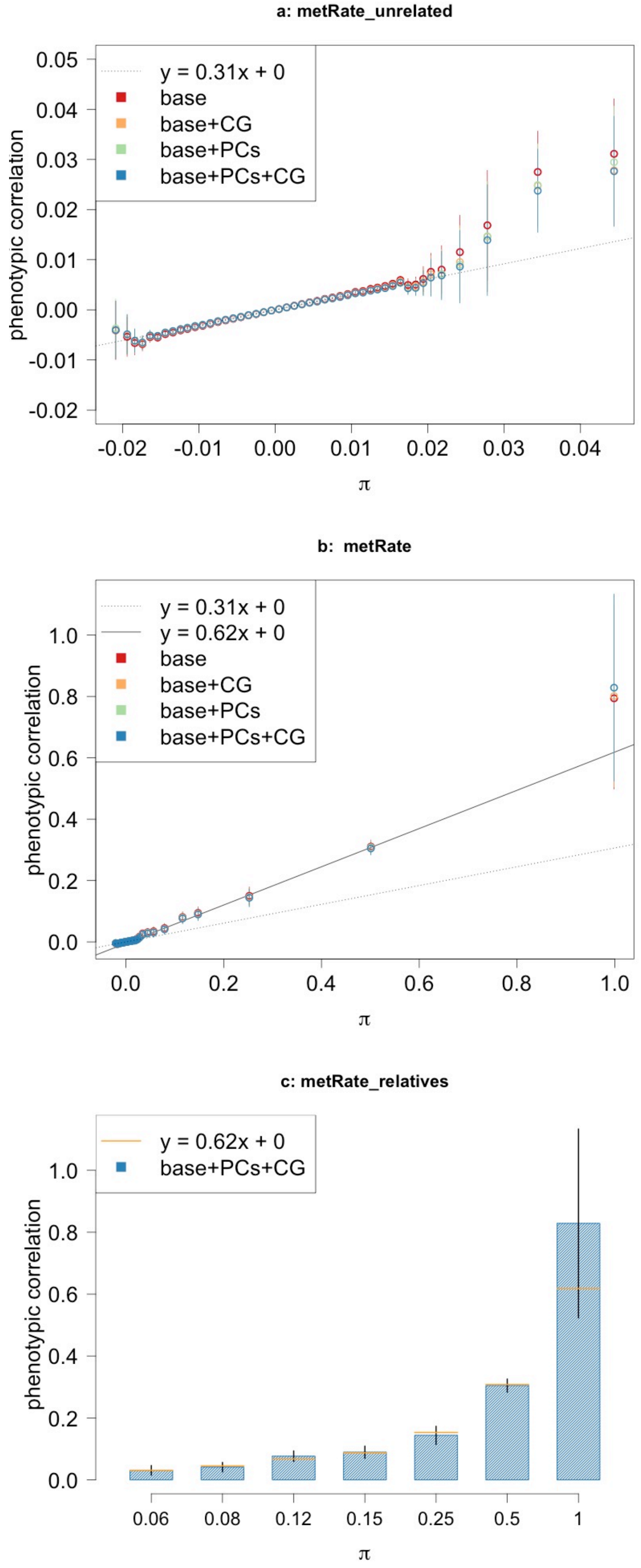
a: metRate_unrelated
y = 0.31x + 0
base
base+CG
base+PCs
base+PCs+CG
phenotypic correlation
π
b: metRate
y = 0.31x + 0
y = 0.62x + 0
base
base+CG
base+PCs
base+PCs+CG
phenotypic correlation
π
c: metRate_relatives
y = 0.62x + 0
base+PCs+CG
phenotypic correlation
π

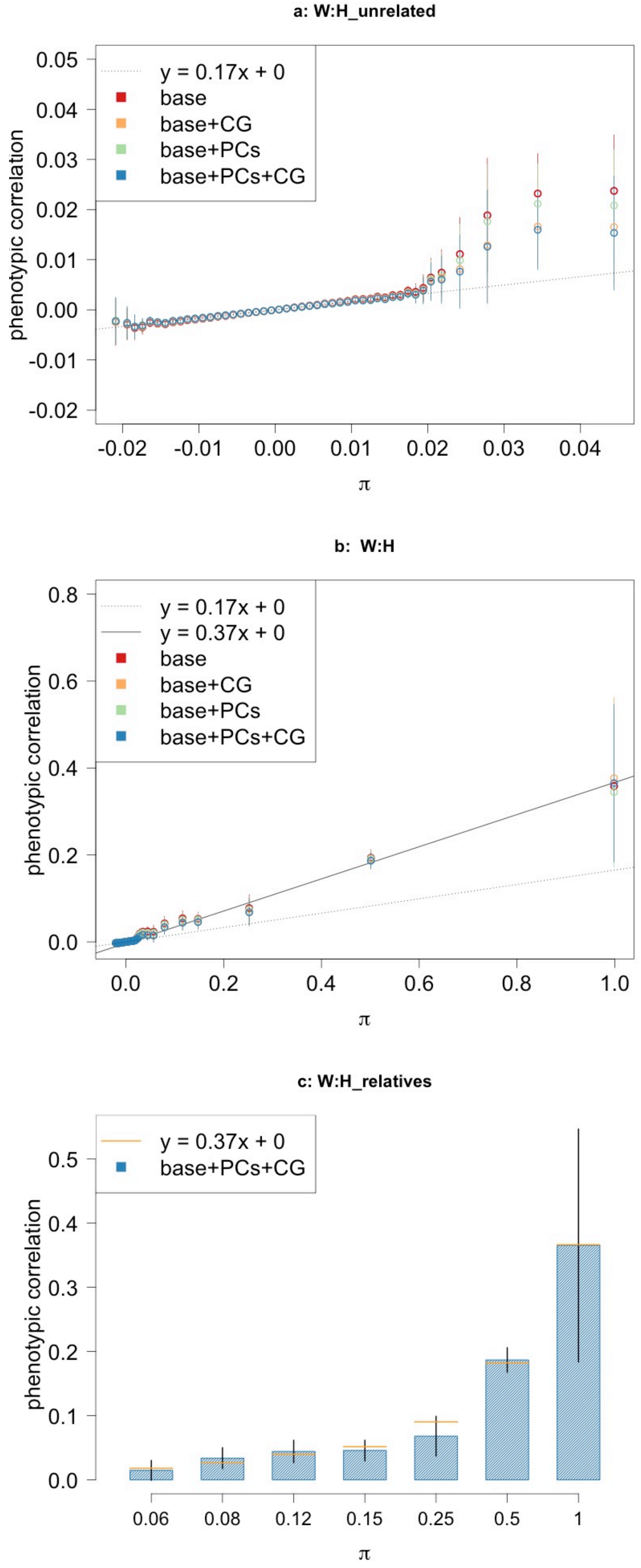

a: W:H_unrelated
y = 0.17x + 0
base
base+CG
base+PCs
base+PCs+CG
phenotypic correlation
0.05
0.04
0.03
0.02
0.01
0.00
-0.01
-0.02
-0.02
-0.01
0.00
0.01
0.02
0.03
0.04
π
b: W:H
y = 0.17x + 0
y = 0.37x + 0
base
base+CG
base+PCs
base+PCs+CG
phenotypic correlation
0.8
0.6
0.4
0.2
0.0
0.0
0.2
0.4
0.6
0.8
1.0
π
c: W:H_relatives
y = 0.37x + 0
base+PCs+CG
phenotypic correlation
0.5
0.4
0.3
0.2
0.1
0.0
0.06
0.08
0.12
0.15
0.25
0.5
1
π

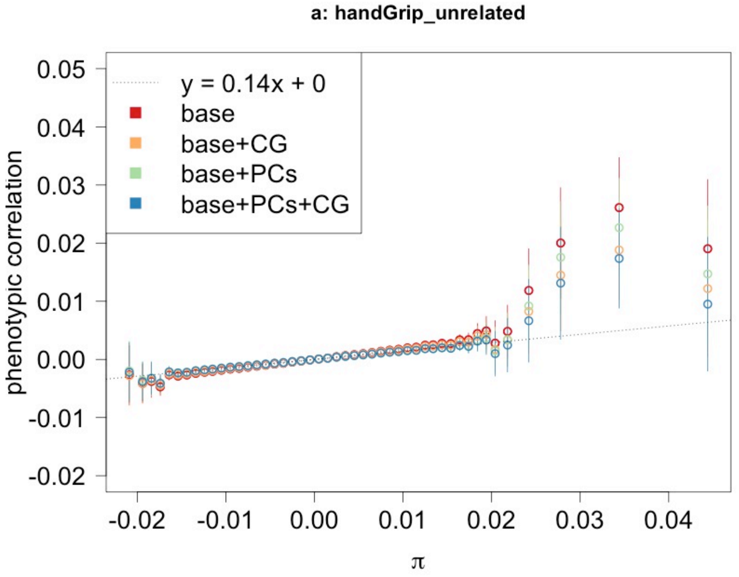
a: handGrip_unrelated
y = 0.14x + 0
base
base+CG
base+PCs
base+PCs+CG
phenotypic correlation
0.05
0.04
0.03
0.02
0.01
0.00
-0.01
-0.02
-0.02
-0.01
0.00
0.01
0.02
0.03
0.04
π

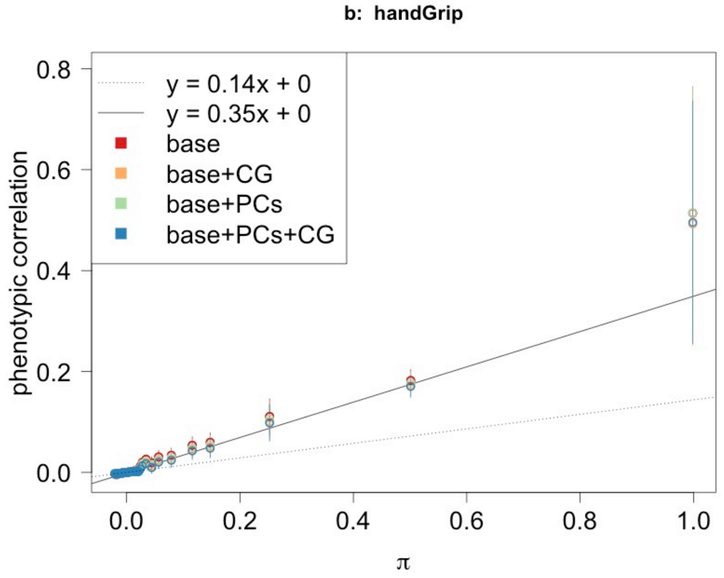
b: handGrip
y = 0.14x + 0
y = 0.35x + 0
base
base+CG
base+PCs
base+PCs+CG
phenotypic correlation
0.8
0.6
0.4
0.2
0.0
0.0
0.2
0.4
0.6
0.8
1.0
π

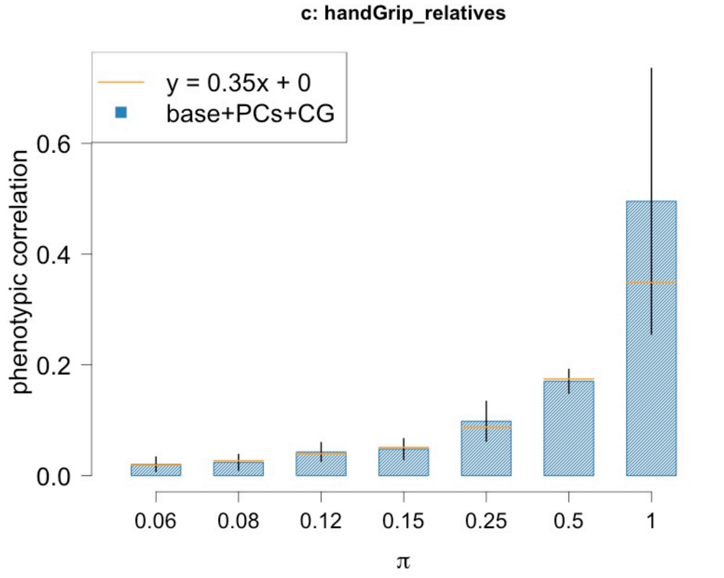
c: handGrip_relatives
y = 0.35x + 0
base+PCs+CG
phenotypic correlation
0.6
0.4
0.2
0.0
0.06
0.08
0.12
0.15
0.25
0.5
1
π

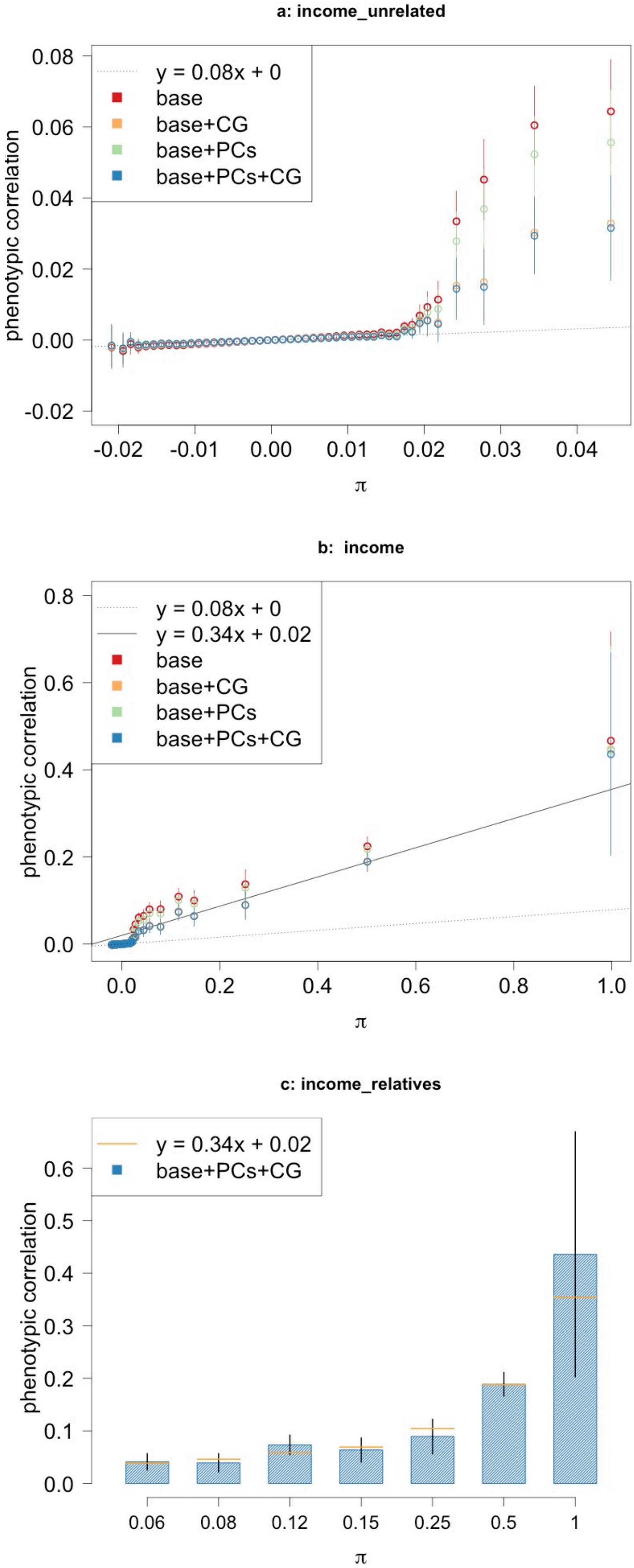
a: income_unrelated
y = 0.08x + 0
base
base+CG
base+PCs
base+PCs+CG
phenotypic correlation
π
b: income
y = 0.08x + 0
y = 0.34x + 0.02
base
base+CG
base+PCs
base+PCs+CG
phenotypic correlation
π
c: income_relatives
y = 0.34x + 0.02
base+PCs+CG
phenotypic correlation
π

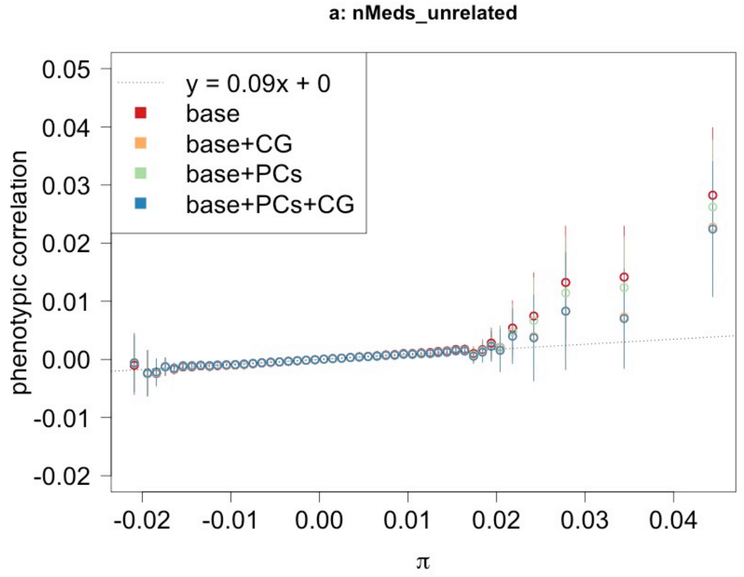
a: nMeds_unrelated
y = 0.09x + 0
base
base+CG
base+PCs
base+PCs+CG
phenotypic correlation
π

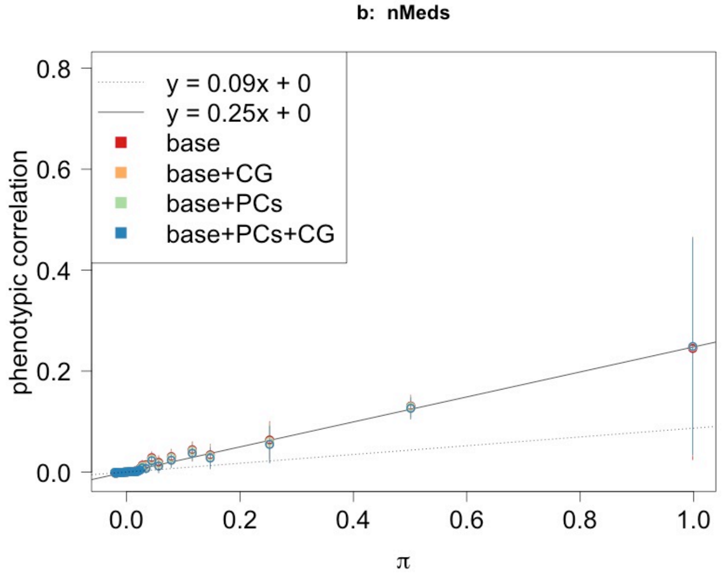
b: nMeds
y = 0.09x + 0
y = 0.25x + 0
base
base+CG
base+PCs
base+PCs+CG
phenotypic correlation
π

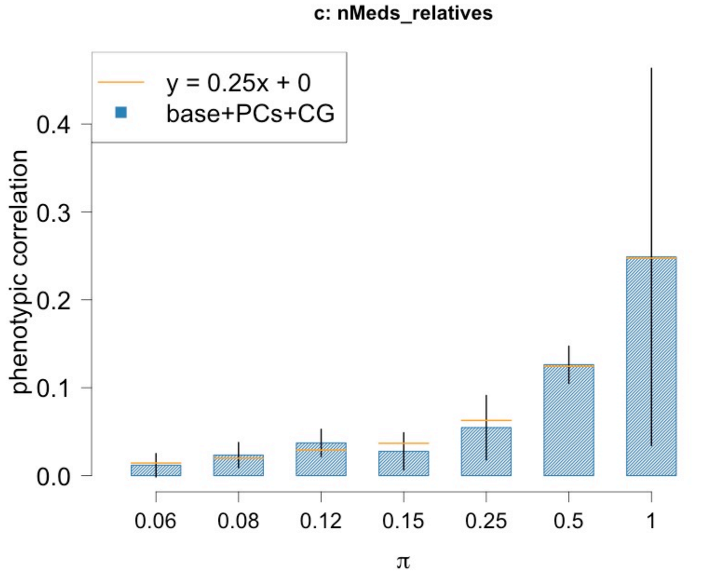
c: nMeds_relatives
y = 0.25x + 0
base+PCs+CG
phenotypic correlation
π

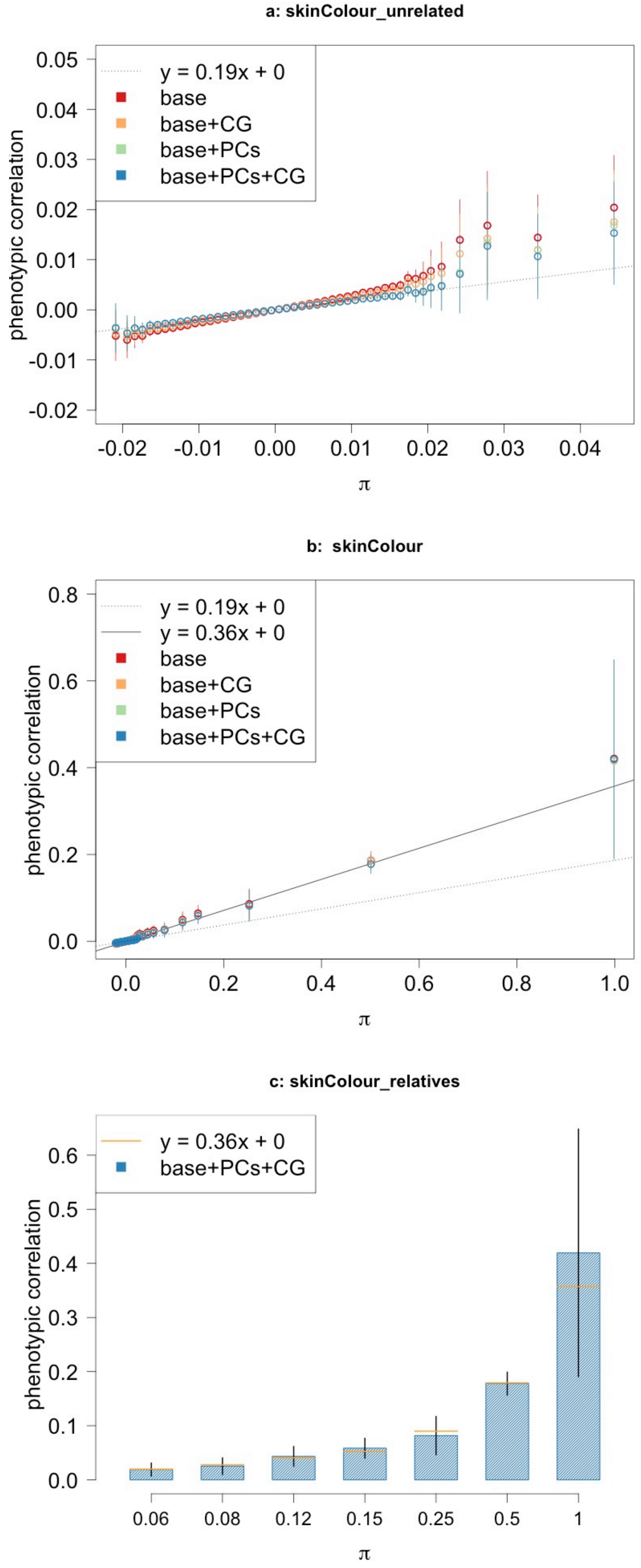
a: skinColour_unrelated
y = 0.19x + 0
base
base+CG
base+PCs
base+PCs+CG
phenotypic correlation
π
b: skinColour
y = 0.19x + 0
y = 0.36x + 0
base
base+CG
base+PCs
base+PCs+CG
phenotypic correlation
π
c: skinColour_relatives
y = 0.36x + 0
base+PCs+CG
phenotypic correlation
0.06
0.08
0.12
0.15
0.25
0.5
1
π

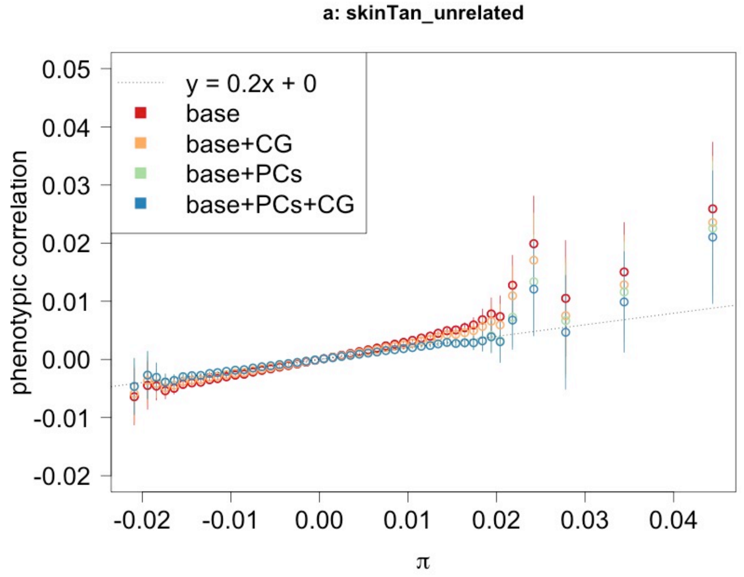
a: skinTan_unrelated
y = 0.2x + 0
base
base+CG
base+PCs
base+PCs+CG
phenotypic correlation
0.05
0.04
0.03
0.02
0.01
0.00
-0.01
-0.02
-0.02
-0.01
0.00
0.01
0.02
0.03
0.04
π

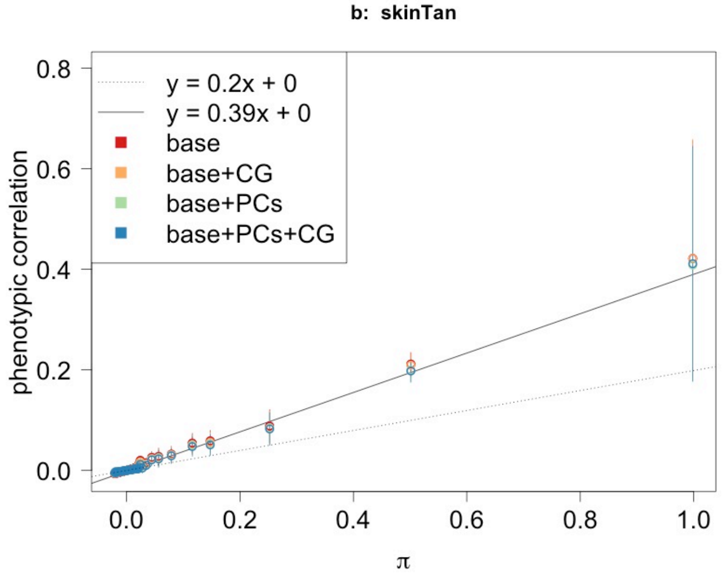
b: skinTan
y = 0.2x + 0
y = 0.39x + 0
base
base+CG
base+PCs
base+PCs+CG
phenotypic correlation
0.8
0.6
0.4
0.2
0.0
0.0
0.2
0.4
0.6
0.8
1.0
π

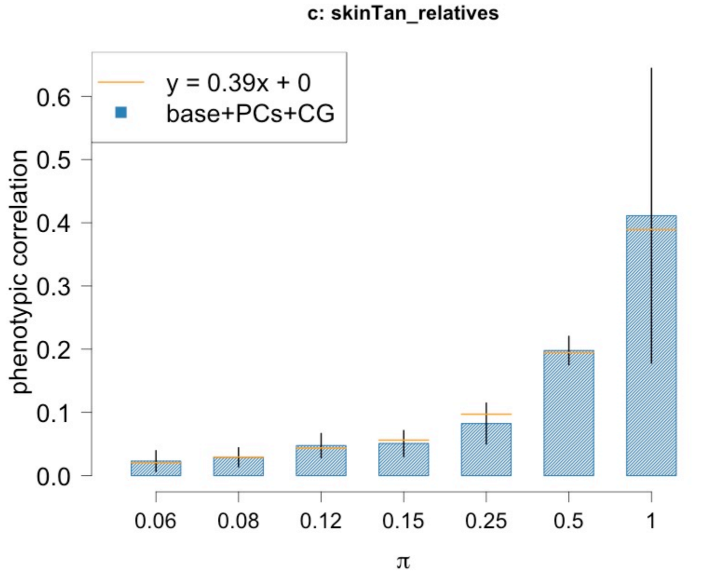
c: skinTan_relatives
y = 0.39x + 0
base+PCs+CG
phenotypic correlation
0.6
0.5
0.4
0.3
0.2
0.1
0.0
0.06
0.08
0.12
0.15
0.25
0.5
1
π

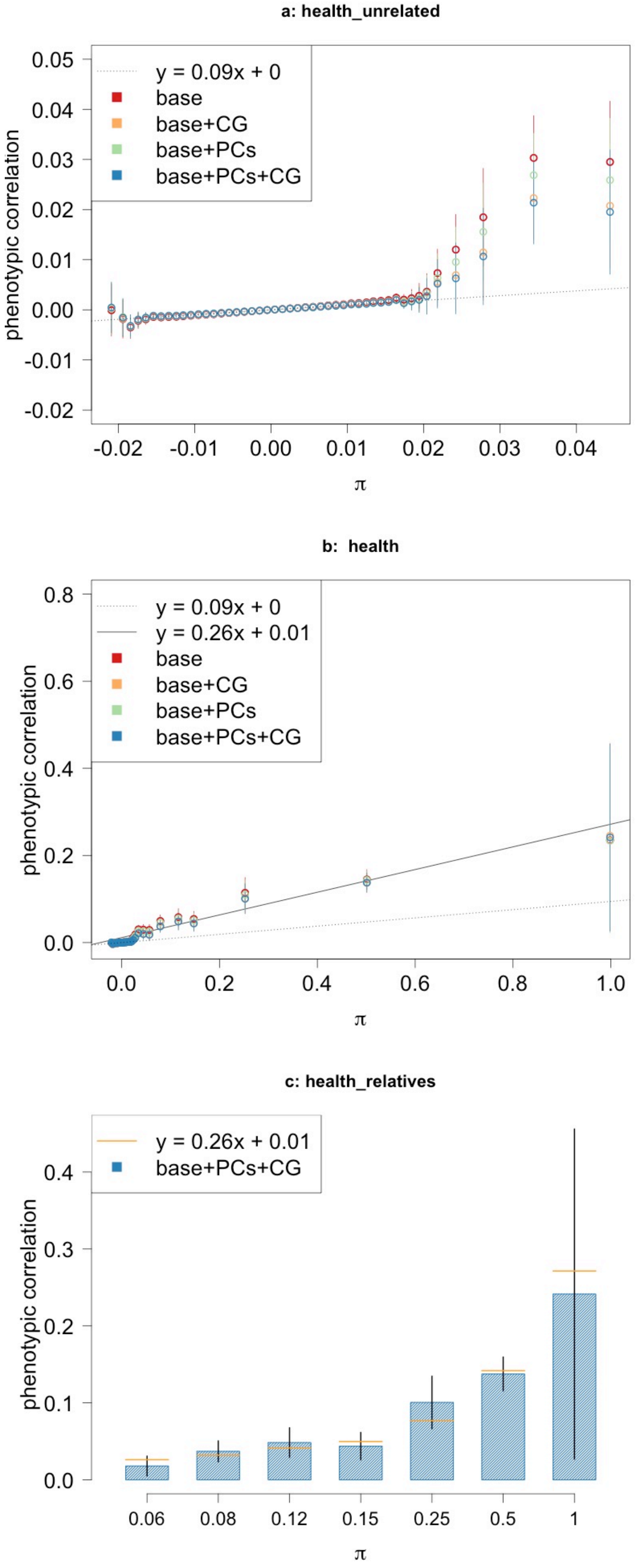
a: health_unrelated
y = 0.09x + 0
base
base+CG
base+PCs
base+PCs+CG
phenotypic correlation
0.05
0.04
0.03
0.02
0.01
0.00
-0.01
-0.02
-0.02
-0.01
0.00
0.01
0.02
0.03
0.04
π
b: health
y = 0.09x + 0
y = 0.26x + 0.01
base
base+CG
base+PCs
base+PCs+CG
phenotypic correlation
0.8
0.6
0.4
0.2
0.0
0.0
0.2
0.4
0.6
0.8
1.0
π
c: health_relatives
y = 0.26x + 0.01
base+PCs+CG
phenotypic correlation
0.4
0.3
0.2
0.1
0.0
0.06
0.08
0.12
0.15
0.25
0.5
1
π

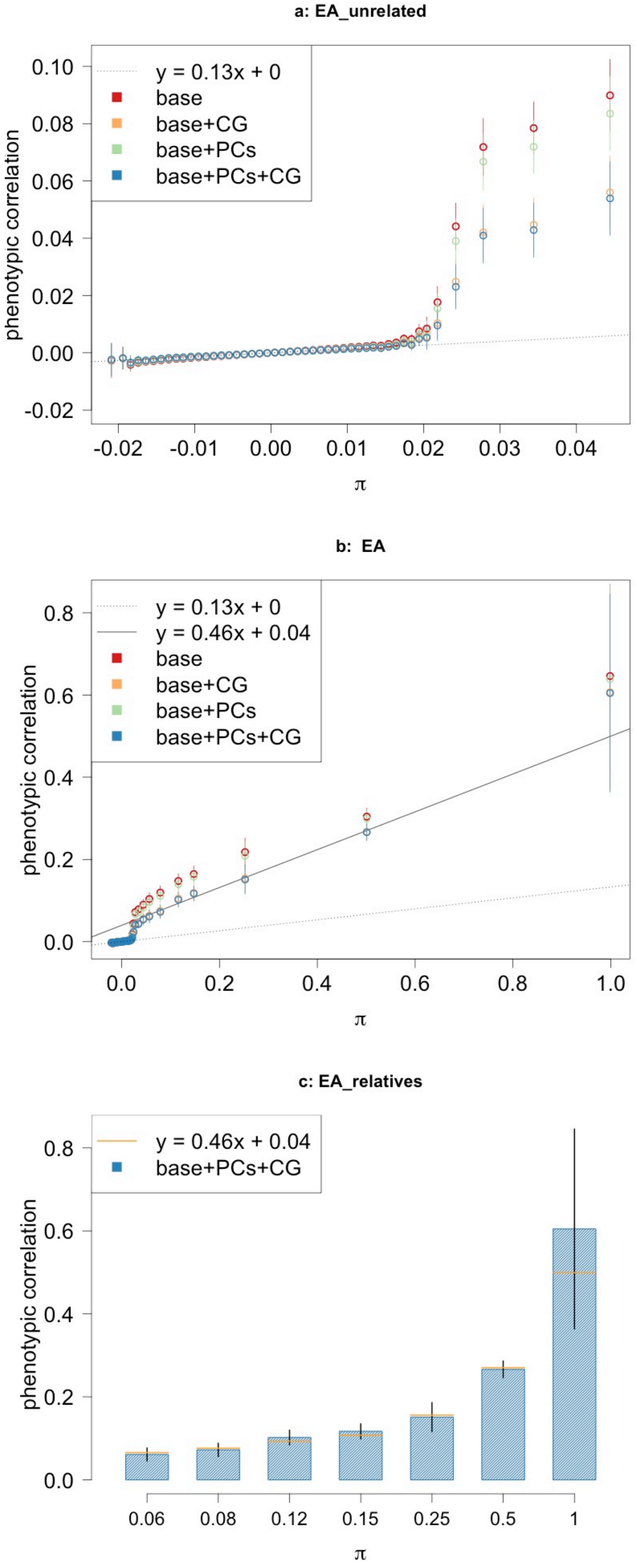
a: EA_unrelated
y = 0.13x + 0
base
base+CG
base+PCs
base+PCs+CG
phenotypic correlation
π
b: EA
y = 0.13x + 0
y = 0.46x + 0.04
base
base+CG
base+PCs
base+PCs+CG
phenotypic correlation
π
c: EA_relatives
y = 0.46x + 0.04
base+PCs+CG
phenotypic correlation
0.06
0.08
0.12
0.15
0.25
0.5
1
π

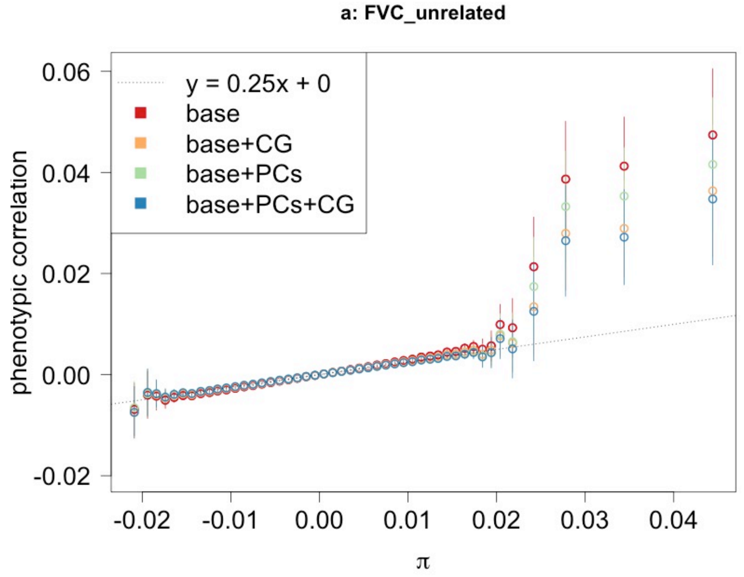
a: FVC_unrelated
y = 0.25x + 0
base
base+CG
base+PCs
base+PCs+CG
phenotypic correlation
π

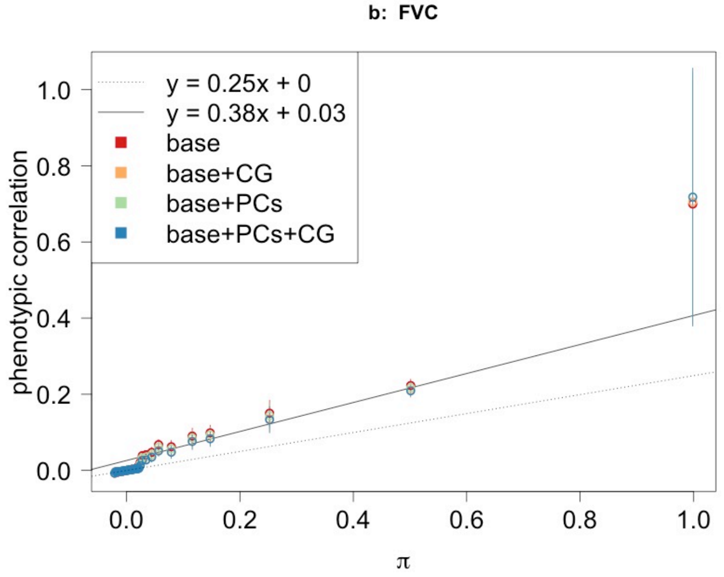
b: FVC
y = 0.25x + 0
y = 0.38x + 0.03
base
base+CG
base+PCs
base+PCs+CG
phenotypic correlation
π

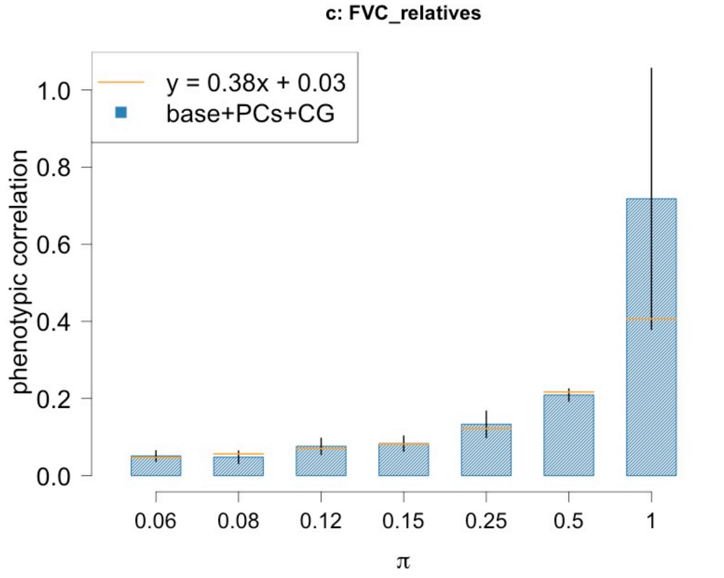
c: FVC_relatives
y = 0.38x + 0.03
base+PCs+CG
phenotypic correlation
π

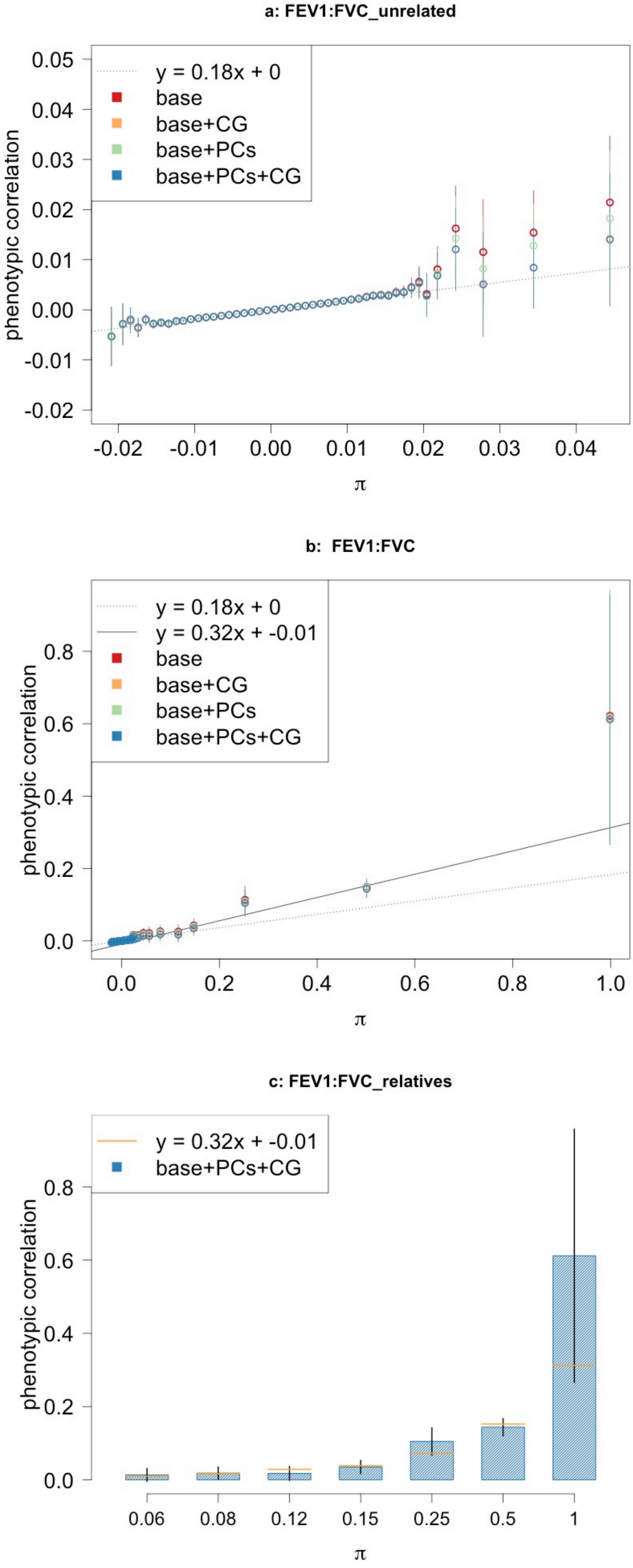

a: FEV1:FVC_unrelated
y = 0.18x + 0
base
base+CG
base+PCs
base+PCs+CG
phenotypic correlation
π
b: FEV1:FVC
y = 0.18x + 0
y = 0.32x + -0.01
base
base+CG
base+PCs
base+PCs+CG
phenotypic correlation
π
c: FEV1:FVC_relatives
y = 0.32x + -0.01
base+PCs+CG
phenotypic correlation
0.06
0.08
0.12
0.15
0.25
0.5
1
π

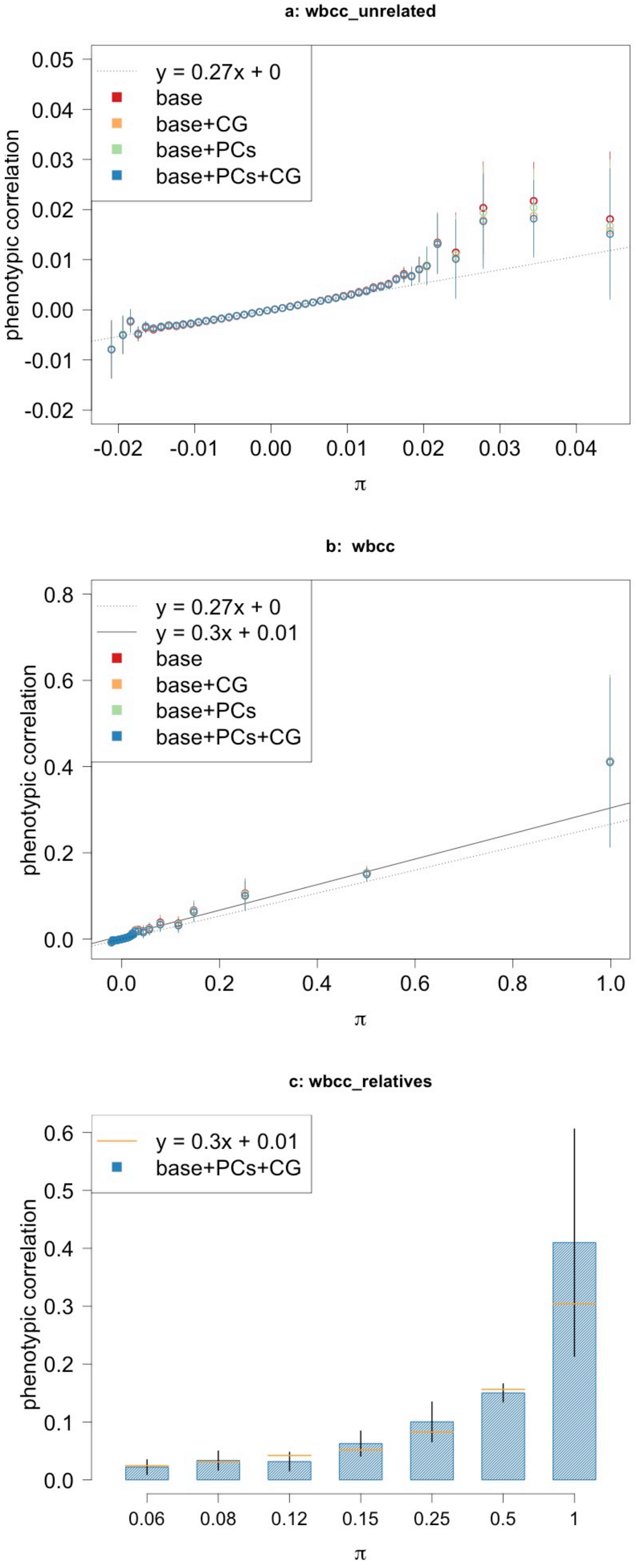
a: wbcc_unrelated
y = 0.27x + 0
base
base+CG
base+PCs
base+PCs+CG
phenotypic correlation
π
b: wbcc
y = 0.27x + 0
y = 0.3x + 0.01
base
base+CG
base+PCs
base+PCs+CG
phenotypic correlation
π
c: wbcc_relatives
y = 0.3x + 0.01
base+PCs+CG
phenotypic correlation
0.06
0.08
0.12
0.15
0.25
0.5
1
π

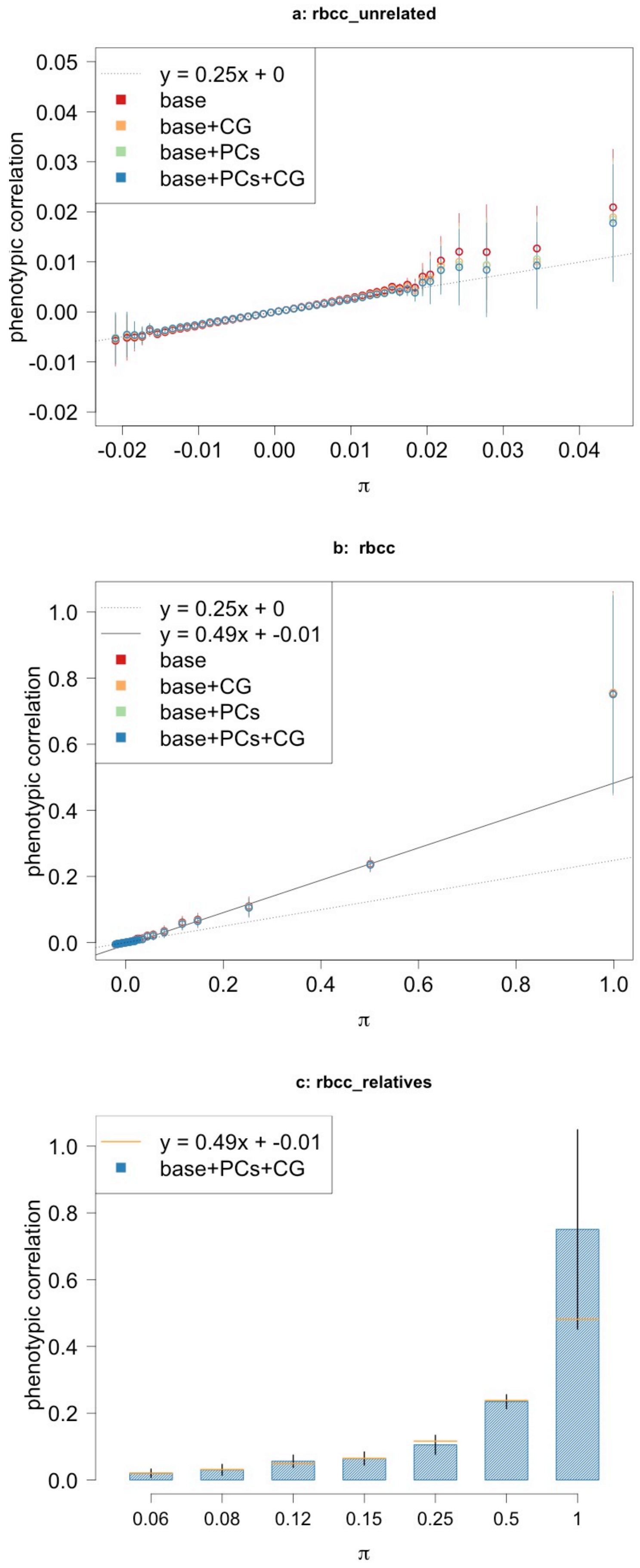

a: rbcc_unrelated
y = 0.25x + 0
base
base+CG
base+PCs
base+PCs+CG
phenotypic correlation
π
b: rbcc
y = 0.25x + 0
y = 0.49x + -0.01
base
base+CG
base+PCs
base+PCs+CG
phenotypic correlation
π
c: rbcc_relatives
y = 0.49x + -0.01
base+PCs+CG
phenotypic correlation
π

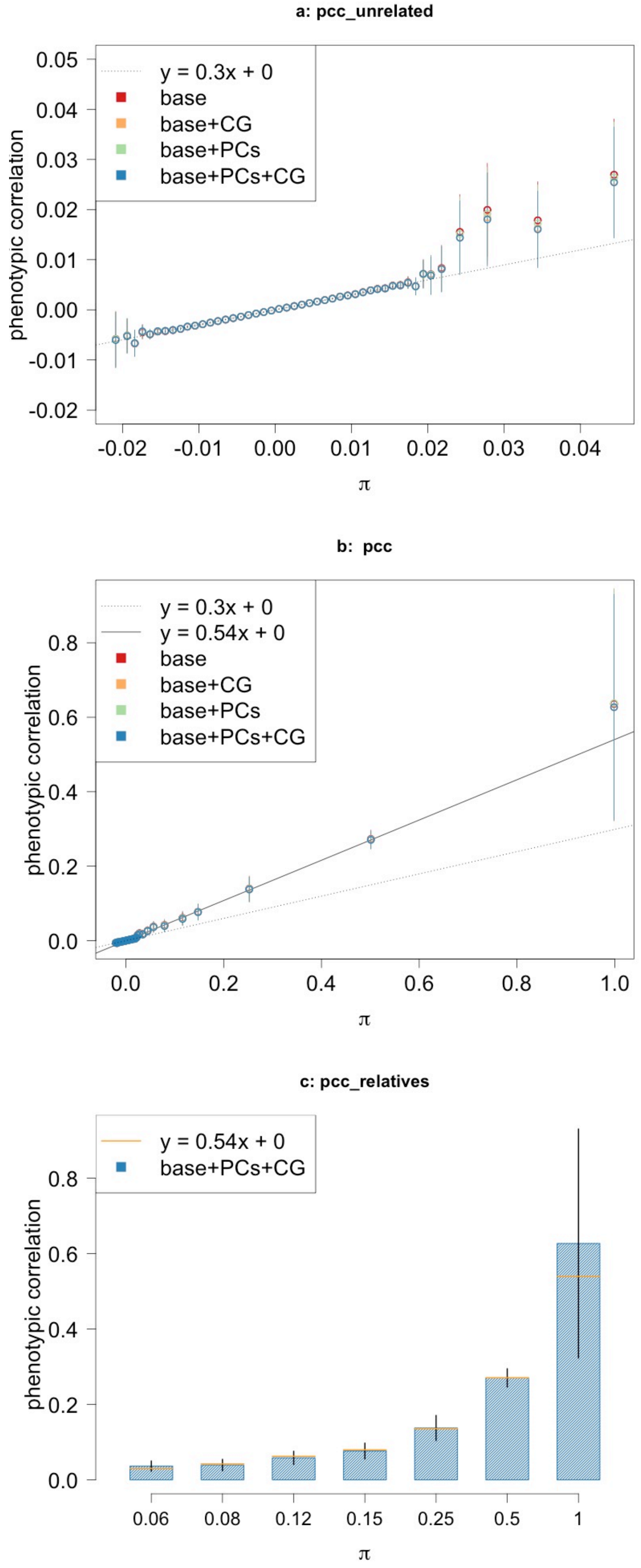
a: pcc_unrelated
y = 0.3x + 0
base
base+CG
base+PCs
base+PCs+CG
phenotypic correlation
0.05
0.04
0.03
0.02
0.01
0.00
-0.01
-0.02
-0.02
-0.01
0.00
0.01
0.02
0.03
0.04
π
b: pcc
y = 0.3x + 0
y = 0.54x + 0
base
base+CG
base+PCs
base+PCs+CG
phenotypic correlation
0.8
0.6
0.4
0.2
0.0
0.0
0.2
0.4
0.6
0.8
1.0
π
c: pcc_relatives
y = 0.54x + 0
base+PCs+CG
phenotypic correlation
0.8
0.6
0.4
0.2
0.0
0.06
0.08
0.12
0.15
0.25
0.5
1
π

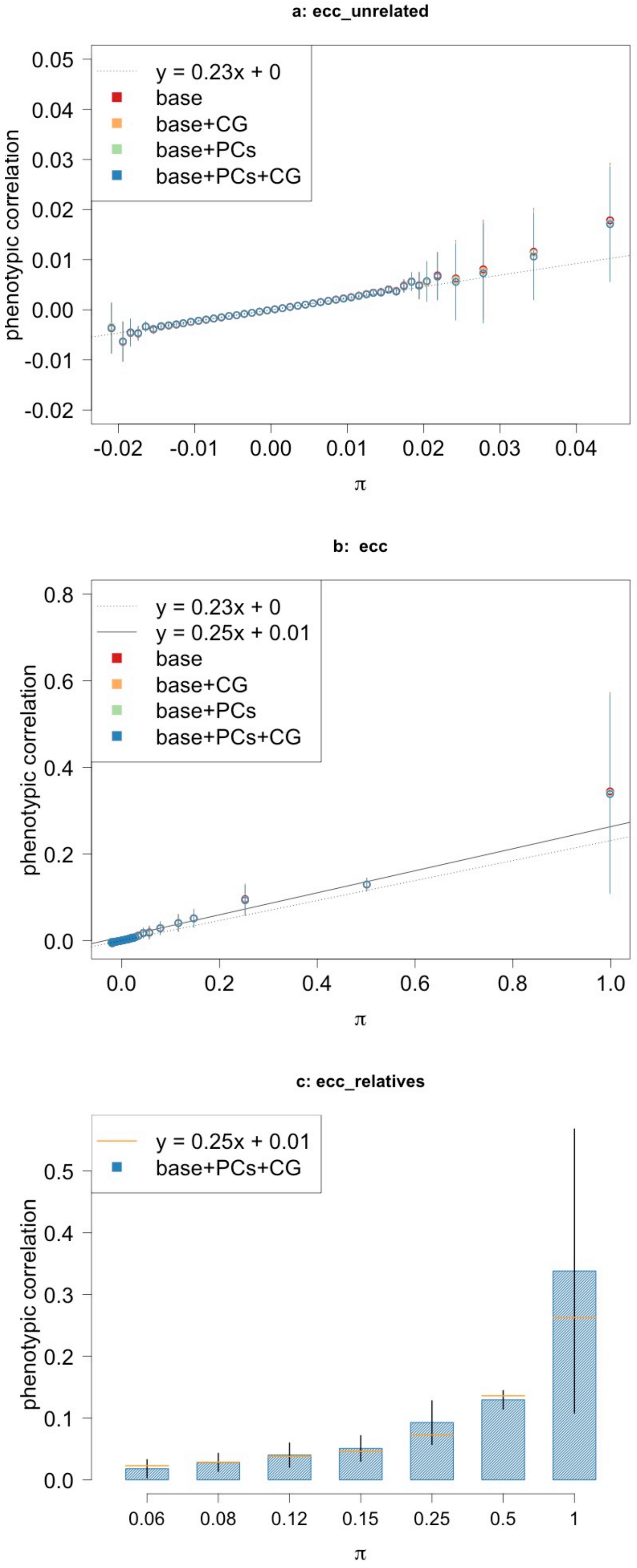
a: ecc_unrelated
y = 0.23x + 0
base
base+CG
base+PCs
base+PCs+CG
phenotypic correlation
0.05
0.04
0.03
0.02
0.01
0.00
-0.01
-0.02
-0.02
-0.01
0.00
0.01
0.02
0.03
0.04
π
b: ecc
y = 0.23x + 0
y = 0.25x + 0.01
base
base+CG
base+PCs
base+PCs+CG
phenotypic correlation
0.8
0.6
0.4
0.2
0.0
0.0
0.2
0.4
0.6
0.8
1.0
π
c: ecc_relatives
y = 0.25x + 0.01
base+PCs+CG
phenotypic correlation
0.5
0.4
0.3
0.2
0.1
0.0
0.06
0.08
0.12
0.15
0.25
0.5
1
π

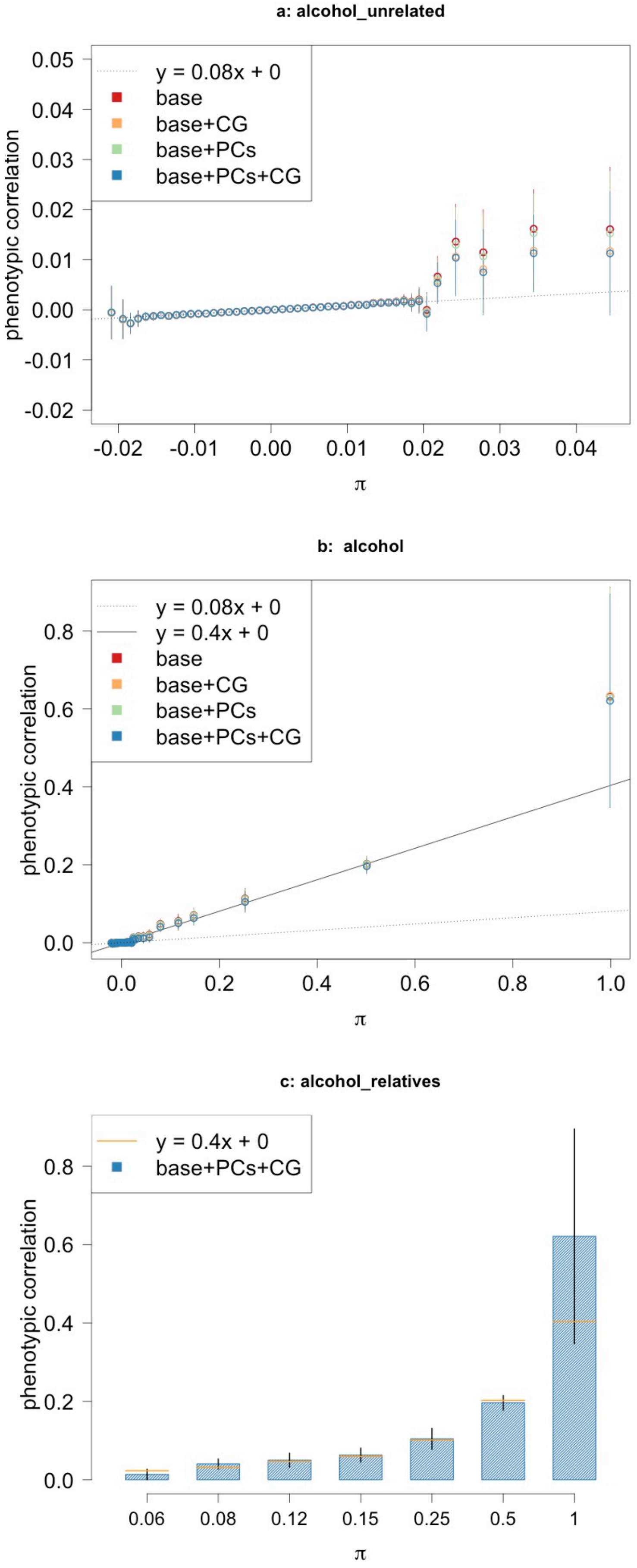
a: alcohol_unrelated
y = 0.08x + 0
base
base+CG
base+PCs
base+PCs+CG
phenotypic correlation
0.05
0.04
0.03
0.02
0.01
0.00
-0.01
-0.02
-0.02
-0.01
0.00
0.01
0.02
0.03
0.04
π
b: alcohol
y = 0.08x + 0
y = 0.4x + 0
base
base+CG
base+PCs
base+PCs+CG
phenotypic correlation
0.8
0.6
0.4
0.2
0.0
0.0
0.2
0.4
0.6
0.8
1.0
π
c: alcohol_relatives
y = 0.4x + 0
base+PCs+CG
phenotypic correlation
0.8
0.6
0.4
0.2
0.0
0.06
0.08
0.12
0.15
0.25
0.5
1
π

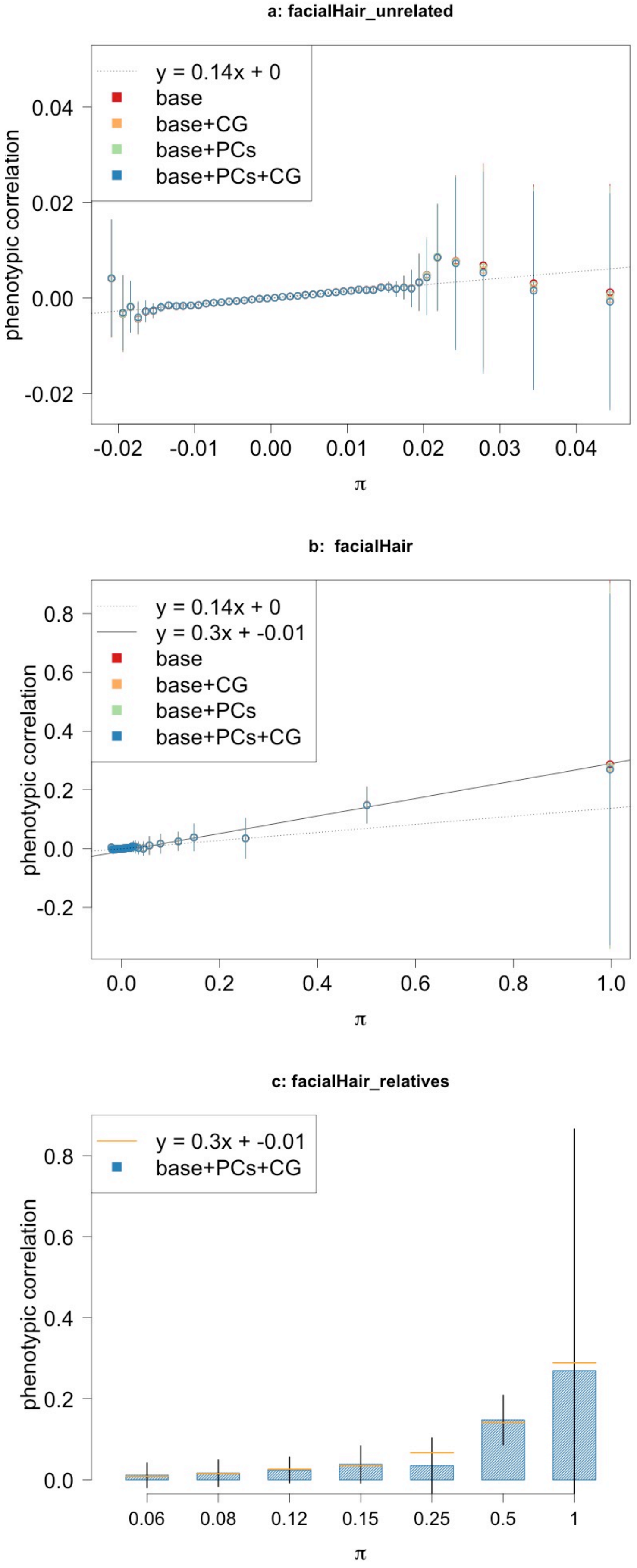
a: facialHair_unrelated
y = 0.14x + 0
base
base+CG
base+PCs
base+PCs+CG
phenotypic correlation
0.04
0.02
0.00
-0.02
-0.02 -0.01 0.00 0.01 0.02 0.03 0.04
π
b: facialHair
y = 0.14x + 0
y = 0.3x + -0.01
base
base+CG
base+PCs
base+PCs+CG
phenotypic correlation
0.8
0.6
0.4
0.2
0.0
-0.2
0.0 0.2 0.4 0.6 0.8 1.0
π
c: facialHair_relatives
y = 0.3x + -0.01
base+PCs+CG
phenotypic correlation
0.8
0.6
0.4
0.2
0.0
0.06 0.08 0.12 0.15 0.25 0.5 1
π

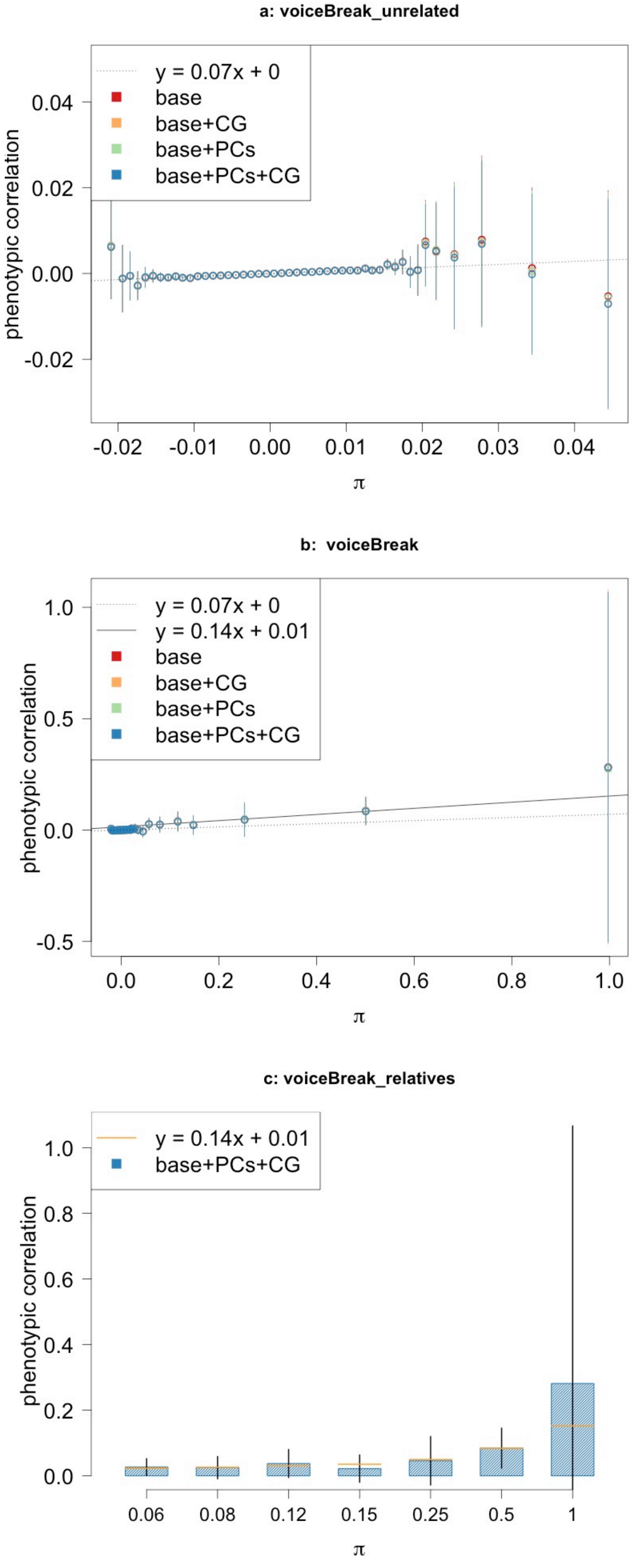
a: voiceBreak_unrelated
y = 0.07x + 0
base
base+CG
base+PCs
base+PCs+CG
phenotypic correlation
0.04
0.02
0.00
-0.02
-0.02
-0.01
0.00
0.01
0.02
0.03
0.04
π
b: voiceBreak
y = 0.07x + 0
y = 0.14x + 0.01
base
base+CG
base+PCs
base+PCs+CG
phenotypic correlation
1.0
0.5
0.0
-0.5
0.0
0.2
0.4
0.6
0.8
1.0
π
c: voiceBreak_relatives
y = 0.14x + 0.01
base+PCs+CG
phenotypic correlation
1.0
0.8
0.6
0.4
0.2
0.0
0.06
0.08
0.12
0.15
0.25
0.5
1
π

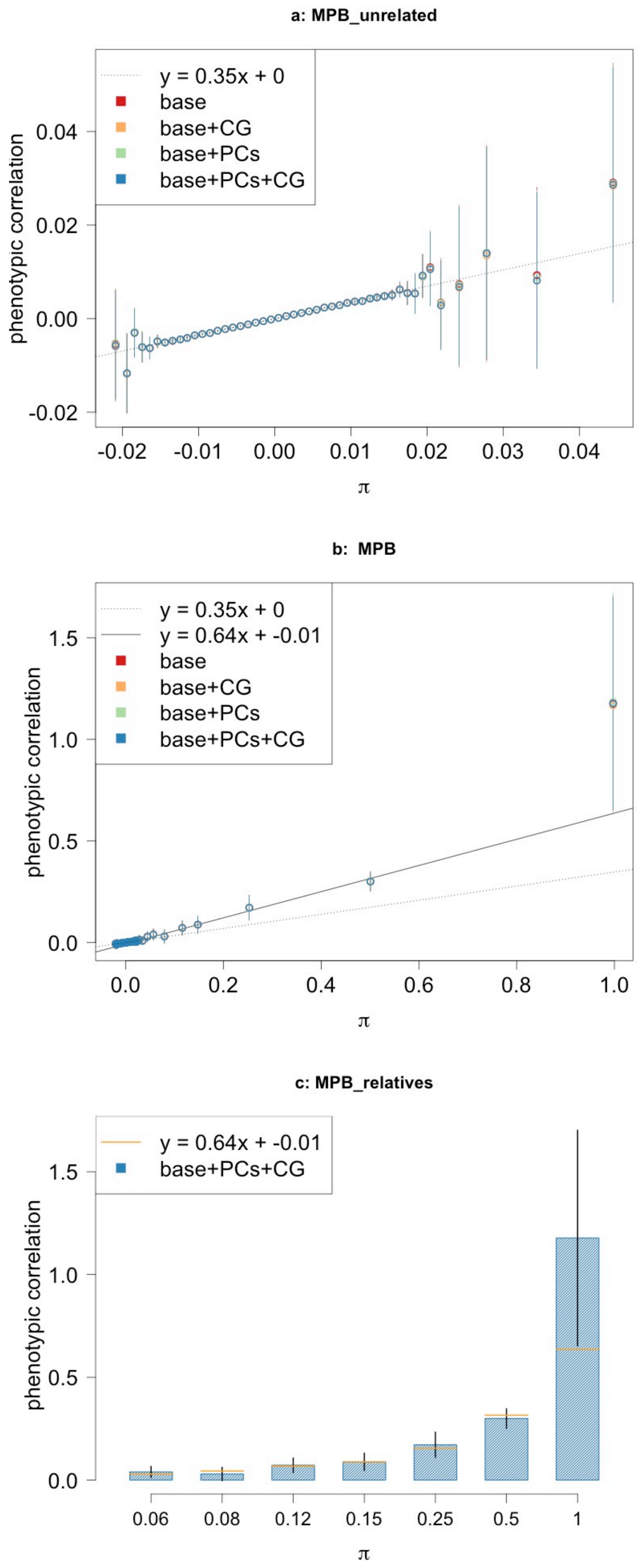
a: MPB_unrelated
y = 0.35x + 0
base
base+CG
base+PCs
base+PCs+CG
phenotypic correlation
0.04
0.02
0.00
-0.02
-0.02
-0.01
0.00
0.01
0.02
0.03
0.04
π
b: MPB
y = 0.35x + 0
y = 0.64x + -0.01
base
base+CG
base+PCs
base+PCs+CG
phenotypic correlation
1.5
1.0
0.5
0.0
0.0
0.2
0.4
0.6
0.8
1.0
π
c: MPB_relatives
y = 0.64x + -0.01
base+PCs+CG
phenotypic correlation
1.5
1.0
0.5
0.0
0.06
0.08
0.12
0.15
0.25
0.5
1
π

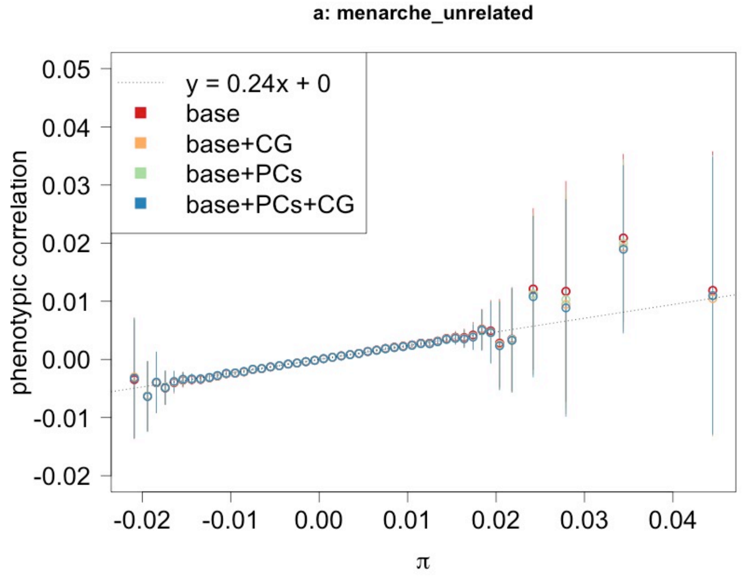
a: menarche_unrelated
y = 0.24x + 0
base
base+CG
base+PCs
base+PCs+CG
phenotypic correlation
π

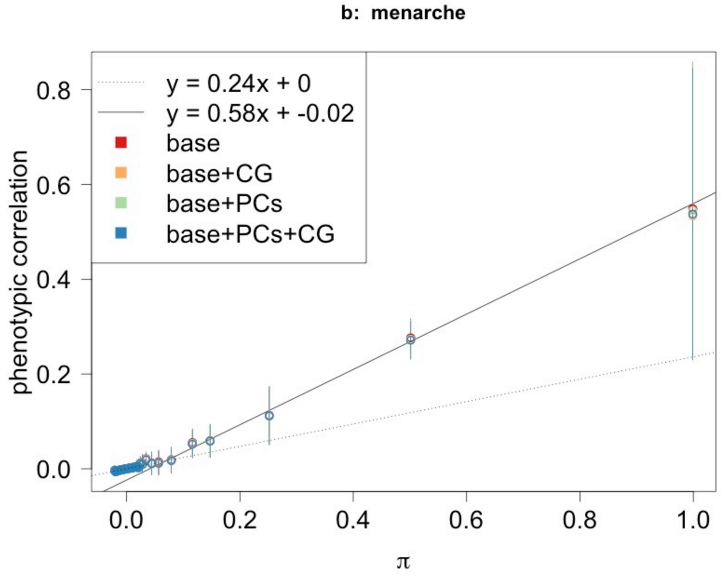
b: menarche
y = 0.24x + 0
y = 0.58x + -0.02
base
base+CG
base+PCs
base+PCs+CG
phenotypic correlation
π

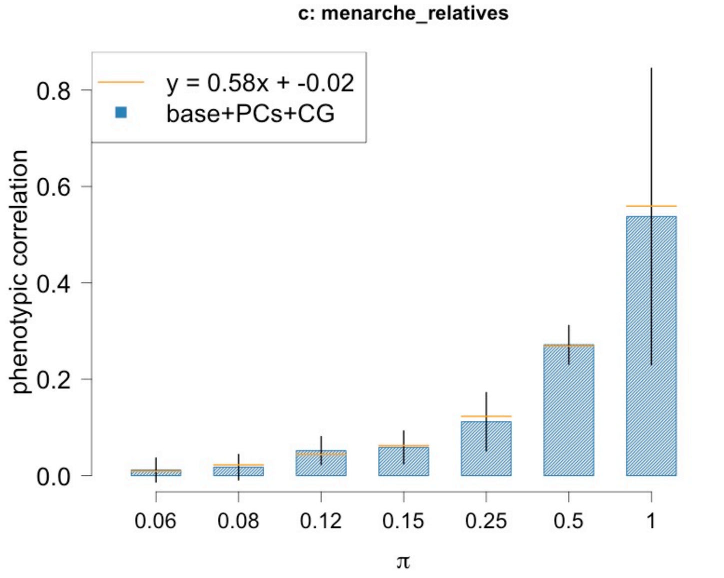
c: menarche_relatives
y = 0.58x + -0.02
base+PCs+CG
phenotypic correlation
0.06
0.08
0.12
0.15
0.25
0.5
1
π

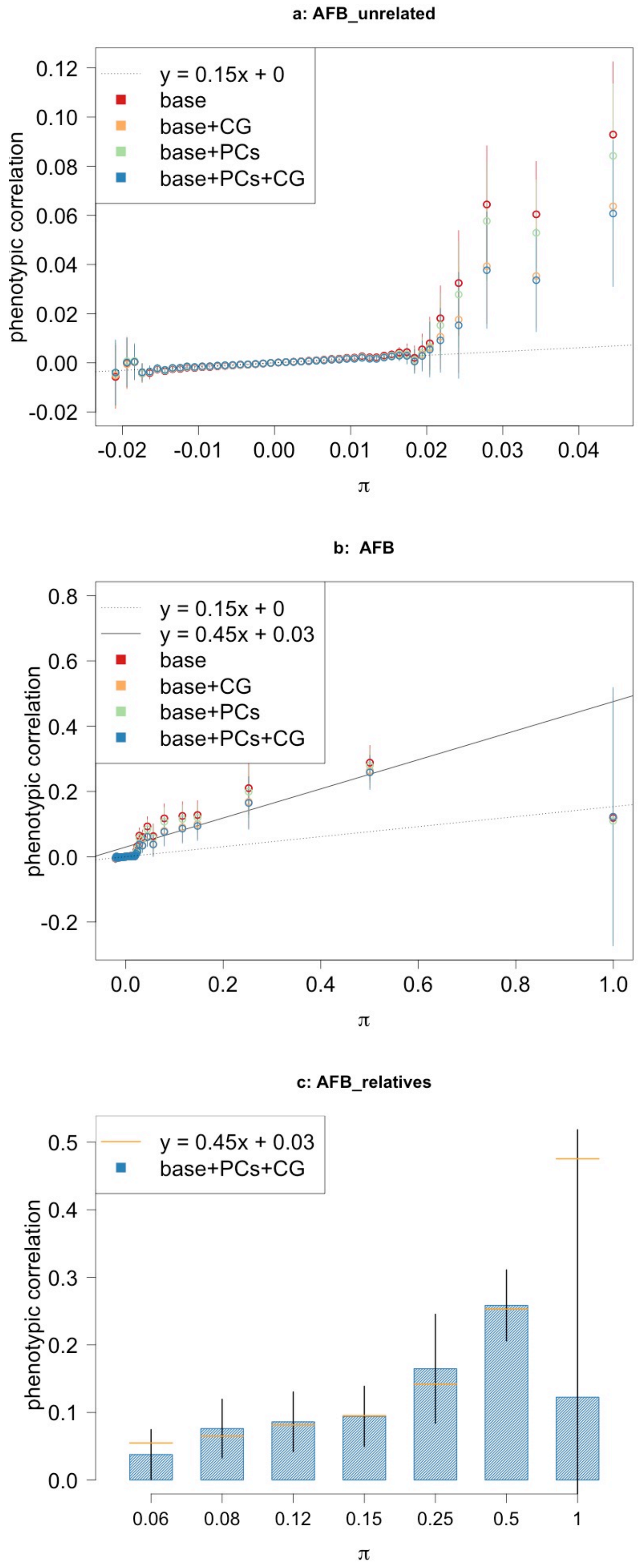
a: AFB_unrelated
y = 0.15x + 0
base
base+CG
base+PCs
base+PCs+CG
phenotypic correlation
π
b: AFB
y = 0.15x + 0
y = 0.45x + 0.03
base
base+CG
base+PCs
base+PCs+CG
phenotypic correlation
π
c: AFB_relatives
y = 0.45x + 0.03
base+PCs+CG
phenotypic correlation
0.06
0.08
0.12
0.15
0.25
0.5
1
π

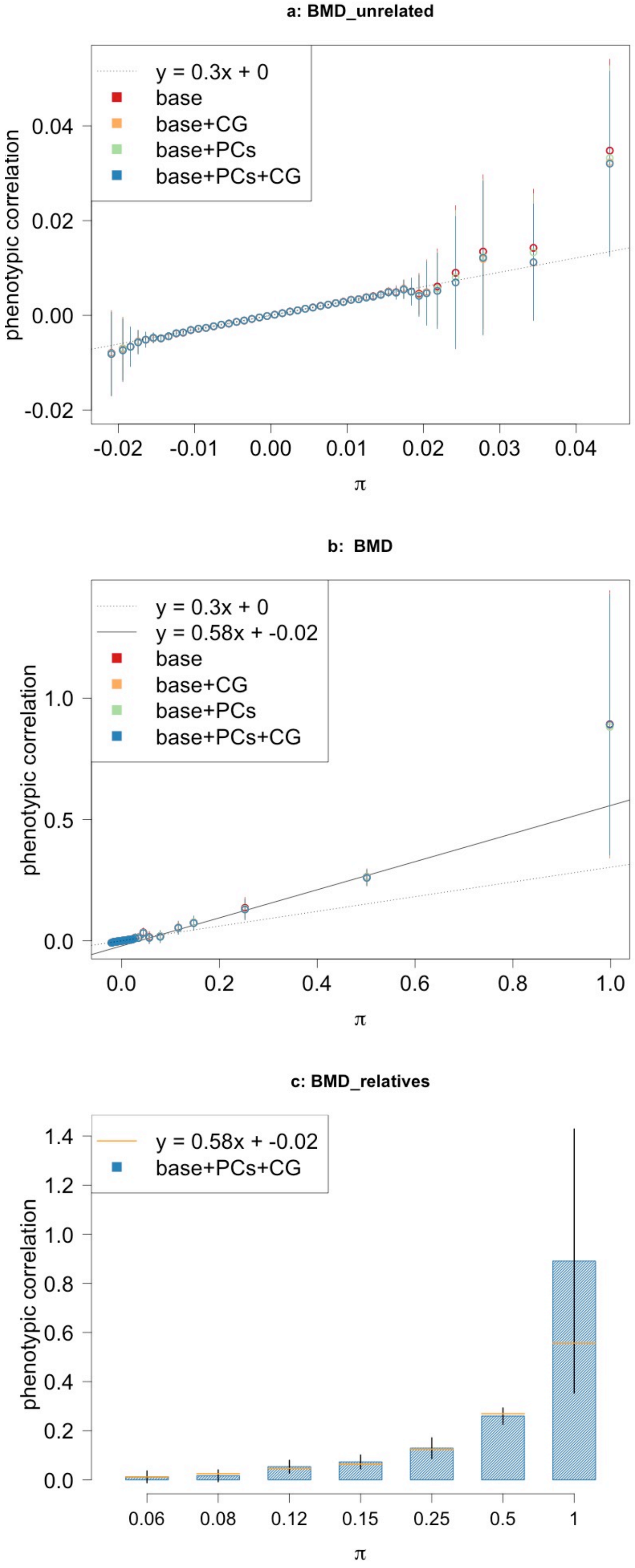
a: BMD_unrelated
y = 0.3x + 0
base
base+CG
base+PCs
base+PCs+CG
phenotypic correlation
0.04
0.02
0.00
-0.02
-0.02
-0.01
0.00
0.01
0.02
0.03
0.04
π
b: BMD
y = 0.3x + 0
y = 0.58x + -0.02
base
base+CG
base+PCs
base+PCs+CG
phenotypic correlation
1.0
0.5
0.0
0.0
0.2
0.4
0.6
0.8
1.0
π
c: BMD_relatives
y = 0.58x + -0.02
base+PCs+CG
phenotypic correlation
1.4
1.2
1.0
0.8
0.6
0.4
0.2
0.0
0.06
0.08
0.12
0.15
0.25
0.5
1
π

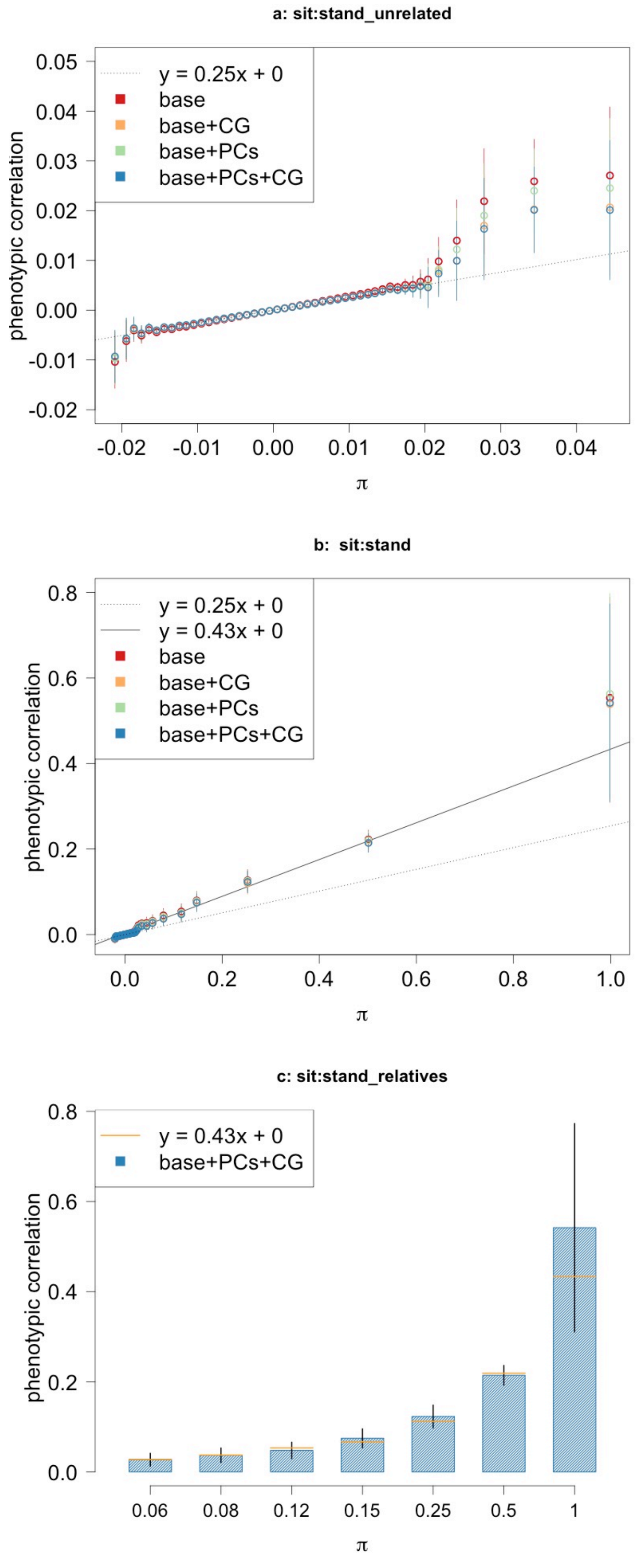
a: sit:stand_unrelated
y = 0.25x + 0
base
base+CG
base+PCs
base+PCs+CG
phenotypic correlation
0.05
0.04
0.03
0.02
0.01
0.00
-0.01
-0.02
-0.02
-0.01
0.00
0.01
0.02
0.03
0.04
π
b: sit:stand
y = 0.25x + 0
y = 0.43x + 0
base
base+CG
base+PCs
base+PCs+CG
phenotypic correlation
0.8
0.6
0.4
0.2
0.0
0.0
0.2
0.4
0.6
0.8
1.0
π
c: sit:stand_relatives
y = 0.43x + 0
base+PCs+CG
phenotypic correlation
0.8
0.6
0.4
0.2
0.0
0.06
0.08
0.12
0.15
0.25
0.5
1
π

Supplementary Figure 3. Phenotypic correlation for 32 quantitative and ordered categorical traits in the UK Biobank, as a function of the genomic relationship (π).

Each page shows a panel of 3 plots for the same trait, as indicated in the plot heading. Panel a (top) shows the phenotypic correlation in unrelated pairs, panel b (middle) shows the phenotypic correlation over the entire spectrum of relatedness, and panel c (bottom) shows the phenotypic correlation in close relatives ($\pi > 0.05$).

Different colours in panels a and b indicate the model used to create the residuals from the model fitted to the phenotypes; where the base model (model 1, see methods) includes terms such as sex and genotyping batch, base+CG (model 2) includes base model terms plus birth contemporary group (CG), base+PCs (model 3) indicates base model terms plus 25 principal components from the genomic SNPs, and base+CG+PCs (model 4) indicates based model covariates plus both PC and birth CG. See main methods section for more details on models fitted to the data.

Panel c show the phenotypic correlation for the close-relative genomic relationships using residuals from the model fitting all terms, including PCs and birth CG.

Regression lines shown in each panel are weighted HE regressions in either unrelated ($\pi < 0.02$, panels a and b) or close relative ($\pi > 0.05$, panels b and c) pairs.

Dots (panels a and b) or the height of bars (panel c) show the phenotypic correlation for all data, with error bars indicating 95% Confidence Intervals ($\pm 1.96\ s.e.$, where standard errors are calculated using a block jack-knife approach with 100 blocks of individuals).

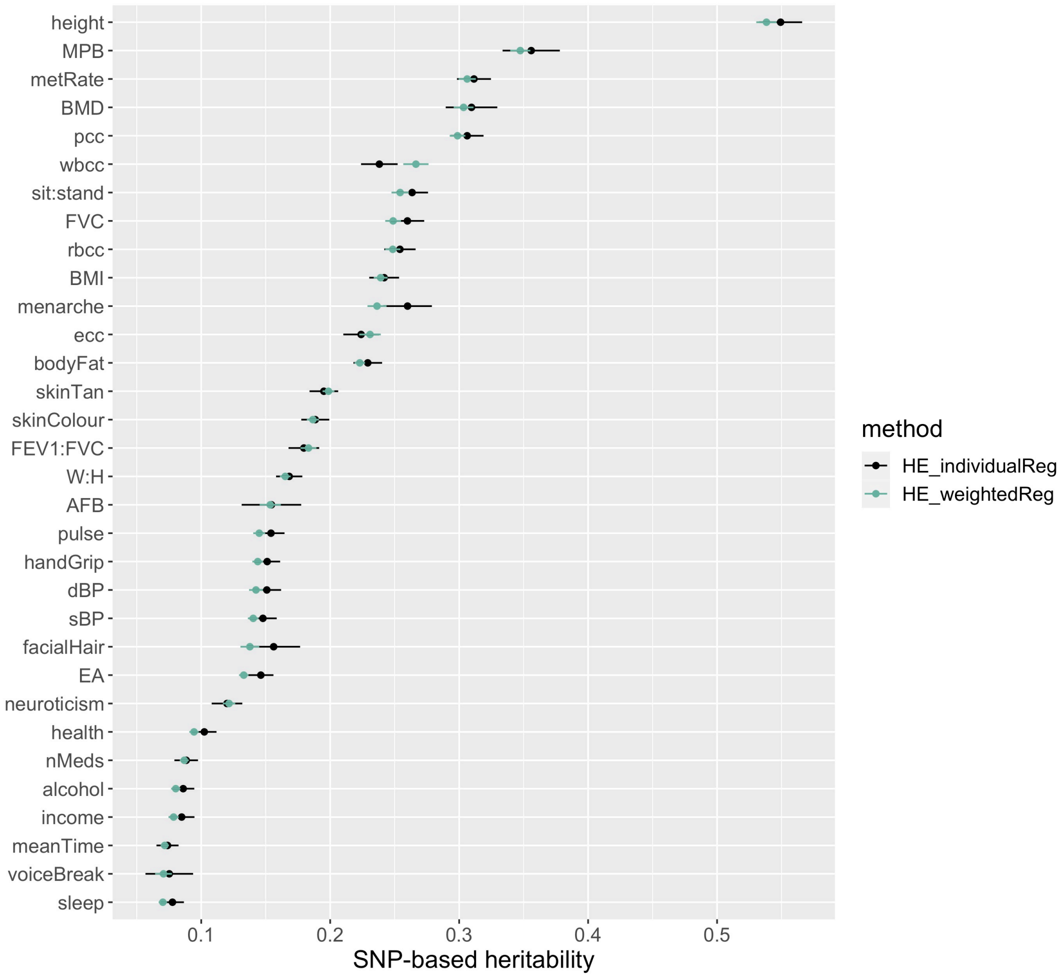

Supplementary Figure 4. The variance explained by common SNP ($\hat{h}^2_{SNP}$) in 32 quantitative and ordered categorical traits in the UK Biobank. Estimates use HE regression with individual level data or a weighted linear regression on the relationship bins. Points indicate the estimate with all data and bars show 95% Confidence Intervals ($\pm 1.96\ s.e.$). Standard errors are either from a blocked jackknife with 100 blocks of individuals (weighted HE regression) or the leave-one-out jackknife approach implemented in GCTA[1] (individual HE regression).

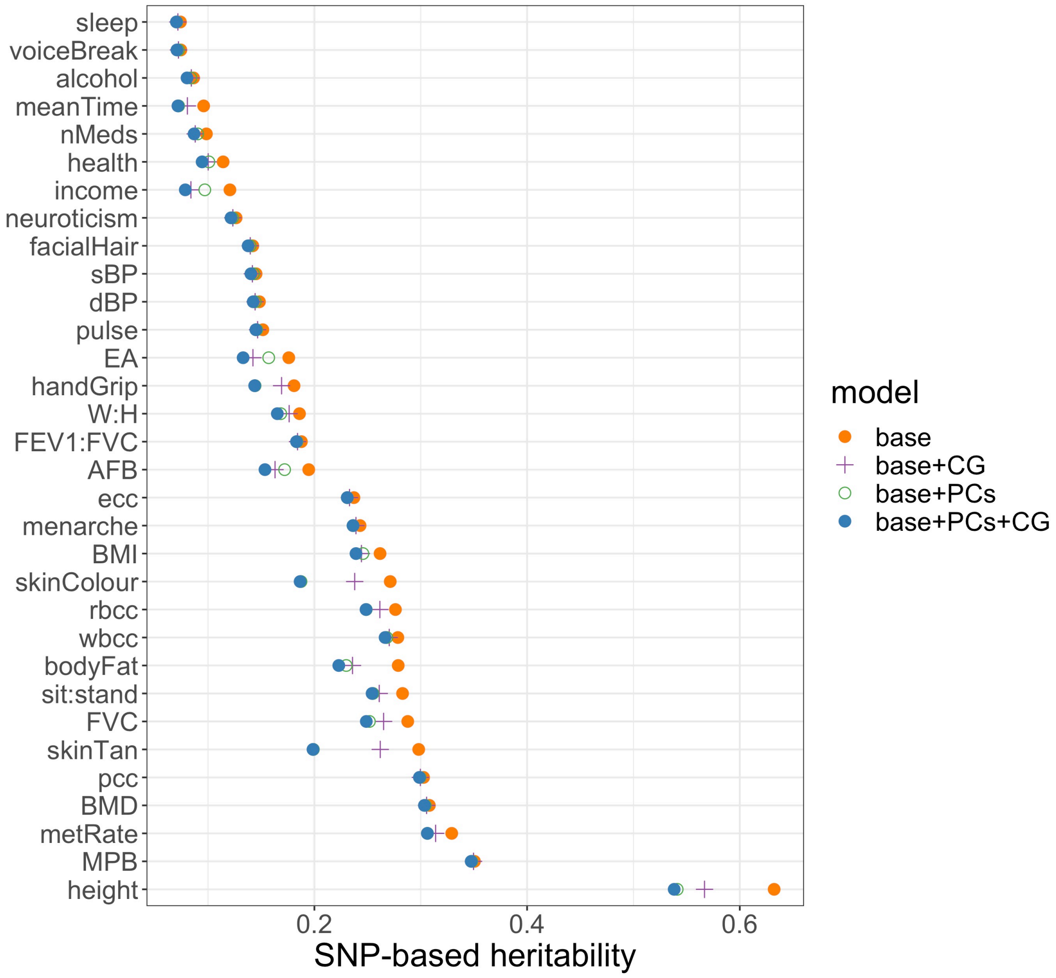

Supplementary Figure 5. The effect of model fitting on the variance explained by common SNP ($\hat{h}^2_{SNP}$) in 32 quantitative and ordered categorical traits in the UK Biobank. Estimation uses a weighted HE regression model. Four different models were fitted to the data prior to the calculation of the SNP-based heritability. See main method section for a full description of the models fitted to the data. Briefly, the models always included terms such as sex and genotyping batch (base), and sometimes included birth contemporary groups (CG) or 25 genomic principal components (PCs), or both CG and PCs.

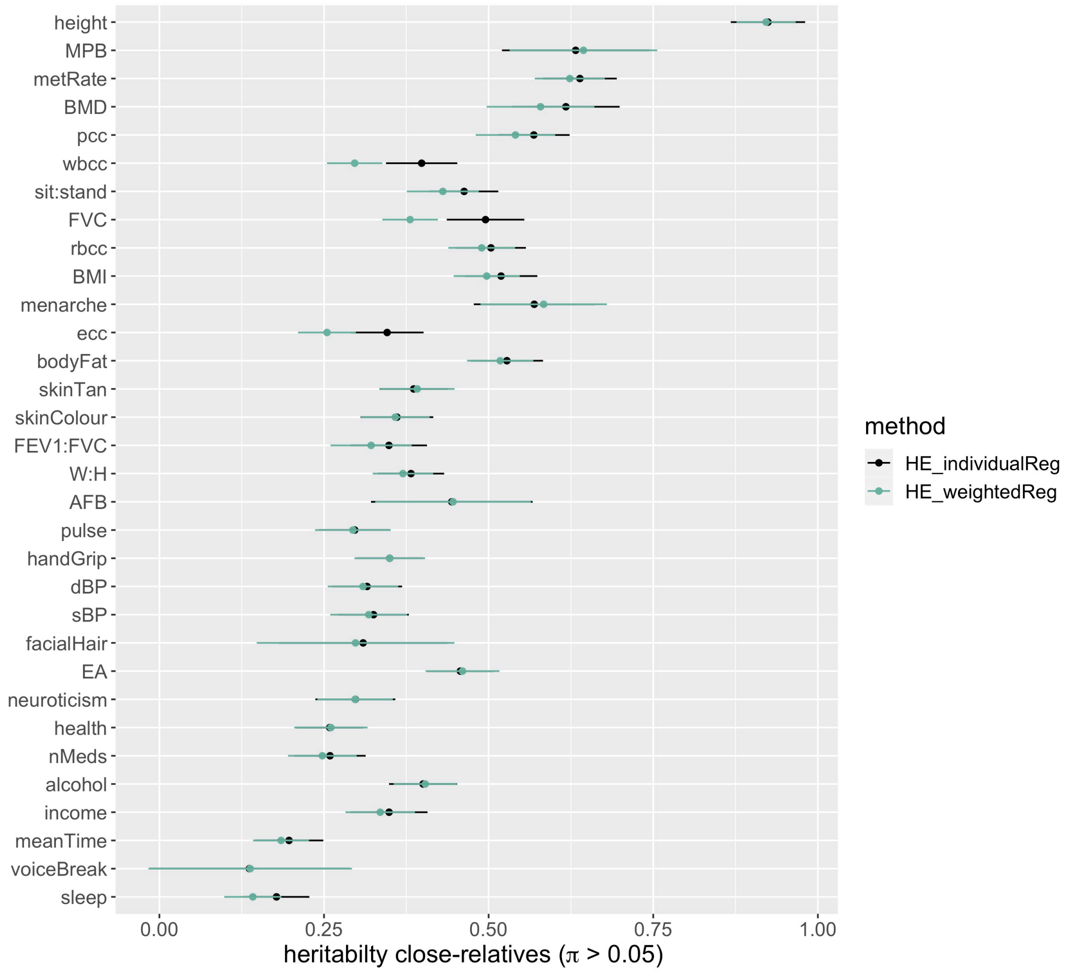

Supplementary Figure 6. Heritability estimated from close relatives ($\hat{h}^2_{\pi>0.05}$) for 32 quantitative and ordered categorical traits in the UK Biobank. Estimation uses either HE regression with individual level data or a weighted linear regression with weights equal to the number of pairs per relationship bin. Bars indicate 95% Confidence Intervals ($\pm 1.96\ s.e.$) and standard errors are from a blocked jackknife approach with 100 blocks of individuals (weighted HE regression) or the leave-one-out jackknife approach implemented in GCTA[1] (individual HE regression).

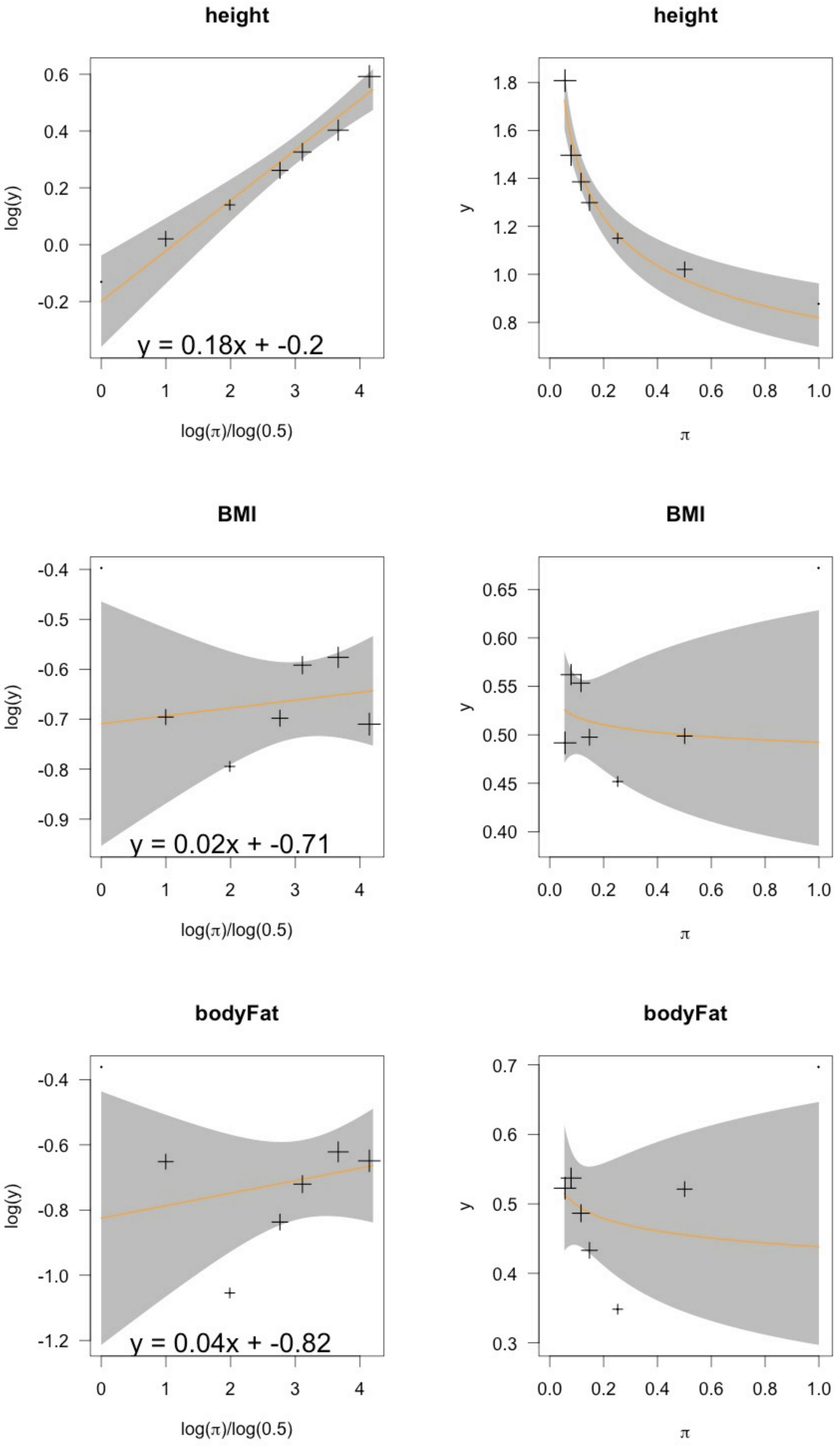
height
log(y)
y = 0.18x + -0.2
log(π)/log(0.5)
height
y
π
BMI
log(y)
y = 0.02x + -0.71
log(π)/log(0.5)
BMI
y
π
bodyFat
log(y)
y = 0.04x + -0.82
log(π)/log(0.5)
bodyFat
y
π

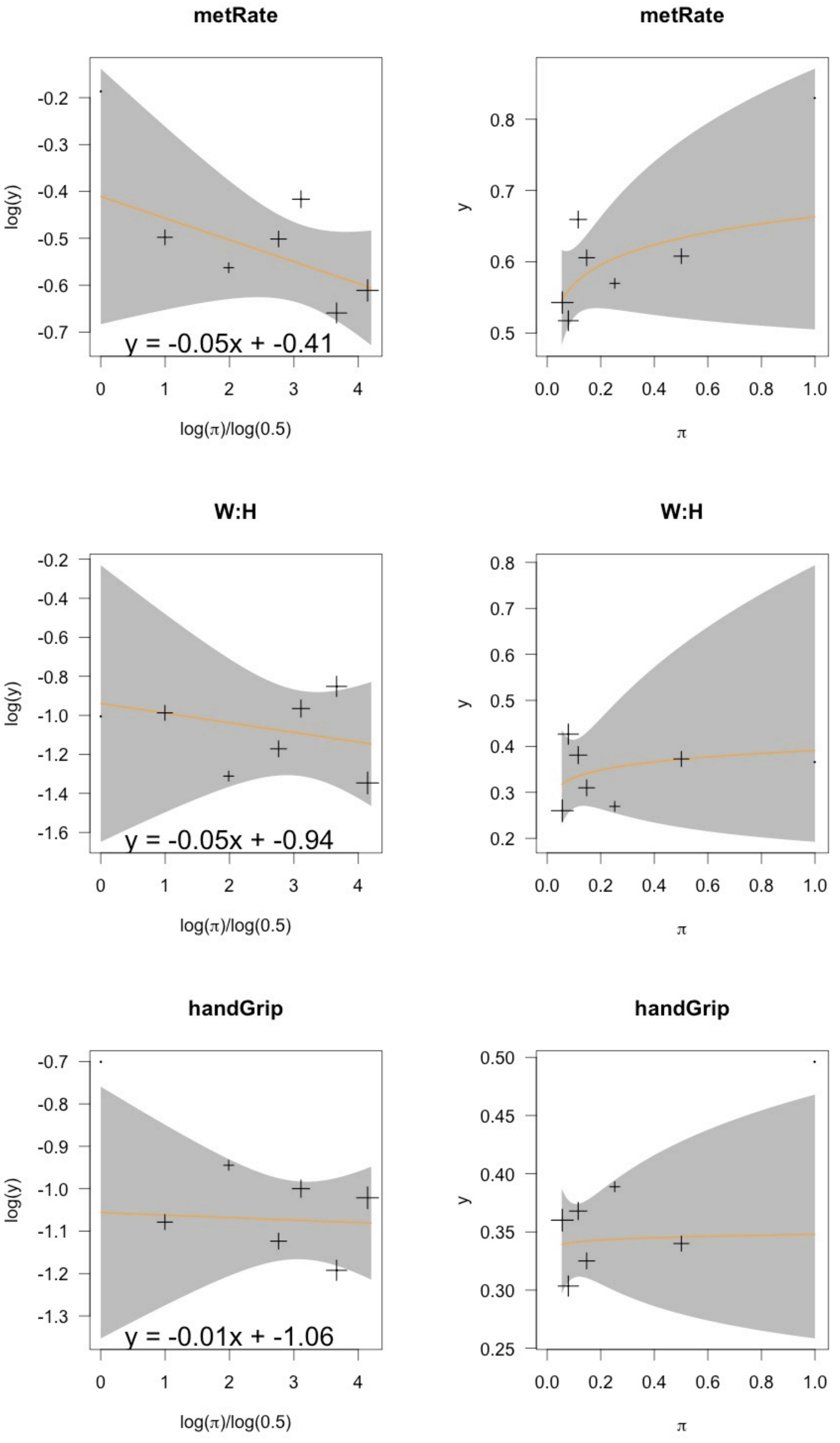
metRate
log(y)
y = -0.05x + -0.41
log(π)/log(0.5)
metRate
y
π
W:H
log(y)
y = -0.05x + -0.94
log(π)/log(0.5)
W:H
y
π
handGrip
log(y)
y = -0.01x + -1.06
log(π)/log(0.5)
handGrip
y
π

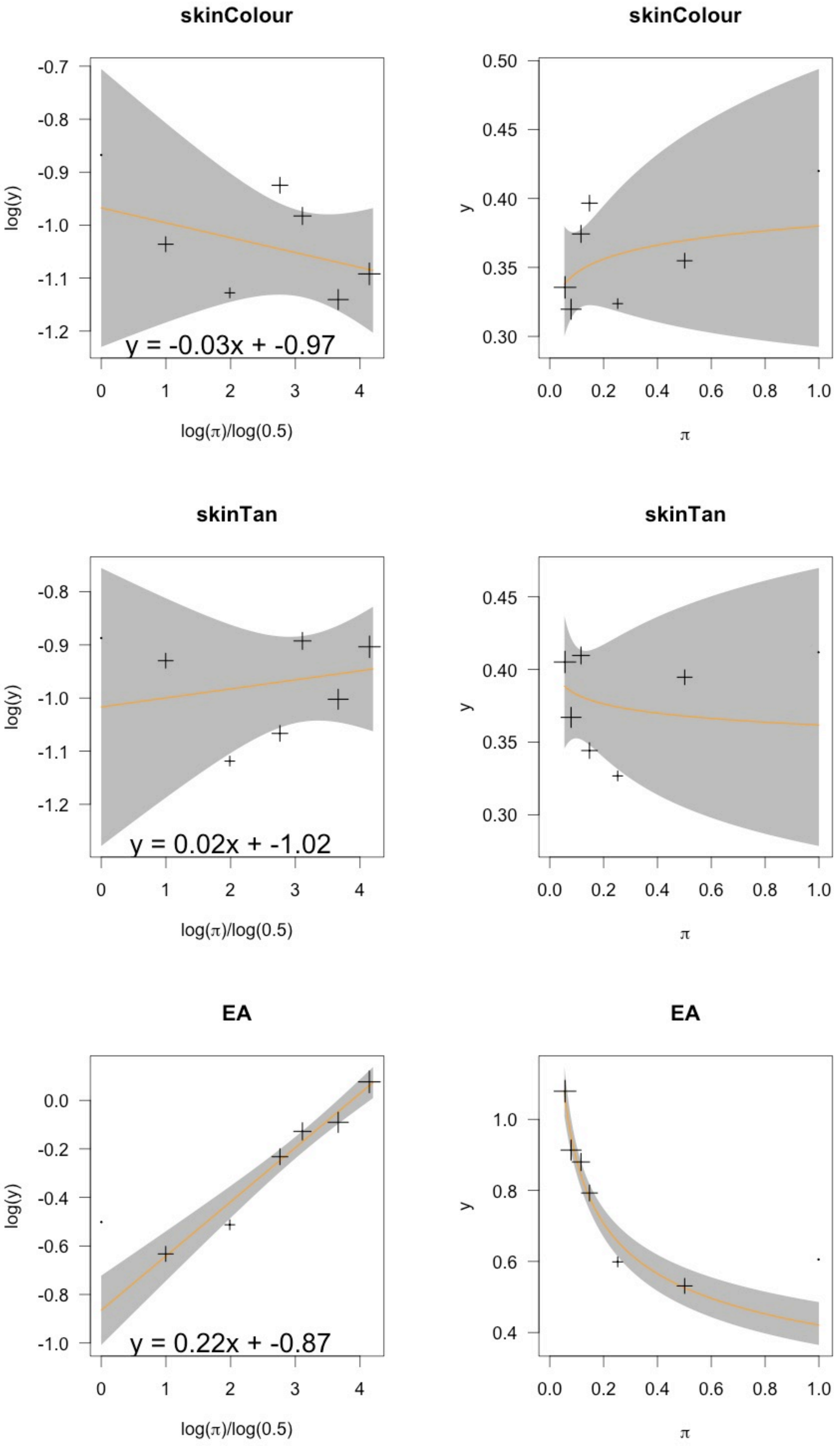
skinColour
log(y)
y = -0.03x + -0.97
log(π)/log(0.5)
skinColour
y
π
skinTan
log(y)
y = 0.02x + -1.02
log(π)/log(0.5)
skinTan
y
π
EA
log(y)
y = 0.22x + -0.87
log(π)/log(0.5)
EA
y
π

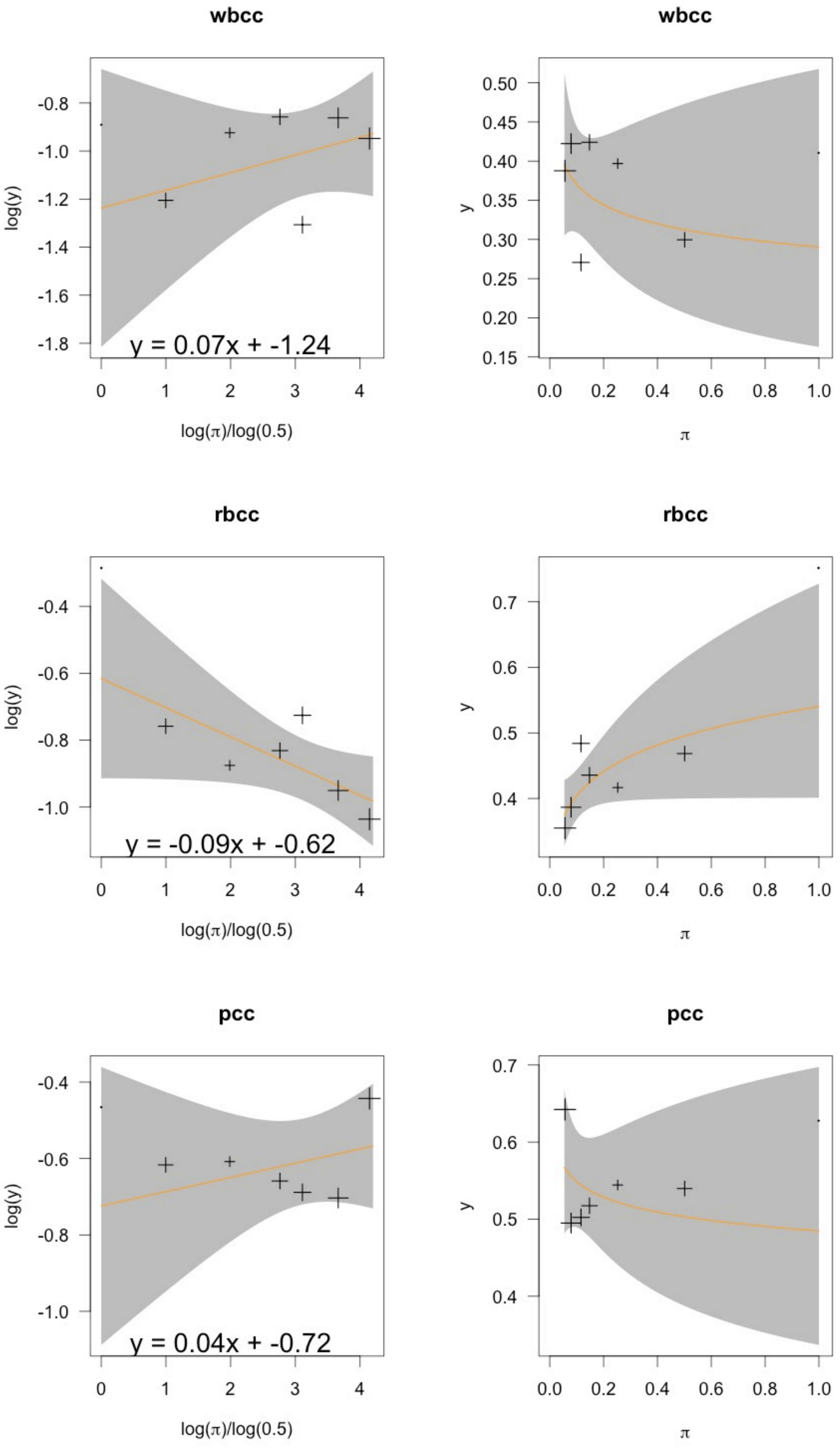
wbcc
log(y)
y = 0.07x + -1.24
log(π)/log(0.5)
wbcc
y
π
rbcc
log(y)
y = -0.09x + -0.62
log(π)/log(0.5)
rbcc
y
π
pcc
log(y)
y = 0.04x + -0.72
log(π)/log(0.5)
pcc
y
π

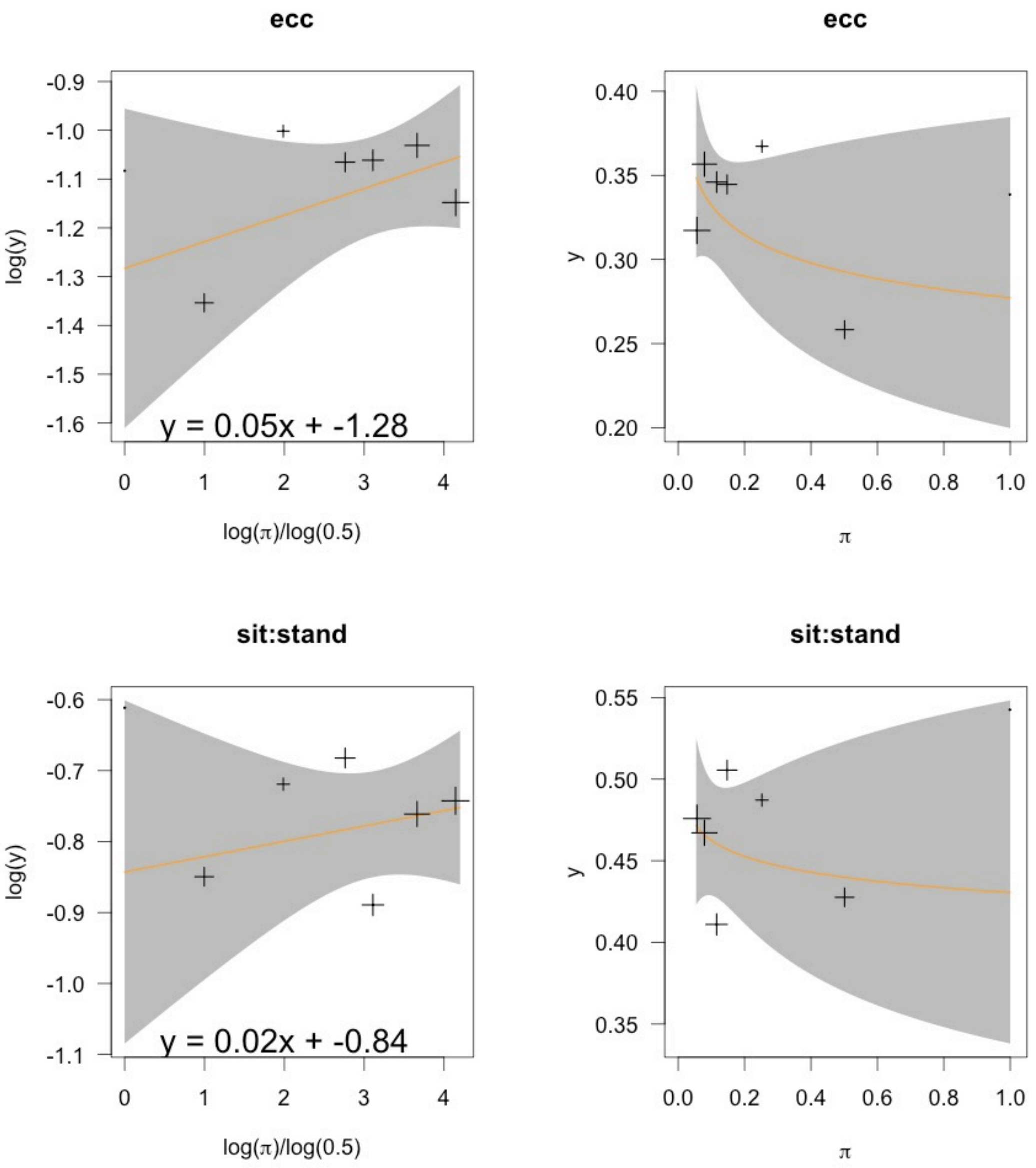

Supplementary Figure 7. Modelling assortative mating in close relatives for 14 traits in the UK Biobank. Each page shows 2-3 traits, as indicated the plot header, on either the log (left) or observed (right) scale. Grey shading indicates the 95% Confidence Interval of the fitted regression, with the best fit regression line shown in orange and the regression equation detailed in the left panel. Points indicate the modelled data, where the size of the point is proportional to the number of pairs contributing to the point.

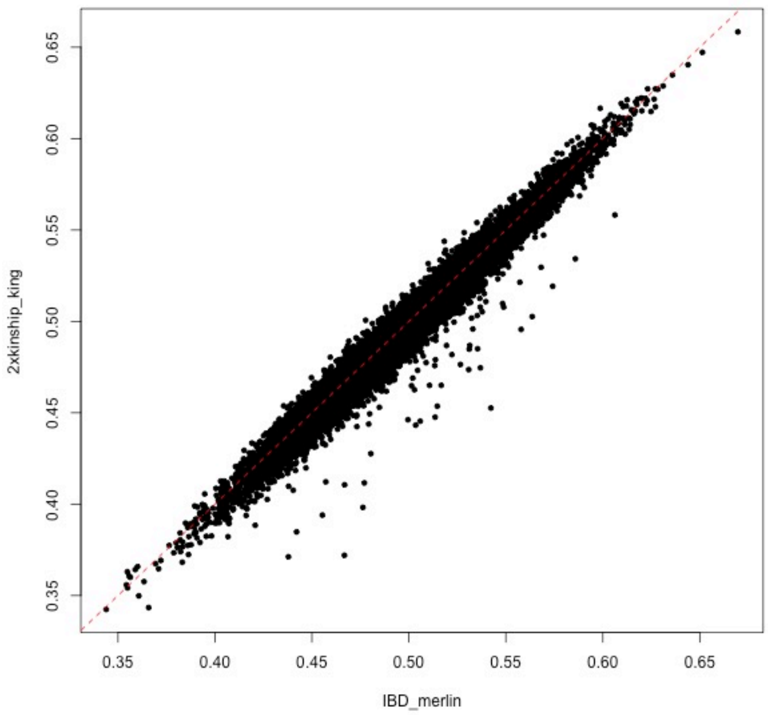

Supplementary Figure 8. Relationship between twice the KING[2,3] kinship coefficient and the proportion of the genome identical-by-descent (IBD) estimated by MERLN[4].

Supplementary Tables

Supplementary Table 1. Trait name (and abbreviation), number of records analysed, UK Biobank field identifiers (f.id) and analysis particulars for 32 quantitative and ordered categorical traits.

Trait	Abbreviation	N	f.eid	Comments
height	height	416,131	f.50.0.0	
pulse	pulse	389,521	f.102.0.0	
sleep duration	sleep	414,505	f.1160.0.0	
diastolic blood pressure	dBP	389,628	f.4079.0.0	
systolic blood pressure	sBP	389,580	f.4080.0.0	
mean time correct match	meanTime	413,307	f.20023.0.0	
body mass index	BMI	415,308	f.21001.0.0	
neuroticism	neuroticism	338,362	f.20127.0.0	
body fat	bodyFat	409,550	f.23099.0.0	
basal metabolic rate	metRate	409,465	f.23105.0.0	
waist:hip ratio	W:H	416,167	f.49.0.0; f.48.0.0	waist/hip
hand grip strength	handGrip	416,124	f.46.0.0; f.47.0.0	maximum
household income	income	358,564	f.738.0.0	5 levels; 1= low.. 5= high
number of medications	nMeds	416,205	f.137.0.0	
skin colour	skinColour	411,315	f.1717.0.0	6 levels; 1= fair.. 6=black
skin tanning	skinTan	408,456	f.1727.0.0	4 levels; 1=very tanned.. 4=never tan
health rating	health	415,476	f.2178.0.0	4 levels; 1=excellent.. 4=poor
years of education2	EA	413,496	f.6138.X.X1	maximum
lung capacity (forced vital capacity)	FVC	379,703	f.3062.0.0	
lung function	FEV1:FVC	378,983	f.3063.0.0; f.3062.0.0	FEV1/FVC
white blood cell count	wbcc	404,211	f.30000.0.0	
red blood cell count	rbcc	404,317	f.30010.0.0	
platelet count	pcc	404,144	f.30080.0.0	
eosinophil count	ecc	402,539	f.30150.0.0	
frequency alcohol intake	alcohol	416,693	f.1558.0.0	6 levels; 1=daily.. 6=never
relative age facial hair[3]	facialHair	186,228	f.2375.0.0	3 levels; 1=younger.. 3=older
relative age voice broke[3]	voiceBreak	178,127	f.2385.0.0	3 levels; 1=younger.. 3=older
male patterned baldness[3]	MPB	191,006	f.2395.0.0	4 levels; 1=no loss.. 4=severe loss
age menarche[3]	menarche	218,158	f.2714.0.0	
age at first birth[3]	AFB	153,682	f.2754.0.0	
bone mineral density	BMD	240,919	f.3148.0.0	
sit to stand ratio	sit:stand	414,868	f.20015.0.0; f.50.0.0	Sitting/standing

[1]where X indicates over all possible values.

[2]calculated according to Okbay[5]

[3]sex-limited trait

Supplementary Table 2. Fields used as fixed effects and covariates with their UKB field identifier (f.eid), and notes giving a brief description of the data.

Fixed effect	f.eid	Notes
genotyping batch	-	106 levels; about 4500 samples per batch
sex	f.31-0.0	2 levels; male and female
Year of birth (YOB)	f.34-0.0	34 levels; 1937 to 1970
Age, age at measurement	f.21003-0.0	31 levels; 40 to 70 years
birth contemporary group (CG)	f.129-0.0; f.130-0.0	378 levels; derived from north and east birth co-ordinates within Great Britain

Supplementary Table 3. Estimates of the slope and intercept (with standard error, s.e.) from individual-level HE regression using unrelated individuals (max. N = 133,387) or weighted regression using all unrelated pairs ($\pi < 0.02$) for the regression of phenotypic covariance on genomic relationship, where the slope of the regression estimates the SNP-based heritability ($\hat{h}^2_{SNP}$).

	Individual-level HE regression				Weighted HE regression			
trait	int.	s.e.	slope	s.e.	int.	s.e.	slope	s.e.
height	-2.07E-05	3.54E-07	0.549	0.009	-3.25E-06	3.36E-08	0.538	0.004
pulse	-1.27E-05	1.91E-07	0.154	0.005	-2.90E-06	2.31E-08	0.145	0.002
sleep	-1.00E-05	1.49E-07	0.078	0.005	-2.52E-06	1.99E-08	0.070	0.002
dBP	-1.26E-05	1.91E-07	0.151	0.006	-2.90E-06	2.38E-08	0.142	0.003
sBP	-1.25E-05	1.90E-07	0.148	0.005	-2.91E-06	2.43E-08	0.140	0.002
meanTime	-9.98E-06	1.56E-07	0.074	0.004	-2.56E-06	2.06E-08	0.072	0.002
BMI	-1.37E-05	2.18E-07	0.242	0.006	-2.76E-06	2.26E-08	0.239	0.003
neuroticism	-1.28E-05	1.79E-07	0.120	0.006	-3.18E-06	2.76E-08	0.122	0.002
bodyFat	-1.36E-05	1.99E-07	0.229	0.006	-2.79E-06	2.18E-08	0.223	0.002
metRate	-1.55E-05	2.51E-07	0.311	0.007	-2.84E-06	2.31E-08	0.306	0.003
W:H	-1.21E-05	1.72E-07	0.168	0.005	-2.68E-06	2.04E-08	0.165	0.002
handGrip	-1.17E-05	1.67E-07	0.151	0.005	-2.62E-06	2.23E-08	0.144	0.002
income	-1.14E-05	1.49E-07	0.085	0.005	-3.10E-06	2.33E-08	0.079	0.002
nMeds	-1.03E-05	1.65E-07	0.088	0.005	-2.58E-06	1.86E-08	0.087	0.002
skinColour	-1.26E-05	2.06E-07	0.188	0.006	-2.69E-06	2.15E-08	0.187	0.002
skinTan	-1.28E-05	1.75E-07	0.195	0.006	-2.72E-06	2.30E-08	0.199	0.002
health	-1.06E-05	1.49E-07	0.102	0.005	-2.65E-06	1.73E-08	0.094	0.002
EA	-1.16E-05	1.46E-07	0.146	0.005	-2.91E-06	2.53E-08	0.133	0.002
FVC	-1.49E-05	2.40E-07	0.260	0.007	-3.04E-06	2.46E-08	0.249	0.003
FEV1:FVC	-1.31E-05	2.13E-07	0.180	0.006	-2.86E-06	2.48E-08	0.183	0.003
wbcc	-1.39E-05	2.41E-07	0.238	0.007	-2.84E-06	2.84E-08	0.266	0.005
rbcc	-1.43E-05	2.20E-07	0.254	0.006	-2.79E-06	2.24E-08	0.249	0.003
pcc	-1.54E-05	2.47E-07	0.306	0.006	-2.87E-06	2.11E-08	0.299	0.003
ecc	-1.36E-05	2.53E-07	0.224	0.007	-2.75E-06	2.34E-08	0.231	0.004
alcohol	-1.02E-05	1.28E-07	0.086	0.004	-2.63E-06	1.62E-08	0.080	0.002
facialHair	-2.20E-05	4.53E-07	0.156	0.010	-5.65E-06	5.06E-08	0.138	0.004
voiceBreak	-2.10E-05	5.73E-07	0.075	0.009	-5.84E-06	5.53E-08	0.071	0.003
MPB	-2.61E-05	4.11E-07	0.356	0.011	-5.80E-06	5.46E-08	0.347	0.004
menarche	-2.17E-05	3.62E-07	0.260	0.010	-4.94E-06	3.86E-08	0.236	0.004
AFB	-2.64E-05	4.68E-07	0.154	0.012	-7.20E-06	5.38E-08	0.154	0.004
BMD	-2.18E-05	4.02E-07	0.310	0.010	-4.91E-06	4.04E-08	0.303	0.004
sit:stand	-1.43E-05	2.28E-07	0.264	0.006	-2.75E-06	2.45E-08	0.254	0.003

Supplementary Table 4. Estimates of the slope and intercept (with standard error, s.e.) from individual-level HE regression using relatives individuals (max. N ~ 150K) or weighted regression using close relative pairs ($\pi > 0.05$) for the regression of phenotypic covariance on genomic relationship, where the slope of the regression estimates the heritability in close-relative ($\hat{h}^2_{\pi>0.05}$).

	Individual-level HE regression				Weighted HE regression			
trait	int.	s.e.	slope	s.e.	int.	s.e.	slope	s.e.
height	0.050	0.006	0.924	0.029	0.050	0.006	0.924	0.029
pulse	-0.006	0.006	0.297	0.027	-0.006	0.006	0.297	0.027
sleep	-0.005	0.006	0.178	0.025	-0.005	0.006	0.178	0.025
dBP	-0.010	0.006	0.315	0.027	-0.010	0.006	0.315	0.027
sBP	-0.010	0.006	0.325	0.027	-0.010	0.006	0.325	0.027
meanTime	-0.006	0.006	0.197	0.027	-0.006	0.006	0.197	0.027
BMI	-0.001	0.006	0.519	0.028	-0.001	0.006	0.519	0.028
neuroticism	-0.005	0.007	0.298	0.031	-0.005	0.007	0.298	0.031
bodyFat	-0.005	0.006	0.528	0.028	-0.005	0.006	0.528	0.028
metRate	-0.006	0.006	0.639	0.029	-0.006	0.006	0.639	0.029
W:H	-0.004	0.005	0.382	0.026	-0.004	0.005	0.382	0.026
handGrip	-0.001	0.005	0.350	0.026	-0.001	0.005	0.350	0.026
income	0.019	0.006	0.349	0.030	0.019	0.006	0.349	0.030
nMeds	-0.002	0.006	0.259	0.028	-0.002	0.006	0.259	0.028
skinColour	-0.001	0.006	0.361	0.028	-0.001	0.006	0.361	0.028
skinTan	-0.003	0.006	0.386	0.026	-0.003	0.006	0.386	0.026
health	0.011	0.006	0.259	0.026	0.011	0.006	0.259	0.026
EA	0.034	0.006	0.457	0.026	0.034	0.006	0.457	0.026
FVC	0.016	0.006	0.495	0.030	0.016	0.006	0.495	0.030
FEV1:FVC	-0.011	0.006	0.349	0.029	-0.011	0.006	0.349	0.029
wbcc	-0.002	0.006	0.398	0.028	-0.002	0.006	0.398	0.028
rbcc	-0.009	0.006	0.504	0.027	-0.009	0.006	0.504	0.027
pcc	-0.004	0.006	0.569	0.028	-0.004	0.006	0.569	0.028
ecc	-0.001	0.006	0.346	0.028	-0.001	0.006	0.346	0.028
alcohol	0.000	0.005	0.401	0.026	0.000	0.005	0.401	0.026
facialHair	-0.012	0.012	0.309	0.065	-0.012	0.012	0.309	0.065
voiceBreak	0.015	0.013	0.137	0.077	0.015	0.013	0.137	0.077
MPB	-0.006	0.012	0.632	0.057	-0.006	0.012	0.632	0.057
menarche	-0.022	0.010	0.569	0.047	-0.022	0.010	0.569	0.047
AFB	0.028	0.014	0.444	0.063	0.028	0.014	0.444	0.063
BMD	-0.024	0.008	0.617	0.042	-0.024	0.008	0.617	0.042
sit:stand	0.000	0.006	0.463	0.026	0.000	0.006	0.463	0.026

Supplementary Table 5. Estimates of the intercept (a) and slope (b) when modelling assortative mating, and their corresponding transformed values of the equilibrium heritability ($\hat{h}^2_{EQ}$) and correlation between mates ($\hat{r}$). Standard errors (s.e.) for all estimates are shown.

	a	s.e.	b	s.e.	$\hat{h}^2_{EQ}$ [1]	s.e.	$\hat{r}$ [2]	s.e.
height	-0.199	0.043	0.178	0.018	0.819	0.035	0.237	0.036
BMI	-0.709	0.147	0.016	0.065	0.492	0.072	0.032	0.139
bodyfat	-0.825	0.150	0.038	0.062	0.438	0.066	0.090	0.161
metRate	-0.411	0.168	-0.046	0.079	0.663	0.112	-0.068	0.102
W:H	-0.939	0.325	-0.050	0.143	0.391	0.128	-0.122	0.305
handGrip	-1.056	0.222	-0.006	0.098	0.348	0.078	-0.017	0.274
skinColour	-0.967	0.217	-0.028	0.103	0.380	0.082	-0.073	0.248
skinTan	-1.016	0.243	0.017	0.109	0.362	0.088	0.047	0.316
EA	-0.866	0.103	0.224	0.045	0.421	0.043	0.596	0.192
wbcc	-1.236	0.177	0.073	0.088	0.291	0.052	0.262	0.369
rbcc	-0.615	0.208	-0.088	0.097	0.541	0.112	-0.155	0.134
pcc	-0.724	0.146	0.037	0.064	0.485	0.071	0.078	0.146
ecc	-1.283	0.274	0.054	0.123	0.277	0.076	0.203	0.518
sit:stand	-0.843	0.175	0.022	0.076	0.431	0.075	0.051	0.190

[1]estimated as exp(a), see methods section in main text.
[2]estimates as [exp(b)-1]/exp(a), see methods in main text.

	$\sigma^2_{a(RM)}$	s.e.	$\sigma^2_{P(RM)}$	s.e.	$\hat{h}^2_{RM}$	s.e.	$\sigma^2_{a(EQ)}/\sigma^2_{a(RM)}$ [3]	s.e.	$\hat{h}^2_{EQ}/\hat{h}^2_{RM}$ [4]	s.e.
height	0.660	0.045	0.841	0.012	0.785	0.012	1.241	0.034	1.044	0.014
BMI	0.484	0.102	0.992	0.032	0.488	0.032	1.016	0.068	1.008	0.036
bodyfat	0.421	0.090	0.983	0.026	0.428	0.026	1.041	0.070	1.023	0.042
metRate	0.694	0.165	1.030	0.055	0.673	0.055	0.957	0.069	0.985	0.018
W:H	0.410	0.187	1.019	0.060	0.403	0.060	0.954	0.123	0.972	0.069
handGrip	0.350	0.110	1.002	0.034	0.349	0.034	0.994	0.096	0.996	0.062
skinColour	0.391	0.121	1.011	0.040	0.387	0.040	0.973	0.094	0.983	0.056
skinTan	0.356	0.125	0.994	0.039	0.358	0.039	1.017	0.114	1.011	0.074
EA	0.315	0.055	0.895	0.014	0.353	0.014	1.334	0.099	1.194	0.071
wbcc	0.269	0.074	0.978	0.024	0.275	0.024	1.082	0.111	1.058	0.083
rbcc	0.586	0.169	1.045	0.057	0.561	0.057	0.923	0.076	0.965	0.027
pcc	0.467	0.099	0.982	0.030	0.475	0.030	1.039	0.071	1.020	0.039
ecc	0.262	0.107	0.985	0.032	0.266	0.032	1.059	0.145	1.043	0.109
sit:stand	0.421	0.106	0.991	0.032	0.425	0.032	1.022	0.082	1.013	0.048

[3]inflation in the (random-mating) genetic variance due to assortative mating.
[4]inflation in the (random-mating) heritability due to assortative mating.

Supplementary Notes

SUPPLEMENTARY NOTE 1:
Effect of incomplete linkage disequilibrium on phenotypic covariance

Simulation was used to investigate the effect of incomplete linkage disequilibrium between causal variants and common variants included in the genomic relationship matrix (GRM) on the phenotypic covariance across the relatedness spectrum. Specifically, we wanted to investigate if the observed increased phenotypic covariance in distant relative pairs ($0.02 < \pi < 0.05$) could be recapitulated through incomplete linkage disequilibrium by selecting causal variants with lower minor allele frequency than common (HapMap3) variants used to construct the GRM.

1.1 Method overview
Phenotypes were simulated under a simple A + E model (i.e. phenotype = additive genetic effect + environmental deviation) for the 417,060 individuals from the UK Biobank included in our study. For each replicate, we calculated the average covariance between pairs of individuals in each of the 54 relationship bins from the GRM. Simulations used the GRM generated and described in the main text. We conducted 100 replicate simulations of four scenarios; where scenarios differed in the causal variant distribution for allele frequency and imputation accuracy. The final scenario was when causal variants were sampled from genotyped SNPs. The simulated heritability of the trait was 0.8.

1.2 Selection of causal variants
For each replicate, we selected 10,000 causal variants from a pool of variants defined by four scenarios. Scenarios 1, 3 and 4 used imputed data. Briefly, all UK Biobank genotypes were cleaned and imputed to the Haplotype Reference Consortium (HRC)[6] and UK10K[7] reference panels by Bycroft et al.[3]. Genotype probabilities were converted to hard-call genotypes using PLINK2[8] (--hard-call 0.1) for bi-allelic variants with info score > 0.3. Basic QC applied to imputed variants including a Hardy-Weinberg equilibrium test p-value > $1x10^{-5}$ and minor allele count > 100 (as assessed in the unrelated European sample). The first scenario selected causal variants from the ~1.1M HapMap3 variants used to create the GRM. As described in the main text, all these variants had minor allele frequency (MAF) > 0.01. The second scenario selected causal variants from the 558,730 autosomal genotyped variants supplied by the UK Biobank[3] that passed QC filters with MAF > 0.01. QC filters applied to the genotyped variants included a Hardy-Weinberg equilibrium test p-value > $1x10^{-5}$, missing genotype rate < 0.05 and minor allele count > 100 (as assessed in the unrelated European sample). The third scenario selected causal variance from imputed variants passing basic QC filters, while the final scenario applied an additional QC filter to imputed variants passing basic QC to include only those variants with high imputation accuracy (info score > 0.95). A summary of the scenarios used to select causal variants is provide in Supplementary Table 6.

Supplementary Table 6. A summary of simulation scenarios used to select causal variants.

Scenario	N variants	MAF	comment
1	1,123,347	> 0.01	Imputed HapMap3 SNP used to construct GRM
2	558,730	> 0.01	Common genotyped variants passing basic QC
3	23,223,051	> 0.0002	Imputed variants passing basic QC
4	9,430,259	> 0.0002	Imputed variants passing basic QC with high imputation accuracy (info score > 0.95).

1.3 Simulation of phenotypes

Phenotypes (P) were simulated using GCTA (v1.9)[9] as the sum of genetic effects (A) and environmental deviations (E) for each individual. For each simulation, allele substitution effects were generated for the 10,000 causal variants from $N(0,\sigma^2_a/[2p_j(1-p_j)M])$, where M is the number of causal loci (M = 10,000), p_j is the minor allele frequency of the j-th causal variant and σ^2_a is the additive genetic variance (σ^2_a = 0.8). Additive genetic effects were then the sum of simulated effect for each locus, dependent on the genotype of the individual. Environmental deviations were generated from $N(0,\sigma^2_e)$, where σ^2_e is the environmental variance (σ^2_e = 0.2). Thus, the expectation for the heritability (h^2) of the simulated trait is $h^2 = \sigma^2_a/(\sigma^2_a+\sigma^2_e) = 0.8$.

1.4 Parameter estimates

Heritability was estimated using a weighted linear regression of the average phenotypic covariance on the mid-point of the genomic relationship bins in either unrelated ($\pi < 0.02$) or relative ($\pi > 0.05$) pairs.

1.5 Results - distribution of MAF and imputation quality

The minor allele frequency (MAF) and imputation quality distribution for causal variants differed markedly across the scenarios. All HapMap3 variants used to construct the GRM (scenario 1) were common (MAF > 0.01) and almost all variants had high imputation quality (info score > 0.95, Supplementary Figure 9). Genotyped variants (scenario 2) were also all common (MAF > 0.01) but had more than twice the number of low frequency variants (0.01 < MAF < 0.1) compared to the HM3 variants. In contrast, less than half the imputed variants passing basic QC filters (scenario 3) were common with imputation quality generally being low-moderate for most of these variants. The final scenario (scenario 4), where imputed variants were selected to have high imputation accuracy, resulted in about 15% of variants being rare and is somewhat intermediate between scenarios 1 and 3 in terms of MAF frequency distribution and imputation accuracy. Note that although genotyped SNP in scenario 2 were not imputed, up to 5% missing values (per variant) were tolerated in this scenario.

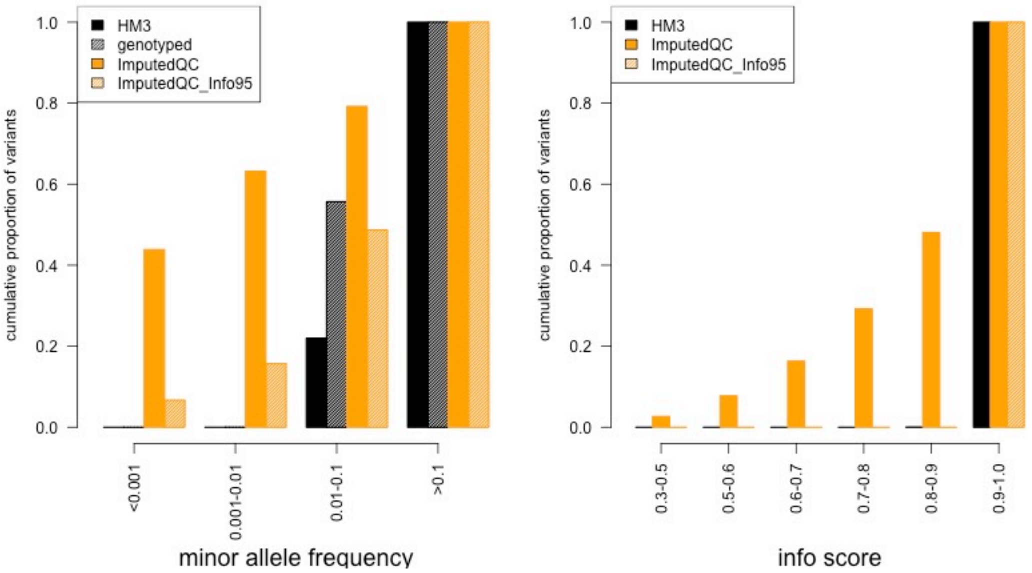

Supplementary Figure 9. The minor allele frequency distribution (left) and imputation accuracy (right) for 10,000 randomly selected causal variants in four scenarios: (i) imputed HapMap3 variants, (ii) common genotyped SNP, (iii) all imputed QC'd variants and (iv) imputed QC'd variants with high imputation accuracy (info score > 0.95).

1.6 Results - parameter estimates

The heritability estimates were not significantly different from the simulated value in unrelated (π < 0.02) and relative (π > 0.05) pairs when causal variants were selected from the HapMap3 variants used to construct the GRM (scenario 1, χ_1^2 = 3.8, p = 0.05; Supplementary Table S7). This implies little loss in information between causal variants and the relationships estimated in the GRM. Thus, the phenotypic covariance is directly proportion to $\pi\hat{h}^2_{SNP}$ for any pair of individuals under this scenario.

In contrast, the phenotypic covariance showed two distinct linear sections when the causal variants were not included in the GRM (scenarios 2-4). There was a smooth transition between the two linear sections in distantly related individuals (0.02 < π < 0.05, Supplementary Figure 10). Although the slope in relatives (π > 0.05) and intercepts were in accordance with the expected values, the slope in unrelated individuals was significantly lower than the expected value of 0.8 in all cases (scenario 2: slope = 0.442 s.e. 0.005; scenario 3: slope = 0.259 s.e. 0.001; scenario 4: slope = 0.610 s.e. 0.002). A lower slope indicates that the covariance between unrelated pairs is lower than that expected from their estimated genomic relationship. This could be an artefact of technical errors (i.e. poor imputation quality or missing genotypes) but the lower than expected covariance in unrelated individuals is consistently observed across all our simulations of high and low imputation accuracy, and genotype variants (scenarios 2-4).

Supplementary Table 7. Intercept and slope (standard error) from the regression of phenotypic covariance on genomic relationships (π) for 100 replicate simulations, where causal variants were selected under 4 scenarios.

Scenario	pairs	intercept	slope
1: HapMap3	unrelated (π < 0.02)	< 0.001 (< 0.001)	0.789 (0.003)
	relatives (π > 0.05)	< 0.001 (< 0.001)	0.796 (0.002)
2: Genotyped SNP	unrelated (π < 0.02)	< 0.001 (< 0.001)	0.442 (0.005)
	relatives (π > 0.05)	- 0.001 (< 0.001)	0.793 (0.002)
3: imputedQC	unrelated (π < 0.02)	< 0.001 (< 0.001)	0.259 (0.001)
	relatives (π > 0.05)	- 0.002 (< 0.001)	0.781 (0.002)
4: imputedQC_info95	unrelated (π < 0.02)	< 0.001 (< 0.001)	0.610 (0.002)
	relatives (π > 0.05)	- 0.001 (< 0.001)	0.798 (0.002)

We propose incomplete linkage disequilibrium (LD) between causal variants and the HapMap3 variants used to construct the GRM are the driving the lower covariance in unrelated individuals. Incomplete LD arises when the MAF for causal variants differs from MAF of variants used to construct the GRM. This includes when causal variants have lower frequency than the HM3 variants used to construct the GRM (i.e. MAF < 0.01), but also when the distribution of MAF for causal variants differs from the HM3 variants. For example, consider the genotyped variants from scenario 2. All variants were common (MAF > 0.01), similar to the HM3 variants, but the genotyped variants had a greater proportion of low frequency variants (0.01 < MAF < 0.1). Thus, the average minor allele frequency of genotyped variants (0.142) is lower than the average minor allele frequency of HM3 variants (0.237). Wray[10] shows that $r_{max}^2 = \frac{p_A(1-p_B)}{1-p_A p_B}$, where r_{max}^2 is a measure of LD and the maximum squared correlation between two loci, p_A and p_B are the allele frequencies at loci *A* and *B* respectively, and we assign alleles such that $p_A < p_B$. This implies, on average, causal variants would expect a loss of about half the simulated heritability in unrelated individuals due to incomplete LD (i.e. $r_{max}^2 = 0.531$). Yang et al.[11] provide a more detailed explanation of the expected loss in information due to LD. In Supplementary Note 1 they show that $\hat{h}_{SNP}^2 = \frac{\overline{r_{QM}^2}}{\overline{r_{MM}^2}} h^2$, where $\hat{h}_{SNP}^2$ is the SNP-based heritability or the slope in unrelated individuals, $\overline{r_{QM}^2}$ and $\overline{r_{MM}^2}$ are the average LD r^2 between causal variants and markers ($\overline{r_{QM}^2}$), or among markers used to construct the GRM ($\overline{r_{MM}^2}$), respectively, and h^2 is the simulated trait heritability. We observed a slope of 0.442 (s.e. 0.005), which is of a similar magnitude to our crude approximation, and suggest that incomplete LD is a key factor determining the two distinct linear sections of phenotypic covariance in relative (π > 0.05) and unrelated (π < 0.02) pairs.

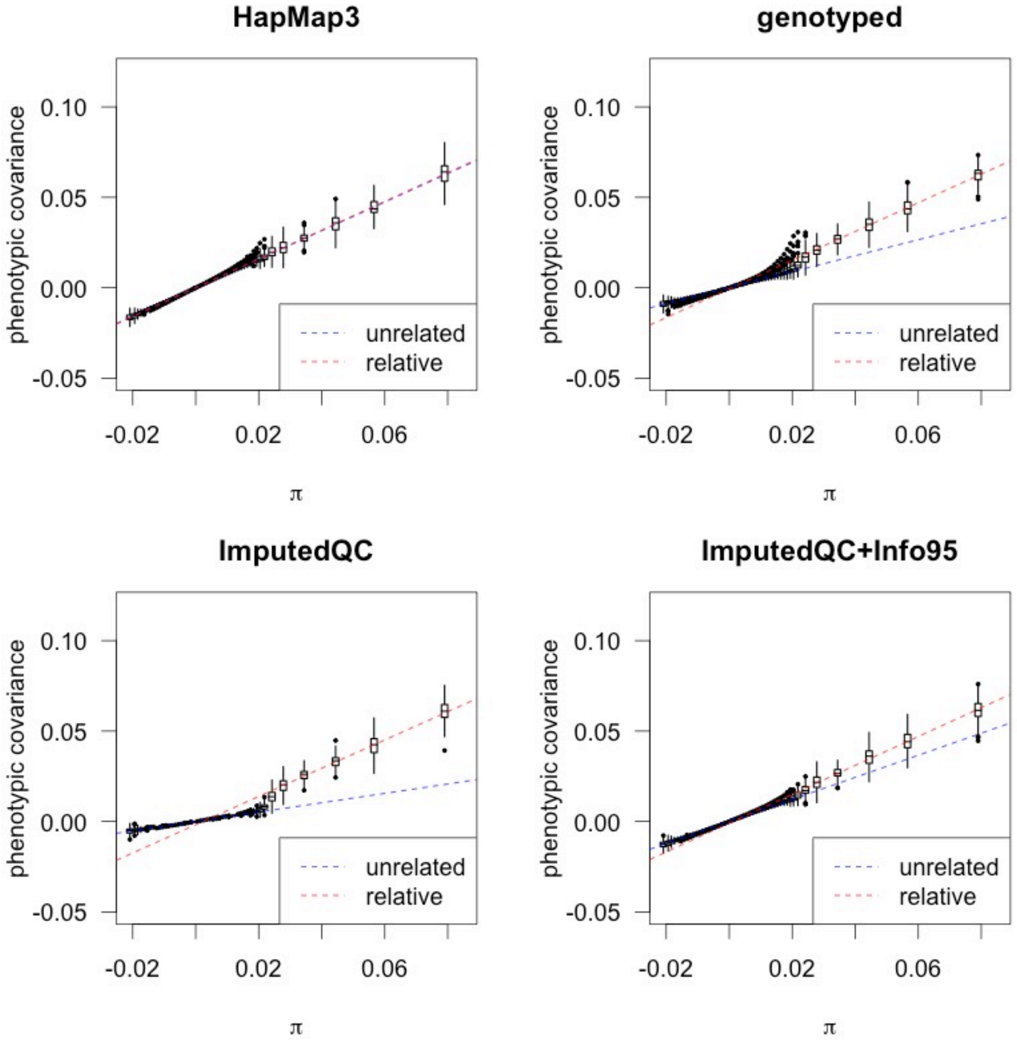

Supplementary Figure 10. Phenotypic covariance for 100 replicate simulations, where the simulated heritability is 0.8 and four different scenarios were used to select causal variants. Shown in red and blue are the regression lines through unrelated ($\pi < 0.02$) or relative ($\pi > 0.05$) pairs. Boxes represent the median and interquartile range (IQR = Q3 - Q1), and whiskers are Q3$\pm$1.5*IQR from 100 replicate simulations. Points indicate outlier replicates.

SUPPLEMENTARY NOTE 2:
Phenotypic covariance due to epistatic interactions

The presence of epistasis (i.e. non-additive interaction of alleles at different loci) could increase the phenotypic covariance between pairs, particularly in close relatives. This note (i) uses simulation to describe the effect of epistatic variance on the phenotypic covariance in relatives, (ii) tests 14 phenotypes in the UK Biobank for epistatic variance, and (iii) determines the power of our test to detect epistasis. The expectation, following[12], is that the phenotypic covariance will increase proportional to square of the genomic relationship (π^2).

2.1.1 Simulation Overview

We simulated phenotypes as the sum of genetic (G) and environmental (E) effects using real genotypes from our sample of 417,060 individuals from the UK Biobank. Simulated genetic effects were solely due to epistatic (or interacting) loci. There were 100 replicate simulations where each replicate selected a different set of 5,000 pairs of unlinked loci (i.e. 10,000 causal variants in total) from the 1,123,347 HapMap3 variants used to create the GRM. We calculated the phenotypic covariance between pairs of individuals in the 54 relationship bins from the GRM described in the main text. The broad-sense heritably (H^2) of the simulated trait was 0.8.

2.1.2 Simulated phenotypes

Phenotypes (P) were simulated as the sum of genetic effects (G) and environmental deviations (E) for each individual. Genetic effects are the sum of simulated epistatic effects, dependent on the (observed) genotype for an individual. Epistatic effects are generated from a unit normal distribution and $G_j = [\boldsymbol{X1_j} \circ \boldsymbol{X2_j}]\boldsymbol{u}$, where G_j is the genetic effect of individual *j*, $\boldsymbol{X1_j}$ and $\boldsymbol{X2_j}$ are the standardised genotypes for individual j at the first and second locus respectively of an interacting pair, and $\boldsymbol{u}$ is the epistatic effect of the corresponding pair of loci. In other words, if we considered two biallelic loci (A/a and B/b), then the genetic effects are:

	**BB**	**Bb**	**bb**
**AA**	0	0	0
**Aa**	0	u	2u
**aa**	0	2*u*	4*u*

Genetic effects for all individuals (G) were scaled to be N(0,1) and environmental deviations sampled from a normal distribution with mean 0 and variance $(1 - H^2)/H^2$.

2.1.3 Simulation Results

The covariance was approximately zero between unrelated individuals (Supplementary Figure 11, left panel). This is consistent with simulation conditions where epistatic variance is not generated between unlinked loci. For relatives, phenotypic covariance increased in relationship bins in proportion to the expected value of $\pi^2 H^2$ (Supplementary Figure 11, right panel).

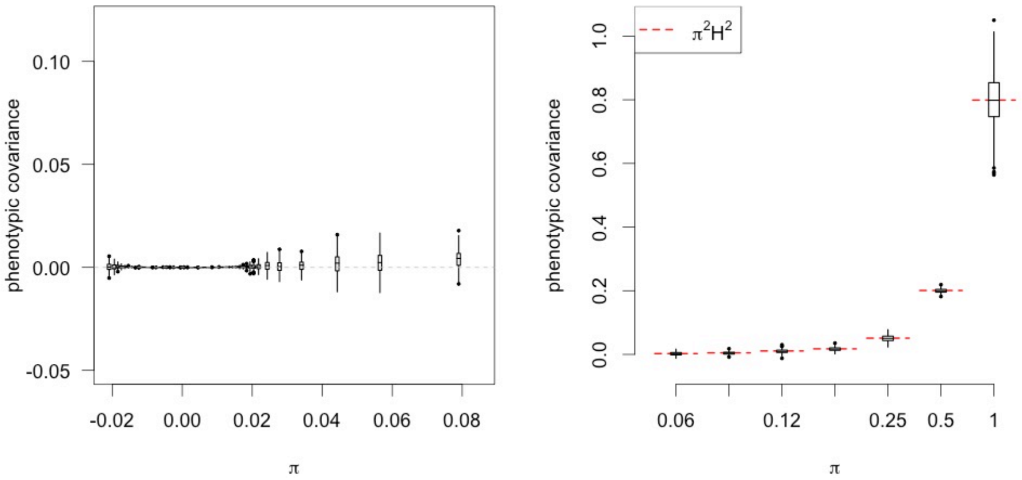

Supplementary Figure 11. Phenotypic covariance in un- and distantly related ($\pi < 0.08$, left) or relative ($\pi > 0.05$, right) pairs for 100 replicate simulations, where the simulated (broad-sense) heritability is 0.8. Boxes represent the median and interquartile range (IQR = Q3 - Q1), and whiskers are Q3$\pm$1.5*IQR from 100 replicate simulations. Points indicate outlier replicates. Red horizontal lines are the expected covariance ($\pi^2 H^2$).

2.2 Testing UK Biobank phenotypes for epistasis

We tested the subset of 14 phenotypes in the UK Biobank with moderate-high SNP-based heritability ($\hat{h}^2_{SNP} > 0.1$) and $> 400K$ records for additive-by-additive genetic variance. We used a bin-based approach where we fitted a weighted linear regression in R[13] (where weights were equal to N_k pairs per bin) of the phenotypic covariance per bin on the linear (π_k) and quadratic (π_k^2) functions of the average genomic relationship. Coefficients for the quadratic term indicate the additive-by-additive variance for all traits was not significantly different from zero (p > 0.05/14; Supplementary Table 8), though the additive-by-additive term was nominally significant for both height and body fat percentage.

Supplementary Table 8. Additive-by-additive variance estimates for 14 traits using a weighted linear regression in the UK Biobank. Shown is the coefficient, standard error (s.e.) and two-sided *p*-value for the quadratic (π^2) term (where *t*-value $\sim t_{df=4}$).

**Trait**	**coefficient**	**s.e.**	***p-value***
height	-0.200	0.060	0.029
body mass index	0.234	0.118	0.119
body fat percentage	0.503	0.141	0.023
metabolic rate	0.215	0.165	0.264
waist to hip	0.128	0.165	0.481
hand grip strength	0.112	0.131	0.439
skin colour	0.045	0.093	0.652
skin tanning	0.145	0.087	0.173
educational attainment	-0.012	0.169	0.945
white blood cell count	-0.035	0.206	0.873
red blood cell count	0.312	0.163	0.128
platelet cell count	0.169	0.083	0.113
eosinophil cell count	-0.115	0.150	0.486
sit to stand ratio	0.019	0.143	0.901

The complete models (with standard errors) for height and body fat percentage are:

height = 0.042 (0.003) + 1.037 (0.036) π – 0.200 (0.060) π^2

bodyFat = 0.019 (0.008) + 0.225 (0.084) π – 0.503 (0.141) π^2

and observed data with the modelled relationship is shown in Supplementary Figure 12. Contrary to expectations under additive-by-additive effects (i.e. Supplementary Figure 11), a negative coefficient for π^2 was estimated for height. This seems driven predominately by monozygotic (MZ) twin pairs having lower covariance than expected under a linear model, and is likely to be an artefact of assortative mating. That is, assortative mating inflates the genetic variance in all close relative pairs except MZ twins. Caution is also needed for the interpretation of the (nominally) significant result for body fat percentage. An alternate model, for example, that included common environmental effects for full-sibs ($\pi \sim 0.5$) and monozygotic twins ($\pi \sim 1.0$) could also feasibly explain the non-linear increased covariance for this trait.

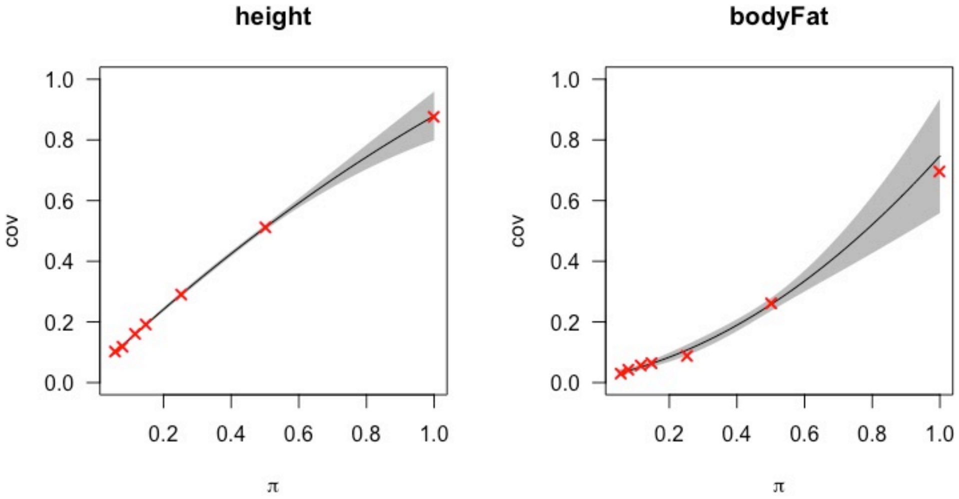

Supplementary Figure 12. Phenotypic covariance in close relatives for height (left) and body fat percentage (right) modelled with a quadratic function for the genomic relationship (π). Red points show the observed data and 95% confidence intervals from the fitted model are indicated in grey.

2.3.1 Power to detect epistasis

We assess the power of our analysis to detect epistatic variation. We provide an analytical solution and verified these calculations via simulation. Details of the analytical solution and simulation are provided in sections 2.3.2 and 2.3.3. The results suggest at least 80% power to detect additive-by-additive genetic variance > $0.45\hat{\sigma}_P^2$ (where $\hat{\sigma}_P^2$ is the phenotypic variance; Supplementary Figure 13).

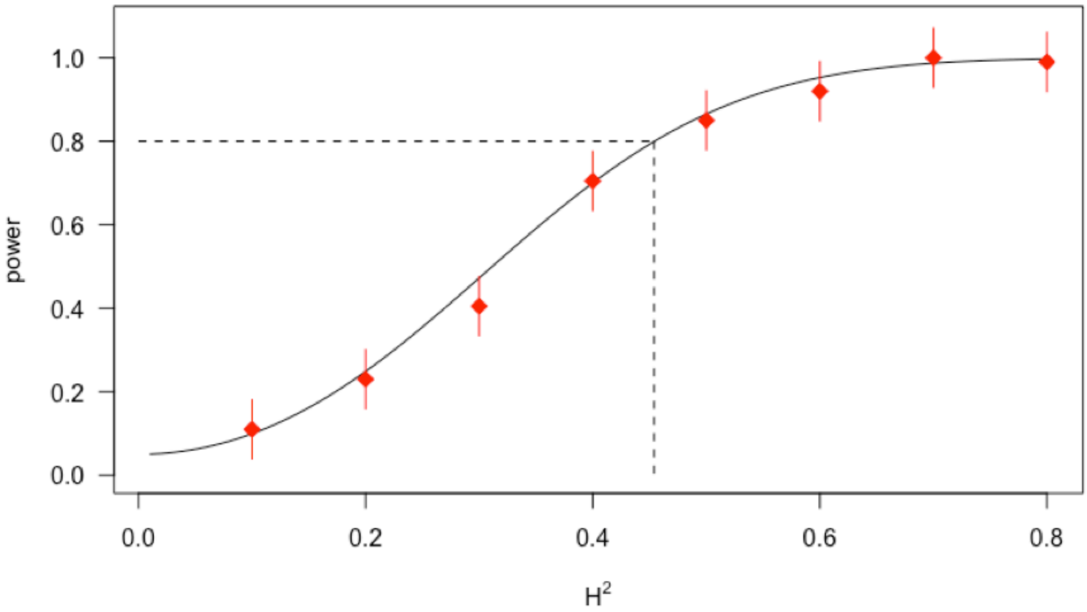

Supplementary Figure 13. Power to detect additive-by-additive genetic variance in the UK Biobank. The solid black line shows the analytical solution, with simulation mean estimates shown in red and bars indicating the standard error of the estimate from 200 replicate simulations.

2.3.2 Power to detect epistasis - analytical solution

The model fitted to the data was:

$\boldsymbol{y} = \boldsymbol{Xb} + \boldsymbol{e}$

where $\boldsymbol{y}$ is the vector of average covariance per bin, $\boldsymbol{X}$ is a the design matrix fitting an overall mean, linear and quadratic terms for the genomic relationship; $\boldsymbol{e}$ is the vector of residual errors distributed $\boldsymbol{N}(\boldsymbol{0}, \boldsymbol{E}\sigma_e^2)$, and $\boldsymbol{E}$ is diag(1/**N**) where **N** is the number of pairs per bin. The sampling variance of $\boldsymbol{b}$ given by $[\boldsymbol{X}'\boldsymbol{E}^{-1}\boldsymbol{X}]^{-1}$, and the non-centrality parameter for the quadratic term is $(b_3/[\boldsymbol{X}'\boldsymbol{E}^{-1}\boldsymbol{X}]^{-1}_{3,3})^2$, with b_3 = H^2. Power was calculated in R using an F-distribution with 1,4 degrees of freedom and a type-1 error rate of 0.05.

2.3.3 Power to detect epistasis - simulation

Our simulations followed the model outlined in sections 2.1.1 and 2.1.2. That is, we simulated phenotypes as the sum of genetic (G) and environmental (E) effects using real genotypes from the UK Biobank, where simulated genetic effects were solely due to 5,000 pairs of unlinked loci with additive-by-additive genetic effects. We calculated the average phenotypic covariance in 7 relationship bins for close relatives ($\pi > 0.05$) in 200 replicate simulations. The broad sense heritably (H^2) of the simulated trait was 0.1, 0.2, 0.3, 0.4, 0.5, 0.6, 0.7 or 0.8. A weighted linear regression in each replicate fitted an overall mean, linear (π) and quadratic (π^2) effects of the average genomic relationship to the average phenotypic covariance per bin. Weights were the number of pairs contributing to each bin. The power to detect epistasis was the proportion of replicates with significant epistatic variance (p < 0.05). Standard errors were approximated as $\frac{1}{\sqrt{n}}$ (where n = 200, i.e. the number of replicates).

SUPPLEMENTARY NOTE 3:
Expectations from full-sib IBD regression analysis under assortative mating

This section summarises known theoretical results from the literature and derives new theoretical results for full-sib IBD regression, validated by simulation. The main finding is that for full-sib IBD regression, and by implication for its generalisation in the RDR method[14], under assortative mating the expectation of the estimate of heritability is neither the population value under random mating nor the population (equilibrium) value under assortative mating.

3.1 Theory - change in genetic & phenotypic variance due to assortative mating

Assortative mating increases the genetic variance in a population, compared to a random mating or base population, and therefore also the phenotypic variance. After several generations of assortative mating equilibrium conditions are reached where the genetic and phenotypic variance remain constant (but increased compared to a random mating or base population). This section of the supplementary note sets out the expected variances after 1 generation of assortative mating, and at equilibrium conditions; and how to convert from base (random mating) population to equilibrium variances (Supplementary Table 9). Theory is reproduced from Table 10.6 (pg. 176) of Falconer and Mackay[15] and Lynch and Walsh[12] (pg. 153-158). Note that y_1 and y_2 are the phenotypes of full-sibs 1 and 2 respectively.

3.2 Theory - expected values from full-sib IBD regression

The phenotype (**y**) can be modelled as:

$$\mathbf{y} = \mathbf{1}\mu + \mathbf{a} + \mathbf{c} + \mathbf{e} \qquad \text{[i]}$$

where **1** is a vector of 1's, μ is the overall mean, **a** is vector additive genetic effects distributed N(0,$\mathbf{A}\sigma^2_a$), **c** is the common environment for pairs of individuals from the same family distributed N(0,$\mathbf{E}\sigma^2_c$), **e** is the vector of residuals from the model distributed N(0,$\mathbf{I}\sigma^2_e$), **A** is a genetic relationship matrix with 1's on the diagonal and proportion of alleles identity-by-descent (IBD) on the off-diagonals for full-sibs, **E** is a family environment matrix with 1's on the diagonal and 1's on the off-diagonal for full-sib pairs, **I** is an identity matrix, σ^2_a is the additive genetic variance, σ^2_c is the variance attributed to family effects and σ^2_e is the residual variance. Thus var(**y**) = $\mathbf{A}\sigma^2_a + \mathbf{E}\sigma^2_c + \mathbf{I}\sigma^2_e$ in the general case or, specifically for full-sibs,

$$\text{cov}(y_1,y_2) = \sigma^2_c + \bar{\pi}\sigma^2_a \qquad \text{[ii]}$$

where cov(y_1,y_2) is the phenotypic covariance for full-sib pairs and $\bar{\pi}$ is the mean IBD sharing for full-sibs (i.e. $\bar{\pi}$ = 0.5). The variance components can be estimated using restricted maximum likelihood (REML), e.g. in GCTA[16], with the heritability obtained from the full-sib regression (h^2_{FS}) defined as $\sigma^2_a / (\sigma^2_a + \sigma^2_c + \sigma^2_e)$. The proportion of phenotypic variance explained by family environment (c^2_{FS}) is $\sigma^2_c / (\sigma^2_a + \sigma^2_c + \sigma^2_e)$.

Next, consider an equivalent model based on the cross-product of full-sib pair phenotypes and simple linear regression:

$$z_i = \mu^* + b\pi_i + \varepsilon_i \qquad \text{[iii]}$$

where $z_i = y_1y_2$ with y_1 and y_2 being the phenotypes of sib 1 and 2 from full-sib pair *i*, and μ^* is the regression intercept, *b* is the regression slope, π_i is the genetic relationship (proportion of alleles IBD) for full-sib pair *i* and ε_i is the model residual.

Supplementary Table 9. Expected values under random and assortative mating.

	Parameter	Expected value
Base (random mating) population parameters		
Genetic variance	$\sigma^2_{a(RM)}$	
Environmental variance	σ^2_{e}	
Phenotypic variance	$\sigma^2_{P(RM)}$	$\sigma^2_{a(RM)} + \sigma^2_{e}$
Heritability	h^2_{RM}	$\sigma^2_{a(RM)} / \sigma^2_{P(RM)}$
After 1 generation of assortative mating		
Phenotypic correlation between mates	r	
Genetic variance	σ^2_{a1}	$\sigma^2_{a(RM)}(1 + rh^2_{RM}/2)$
Phenotypic variance	σ^2_{P1}	$\sigma^2_{P(RM)}[1 + r(h^2_{RM})^2/2]$
Heritability	h^2_{1}	$\sigma^2_{a1} / \sigma^2_{P1}$
Full-sib covariance	$\mathrm{cov}(y_1,y_2)$	$½\sigma^2_{a(RM)}(1 + rh^2_{RM})$
Full-sib correlation	$\mathrm{r}(y_1,y_2)$	$\mathrm{cov}(y_1,y_2) / \sigma^2_{P1}$
At equilibrium conditions		
Genetic variance	$\sigma^2_{a(EQ)}$	
Phenotypic variance	$\sigma^2_{P(EQ)}$	$\sigma^2_{a(EQ)} + \sigma^2_{e}$
Equilibrium heritability	h^2_{EQ}	$\sigma^2_{a(EQ)} / \sigma^2_{P(eq)}$
Full-sib covariance	$\mathrm{cov}(y_1,y_2)$	$½\sigma^2_{a(EQ)} (1 + rh_{EQ}^2)$
Full-sib correlation	$r(y_1,y_2)$	$\mathrm{cov}(y_1,y_2) / \sigma^2_{P(EQ)}$
Converting between base & equilibrium		
Genetic variance	$\sigma^2_{a(RM)}$	$\sigma^2_{a(EQ)} (1 – rh_{EQ}^2)$
Phenotypic variance	$\sigma^2_{P(RM)}$	$\sigma^2_{P(EQ)} [1 - r(h_{EQ}^2)^2]$
Heritability	h^2_{RM}	$h^2_{EQ}(1 - rh^2_{EQ})/$ $[(1 - \mathrm{r}(h_{EQ}^2)^2]$

We note and correct a small error in equation (9) from Table 10.6 of Falconer and Mackay (p. 176) by calculating h^2_{RM} as $\sigma^2_{a(RM)} / \sigma^2_{P(RM)}$ and where $\sigma^2_{a(RM)}$ and $\sigma^2_{P(RM)}$ are as defined above, or eq. (5) / eq. (7) in aforementioned reference.

The regression co-efficient b is equivalent to σ^2_a because[17]:

$$\begin{aligned} b &= \mathrm{cov}(\pi, \mathrm{y_1 y_2}) \;/\; \mathrm{var}(\pi) \\ &= \mathrm{cov}(\pi, \mathrm{a_1 a_2}) \;/\; \mathrm{var}(\pi) \\ &= [\mathrm{E}(\pi \mathrm{a_1 a_2}) - \mathrm{E}(\pi)\mathrm{E}(\mathrm{a_1 a_2})] \;/\; \mathrm{var}(\pi) \\ &= [\mathrm{E}(\pi \mathrm{E}(\mathrm{a_1 a_2}|\pi)) - \mathrm{E}(\pi)\mathrm{E}(\mathrm{E}(\mathrm{a_1 a_2}|\pi))] \;/\; \mathrm{var}(\pi) \\ &= [\mathrm{E}(\pi^2)\sigma_a^2 - \mathrm{E}(\pi)\mathrm{E}(\mathrm{E}(\pi))\sigma_a^2] \;/\; \mathrm{var}(\pi) \\ &= \sigma_a^2\, [\mathrm{E}(\pi^2) - \mathrm{E}(\pi)^2)] \;/\; \mathrm{var}(\pi) \\ &= \sigma_a^2 \end{aligned}$$

Then $\mu^* = \bar{z} - \mathrm{b}\bar{\pi} = \mathrm{cov}(y_1,y_2) - \mathrm{b}\bar{\pi}$ [iv]

By rearranging [iv] and comparing to [ii] we can see that $\mathrm{E}(\mu^*) = \sigma_c^2$ and $\mathrm{E(b)} = \sigma_a^2$.

We simulate assortative mating below to verify that the expected value of $\hat{\sigma}_a^2$, the REML estimator of σ_a^2, is the base or random mating genetic variance and thus, in the presence of assortative mating, that $\hat{\sigma}_c^2$ is inflated by the increase in genetic variance created by assortment (i.e. the variance created by gametic phase disequilibrium). We note that h^2_{FS} is then $\sigma^2_{a(RM)}$ / ($\sigma^2_{a(EQ)} + \sigma^2_c + \sigma^2_e$); where the numerator is the random mating genetic variance and the denominator is the phenotypic variance in the current population. Thus, h^2_{FS} estimates neither the random mating (h^2_{RM}) nor equilibrium (h^2_{EQ}) heritability.

3.3 Simulation parameters

A total of 500 replicate simulations were conducted for one generation of either random or assortative mating. Parameters for the simulation are given in Supplementary Table 10. For each full-sib pair the proportion of the genome IBD was calculated from the observed segregation of alleles between siblings (i.e. without error). The mean IBD co-efficient for all pairs was 0.5 with standard deviation 0.038. Phenotypes were generated under the model [i], with genetic effects for individual j calculated as the sum of all allelic effects carried by each individual, i.e. $a_j = \sum \beta$.

Supplementary Table 10. Simulation parameters for 1 generation of mating

	Symbol	Value
Genetic variance in base (parental) population	$\sigma^2_{a(RM)}$	0.7
Environmental variance	σ^2_e	0.3
Phenotypic variance in parental population ($\sigma^2_{a(RM)} + \sigma^2_e$)	$\sigma^2_{P(RM)}$	1.0
Heritability in base or random mating population ($\sigma^2_{a(RM)}/\sigma^2_{P(RM)}$)	h^2_{RM}	0.7
Number of independent loci, from Visscher et al.[18]	M	85
Effect of independent loci	β	N(0,h^2_{RM}/M)
Number of individuals	n	40,000
Correlation between mates	r	0 or 0.6

3.4 Analysis of simulated data

Simulated data was analysed using either the individual model [i] and REML estimation in GCTA v1.9[16] or the full-sib covariance model [iii] and a linear regression in R v3.5.0[13].

3.5 Simulation results - individual model

Supplementary Table 11 shows the mean (and standard error of the mean, s.e.m.) for 500 replicate simulations of the individual model with either random or assortative mating and analysed with REML in GCTA. The expected value for the full-sib covariance from the simulations is not significantly different from the expected value defined in Supplementary Table 9 [$\text{cov}(y_1,y_2) = \frac{1}{2}\sigma^2_{a(RM)}(1 + rh_{RM}^2) = 0.350$ or 0.497], either under random or assortative mating schemes. Assortative mating increases the phenotypic covariance between full-sib pairs.

The estimated variance attributed to additive genetic effects ($\hat{\sigma}_a^2$) under random or assortative mating was not significantly different from the simulated genetic variance in the base population ($\sigma^2_{a(RM)}$= 0.7). The variance attributed to family effects ($\hat{\sigma}_c^2$) is

~ 0 under random mating, and approximately equal to the increase in genetic variance due to assortment when r = 0.6 (i.e. $\sigma_c^2 = \sigma^2_{a1} - \sigma^2_{a(RM)}$ = 0.85 - 0.7 = 0.15). We confirm that $\hat{h}^2_{FS}$ under this model is the random mating genetic variance scaled by the phenotypic variance in the current population ($h^2_{FS} = \sigma^2_{a(RM)} / \sigma^2_{P1}$ = 0.61).

Supplementary Table 11. Mean (and standard error of the mean, s.e.m.) for variance components from the individual model estimated using REML from 500 replicate simulations.

r	cov(y_1,y_2)	$\widehat{\sigma_a^2}$	s.e.m.	$\widehat{\sigma_c^2}$	s.e.m.	$\widehat{\sigma_P^2}$	s.e.m.	$\widehat{\sigma_a^2}/\widehat{\sigma_P^2}$	s.e.m.	$\widehat{\sigma_c^2}/\widehat{\sigma_P^2}$	s.e.m.
0	0.349	0.708	0.007	-0.005	0.003	0.999	0.000	0.708	0.007	-0.005	0.003
0.6	0.497	0.692	0.016	0.149	0.004	1.147	0.000	0.607	0.006	0.130	0.003

3.6 Simulation results - full-sib covariance model

Supplementary Table 12 shows the results from the full-sib covariance regression model. The intercept and slope of the full-sib regression under random mating is in accordance with expected values [i.e. E(μ^*) = 0, E(b) = $\sigma^2_{a(RM)}$]. Similarly, under assortative mating the values obtained under simulation are not significantly different from their expectations, i.e. E(μ^*) = σ^2_c = 0.15 and E(b) = $\sigma^2_{a(RM)}$ = 0.7. Note that scaling of the phenotypes to N(0,1) would have the effect of scaling both the intercept and slope of the regression analysis such that E(μ^*) = $\sigma^2_c / \sigma^2_{P1} = c^2_{FS}$ and E(b) = $\sigma^2_{a(RM)} / \sigma^2_{P1}$ = h^2_{FS}.

Supplementary Table 12. Mean (and standard error of the mean, s.e.m.) intercept ($\widehat{\mu^*}$) and slope ($\hat{b}$) for 500 replicate simulations from the full-sib covariance regression model with the mean full-sib covariance (i.e. $\bar{z}$).

r	$\bar{z}$	$\widehat{\mu^*}$	s.e.m.	$\hat{b}$	s.e.m.
0	0.350	0.001	0.004	0.699	0.009
0.6	0.497	0.144	0.005	0.706	0.010

SUPPLEMENTARY NOTE 4:
Expectations of genetic parameters from different experimental designs

This supplementary note describes the expectations of heritability estimates from different experimental designs under assortative mating. We convert each estimate from this study and classic twin estimates from the literature into two comparable parameters, (i) the equilibrium heritability (h^2_{EQ}), and (ii) the random mating genetic variance divided by the equilibrium phenotypic variance [i.e. $h^2_{EQ}(1-rh^2_{EQ})$, under some assumptions and where *r* is the phenotypic correlation between mates]. The experimental designs examined are a classic twin design, full-sib IBD regression analysis, and estimates from complex pedigrees (or close relatives).

4.1 A note on converting between random and assortative mating variance parameters

Under the assumptions of (i) no common environmental variance, (ii) no non-additive genetic variation and (iii) a population in assortative mating equilibrium, we know the relationship between the random mating (RM) and assortative mating (AM) variance parameters. From Supplementary Table 9,

$$h^2_{RM} = h^2_{EQ}(1-rh^2_{EQ})/\,(1-rh^4_{EQ})$$

This can also be written as equilibrium parameters in terms of RM parameters[19],

$$\begin{aligned} h^2_{EQ} &= 2h^2_{RM}/[1+\sqrt{1-4rh^2_{RM}(1-h^2_{RM})}] \\ &= 2h^2_{RM}/[1+t], \end{aligned}$$

Note that when under random mating $r=0$, $t=1$ and $h^2_{EQ}=h^2_{RM}$.

We can now write the expected value of the estimates from twin studies, from full-sib IBD regression and from complex pedigree analysis in terms of either RM or AM parameters.

4.2 Assumed value for *r*, the correlation between mates

In subsequent sections of this supplementary note, we adjust estimates from twin studies and full-sib IBD regression for assortative mating. For adjustments, we need to assume a value for *r*, the correlation between mates. We chose to use estimates independent of our estimate of $\hat{r}$ from close relatives, and therefore obtained phenotypic correlations of spousal pairs from the literature. The correlation between mates for height was assumed to be 0.23, from a published meta-analysis of 154 global populations[20]. For EA we use the inverse weighted variance method[21] to meta-analyse reported spousal correlations from Australia (0.426, *N* = 4830)[22], the US (0.480, *N* = 134)[23] and the UK (0.35 s.e. 0.03). We approximated standard errors as $\sqrt{(1-r^2)/N}$ (where *r* is the correlation between *N* spousal pairs) when they were not provided to obtain an estimate of the spousal correlation for EA as 0.416 (0.012).

4.3.1 Expectations from twin studies

Let r(MZ) and r(DZ) be the monozygotic and dizygotic twin correlations, $\sigma^2_{a(EQ)}$ the additive genetic variance at equilibrium, σ^2_c the common environmental variance, σ^2_d the non-additive genetic variance, $\sigma^2_{P(EQ)}$ the phenotypic variance at equilibrium, and h^2_{EQ}, d^2_{EQ} and c^2_{EQ} be the proportion of equilibrium phenotypic variance explained by additive genetic, non-additive genetic and common environmental variance. Then following Falconer and Mackay[15] (Table 10.5, p. 172) and Lynch and Walsh[12] (Table 7.4, p. 158), the expectations of the twin correlations are:

r(MZ) $= [\sigma^2_{a(EQ)} + \sigma^2_d + \sigma^2_c] / \sigma^2_{P(EQ)}$

r(DZ) $= [0.5\,\sigma^2_{a(EQ)}(1 + rh^2_{EQ}) + 0.25\sigma^2_d + \sigma^2_c] / \sigma^2_{P(EQ)}$

Using Falconer's estimate of the heritability as 2[r(MZ) - r(DZ)] then:

$E(h^2_{Twin}) = 2\,[(\sigma^2_{a(EQ)} + \sigma^2_d + \sigma^2_c - 0.5\,\sigma^2_a(1 + rh^2_{EQ}) - 0.25\,\sigma^2_d - \sigma^2_c) / \sigma^2_{P(EQ)}]$

$= [\sigma^2_{a(EQ)}(1 - rh^2_{EQ}) + 1.5\,\sigma^2_d] / \sigma^2_P$

$= h^2_{EQ}(1 - rh^2_{EQ}) + 1.5d^2_{EQ}$

And Falconer's estimate of the common environmental effect [2r(DZ)-r(MZ)] is:

$E(c^2_{Twin}) = 2\,[0.5\,\sigma^2_{a(EQ)}(1 + rh^2_{EQ}) + 0.25\sigma^2_d + \sigma^2_c] / \sigma^2_{P(EQ)}] - [\sigma^2_{a(EQ)} + \sigma^2_d + \sigma^2_c] / \sigma^2_{P(EQ)}$

$= [\sigma^2_{a(EQ)}(rh^2_{EQ}) - 0.5\sigma^2_d + \sigma^2_c] / \sigma^2_{P(EQ)}$

$= rh^4_{EQ} + 0.5d^2 + c^2$

Assuming there is a negligible contribution from σ^2_d, then under assortative mating 2[r(MZ) - r(DZ)] underestimates h^2_{EQ} and [2r(DZ)-r(MZ)] overestimates the common environmental effect. And also, following section 4.1, the expectation of 2[r(MZ) - r(DZ)] in terms of the random mating heritability is:

$E(h^2_{Twin}) = 2h^2_{RM}(1 + t - 2rh^2_{RM})/(1+t)^2$

4.3.2 Adjusting twin study heritability estimates for AM

A classical twin estimate of heritability is equivalent to $h^2_{EQ}(1 - rh^2_{EQ})$ under the stated assumptions. We can adjust this estimate for assortative mating[22] to estimate the equilibrium heritability. First, let x = 2[r(MZ) - r(DZ)] and y = 2r(DZ) - r(MZ) and then equate x and y with their expectations. Thus, the twin estimate of the equilibrium heritability ($h^2_{Twin\_Adj}$) is $[1 - \sqrt{1 - 4rx}]/2r$ (when r > 0) and the adjusted value for the common environmental variance ($c^2_{Twin\_Adj}$) is $y - rh^4_{Twin\_Adj}$. In Supplementary Table 13 we apply these corrections to recent meta-analysis of twin correlations for height[24] and EA[25], assuming the correlations between mates (r) as described in section 4.2.

Supplementary Table 13. Heritability of height and educational attainment from twin studies with adjustments for assortative mating.

		height (s.e.)	EA (s.e.)
correlation b/w mates*	r	0.23	0.42
MZ twin correlation	r(MZ)	0.908 (0.005)[24]	0.747 (0.015)[25]
DZ twin correlation	r(DZ)	0.543 (0.008)[24]	0.551 (0.018)[25]
Falconer's $\hat{h}^2_{Twin}$	2[r(MZ) - r(DZ)]	0.730 (0.019)	0.392 (0.047)
Falconer's $\hat{c}^2_{Twin}$	2r(DZ) - r(MZ)	0.178 (0.017)	0.355 (0.039)
$\hat{h}^2_{Twin\_Adj}$	$[1 - \sqrt{1-4r\hat{h}^2_{Twin}}]/2r$	0.928 (0.033)	0.493 (0.079)
$\hat{c}^2_{Twin\_Adj}$	$\hat{c}^2_{Twin} - r\hat{h}^4_{Twin\_Adj}$	-0.020 (0.022)	0.254 (0.051)

*see section 4.2, assumed to be known without error.

Standard errors for the above estimates are approximated using the delta-method, based on Taylor series expansions.

First letting x = 2[r(MZ) - r(DZ)] and y = 2r(DZ)-r(MZ), we can show that:

$$var\left(\hat{h}^2_{Twin\_Adj}\right) \approx \frac{var(\hat{x})}{1-4r\hat{x}}$$

$$var\left(\hat{c}^2_{Twin\_Adj}\right) \approx var(\hat{y}) + 4r^2\hat{h}^4_{Twin\_Adj}var(\hat{h}^2_{Twin\_Adj})$$

First note that $E\left(h^2_{Twin\_Adj}\right) = \left[1-\sqrt{1-4rx}\right]/2r$. Then $f(x) = \left[1-\sqrt{1-4rx}\right]/2r$ and $f'(x) = 1/\sqrt{1-4rx}$. Using the delta method, $var[f(x)] \approx var(\hat{x}).[f'(\hat{x})]^2$. Hence $var\left[\hat{h}^2_{Twin\_Adj}\right] \approx var(\hat{x})/(1-4r\hat{x})$.

Next note that $E(c^2_{Twin\_Adj}) = y - rh^4_{Twin\_Adj}$. Then $var\left(\hat{c}^2_{Twin\_Adj}\right) = var(\hat{y}) + r^2var\left(\hat{h}^4_{Twin\_Adj}\right)$, assuming $cov(y, h^4_{Twin\_Adj})$ = 0. Then $var\left(\hat{c}^2_{Twin\_Adj}\right) \approx var(\hat{y}) + r^2var\left(\hat{h}^2_{Twin\_Adj}\right).\left[f'\left(h^2_{Twin\_Adj}\right)\right]^2$, using the delta method. Finally, $var\left(\hat{c}^2_{Twin\_Adj}\right) \approx var(\hat{y}) + 4r^2\hat{h}^4_{Twin\_Adj}var\left(\hat{h}^2_{Twin\_Adj}\right)$.

4.4.1 Expectations from full-sib IBD regression

We showed in Supplementary Note 3 that the expectation for the heritability estimated using full-sib regression is the random mating genetic variance divided by the equilibrium phenotypic variance. Thus,

E[h^2_{FS}] = $E[\sigma^2_{a(RM)}/\sigma^2_{P(EQ)}]$
= $\sigma^2_{a(EQ)}(1 - rh^2_{EQ})/\sigma^2_{P(EQ)}$
= $h^2_{EQ}(1 - rh^2_{EQ})$

Assuming that phenotypes in the current population are standardised (i.e. $\sigma^2_{P(EQ)}$ = 1). Hence under the stated assumptions, the expected values for the full-sib regression and twin analyses are the same.

4.4.2 Adjusting heritability estimates from full-sib IBD regression for AM

The full-sib regression estimate of heritability is h^2_{EQ} (1 - rh^2_{EQ}) and we can adjust this estimate for assortative mating to estimate the equilibrium heritability. First, let x = h^2_{FS} and note that $r[h^2_{EQ}]^2 - h^2_{EQ} + h^2_{FS} = 0$, then $h^2_{EQ} = [1 - \sqrt{1-4rx}]/2r$ (when r > 0). We apply this correction in Supplementary Table 14 to the heritability estimated from the full-sib IBD regression meta-analysis. Standard errors are approximated using the delta-method following the outlined adjustment for the twin design.

Supplementary Table 14. Heritability for height and educational attainment from the meta-analysis of full-sib IBD regression estimates, with an adjustment for assortative mating.

		height (s.e.)	EA (s.e.)
Correlation b/w mates*	r	0.23	0.42
Heritability FS meta-analysis	$\hat{h}^2_{FS}$	0.658 (0.065)	0.284 (0.110)
Equilibrium heritability	$[1 - \sqrt{1-4r\hat{h}^2_{FS}}]/2r$	0.808 (0.104)	0.329 (0.122)

*estimates from section 4.2, assumed to be known without error.

4.5.1 Expectations from complex pedigrees

In the main text, the inflation factors for correlation between relatives with a relationship of $\theta = 1/(d+1)$ is approximately $(1 + rh^2_{EQ})^d$, where d is the number of meiosis apart ($d = 0$ for MZ twins). We model the effects of AM explicitly in the main paper and estimate h^2_{EQ} for traits with evidence of AM (i.e. height and EA). In the general case there is no simple solution as the expectation is averaged over different relative pairs and so depends on the specific experimental design. However, if we assume that the population consists of relative pairs with $\theta = \frac{1}{16}, \frac{1}{8}, \frac{1}{4}, \frac{1}{2}$ and 1, with the proportion of pairs $p_{d(i)}$ then,

$$E[h^2_{complex}] \approx h^2_{EQ} \sum [p_i(1 + rh^2_{EQ})^{d(i)}]$$

4.5.2 Adjusting the equilibrium heritability to be comparable to twin and full-sib IBD estimates

We can also adjust the estimate of the equilibrium heritability so that the estimate is directly comparable to h^2_{FS} and h^2_{Twin}. The expected value for h^2_{FS} and h^2_{Twin} is h^2_{EQ} (1 - $r\,h^2_{EQ}$), as outlined above. In addition, the expected value of the 'common environmental' effect is the additional genetic variance generated by AM (i.e. $h^2_{EQ} - h^2_{FS}$). The estimates of equilibrium heritability ($\hat{h}^2_{EQ}$) and correlation between mates ($\hat{r}$) and their standard errors are estimated in this paper from modelling AM in close relatives (i.e. from Supplementary Table 5). Adjusted values, with standard errors from the block jack-knife design with 100 blocks of individuals, are provided in Supplementary Table 15. Note that $\hat{r}$ inferred from genetic data is similar to the meta-analysis of phenotypic spousal-pair correlations (in section 4.2) for height, but higher

than the phenotypic correlation (in section 4.2) for EA. The discrepancy for EA is discussed further in the main text, but could be caused by strong phenotypic assortment on a trait genetically correlated with EA.

Supplementary Table 15. Conversion of the estimate of equilibrium heritability for height and educational attainment from close relatives into parameters comparable to those obtained under twin and full-sib IBD regression designs.

		height (s.e.)	EA (s.e.)
Equilibrium heritability	$\hat{h}^2_{EQ}$	0.819 (0.035)	0.421 (0.043)
Correlation b/w mates	$\hat{r}$	0.237 (0.036)	0.596 (0.192)
Heritability, FS reg.	$\hat{h}^2_{EQ}(1-\hat{r}\hat{h}^2_{EQ})$	0.660 (0.045)	0.315 (0.055)
Comm. Enviro., FS reg.	$\hat{h}^2_{EQ}-\hat{h}^2_{EQ}\left(1-\hat{r}\hat{h}^2_{EQ}\right)$	0.159 (0.012)	0.105 (0.014)

SUPPLEMENTARY NOTE 5:
Model parameterisation with indirect genetic effects

The purpose of this section is to show how highly parameterised models can become that attempt to draw inference on direct and indirect genetic effects on complex traits and, therefore, that caution needs to be applied when simplified models are used that draw strong conclusions. We begin by noting that there is a strong theoretical body of literature on indirect (associative) effects, as summarised in Lynch and Walsh[12] for maternal effects and in much more detail and with more generality in Walsh and Lynch[26]. The primary literature is from quantitative genetics applied to plant and animal breeding and from evolutionary genetics.

We focus on the simple design of a nuclear family with 2 siblings and assume that we have perfect genetic transmission and phase information. We assume the absence of non-additive genetic variation and non-nuclear (cytoplasmic) inheritance and note that their effects would require additional parameters in the model and would induce confounding with other parameters. We also assume a variance component model, which models composite effects of unobserved or unknown traits in relatives (parents, sib) that influence the phenotype of interest in the focal individual. An alternative approach is a trait-based model, for which the traits in relatives that affect the phenotype of interest in the focal individual need to be known[26].

5.1 Gametic model

Let subscripts T, N, M, P and S denote transmitted, non-transmitted, maternal, paternal and sibling, and x be the (0-1) indicator variable for an allele. The phenotype y of one of the two siblings can be written as a function of transmitted and non-transmitted effects, and their interactions,

$$y = b_{TM}x_{TM} + b_{TP}x_{TP} + b_{NM}x_{NM} + b_{NP}x_{NP} + b_{SM}x_{SM} + b_{SP}x_{SP} + b_{TMTP}x_{TM}x_{TP} + \ldots.. + b_{SMSP}x_{SM}x_{SP} + e$$

There are 6 main allelic effects in this model and 15 pairwise interactions. A number of the interactions have a clear interpretation, for example the effect b_{TMTP} is a parent-of-origin effect. The variance of y is partitioned into the contribution of the 6 main effects, their interactions and the contribution a large number of covariance terms. Again, some of these covariance terms have a clear interpretation, for example $cov(x_{TM}, x_{TP})$, $cov(x_{TM}, x_{NM})$, $cov(x_{TM}, x_{NP})$ and $cov(x_{NM}, x_{NP})$ can all be non-zero when there is gametic phase disequilibrium, e.g. under assortative mating.

5.2 Whole genome variance component model

We model the phenotype of an offspring as a function of direct (d) and indirect effects, with the indirect effects partitioned into a Maternal, Paternal and Sibling effect. When considering the phenotype of the offspring, these indirect effects are environmental sources of variation, even though the unknown traits through which the indirect effects operate can have genetic components (A).

$$y = y_d + M + P + S = A_d + E_d + (A_m + E_m) + (A_p + E_p) + (A_s + E_s)$$

Even without interactions, there are 7 variance components (plus a residual) and up to 21 covariances to estimate.

Young et al.[14] simplify this model by fitting, in their notation,

$y = A_d + A_{par} + E_d$,

with $var(y) = var(A_d) + var(A_{par}) + cov(A_d, A_{par}) + var(E_d)$.

In this model, A_{par} includes the effect of the environment of the focal (offspring) individual that is correlated with the average genotype of the parents.

5.3 Estimation of genetic variance from sibling design ("full-sib IBD regression")
If we have only 2 sibs per family and there is an indirect (social, subscript s) effect of one sibling on the other, then, following the notation and derivations in Walsh and Lynch[26] (chapter 22):

$y_1 = A_{d1} + E_{d1} + A_{s2} + E_{s2}$ and likewise $y_2 = A_{d2} + E_{d2} + A_{s1} + E_{s1}$

(Bijma et al.[27] define a 'total breeding value' as $A_{Ti} = A_{di} + A_{si}$).

Let π be the correlation in additive genetic values between the sibs. This could be the expected value (½) or the realised IBD value. It follows that

$var(y) = var(A_d) + var(E_d) + var(A_s) + var(E_s) + 2\pi cov(A_s, A_d)$,

and $cov(y_1, y_2) = \pi[var(A_d) + var(A_s)] + 2cov(A_s, A_d)$

Therefore, the expectation of the estimate of additive genetic variance of the sib-IBD regression is $[var(A_d) + var(A_s)]$ and the intercept is $2cov(A_s, A_d)$.

This ignores the contribution of parental indirect effects and the contribution of a common environmental component.

References

1. Yang, J., Zeng, J., Goddard, M.E., Wray, N.R. & Visscher, P.M. Concepts, estimation and interpretation of SNP-based heritability. *Nature Genetics* **49**, 1304 (2017).
2. Manichaikul, A. *et al.* Robust relationship inference in genome-wide association studies. *Bioinformatics* **26**, 2867-73 (2010).
3. Bycroft, C. *et al.* The UK Biobank resource with deep phenotyping and genomic data. *Nature* **562**, 203-209 (2018).
4. Abecasis, G.R., Cherny, S.S., Cookson, W.O. & Cardon, L.R. Merlin--rapid analysis of dense genetic maps using sparse gene flow trees. *Nature Genetics* **30**, 97-101 (2002).
5. Okbay, A. *et al.* Genome-wide association study identifies 74 loci associated with educational attainment. *Nature* **533**, 539-42 (2016).
6. The Haplotype Reference Consortium *et al.* A reference panel of 64,976 haplotypes for genotype imputation. *Nature Genetics* **48**, 1279 (2016).
7. The UK10K Consortium *et al.* The UK10K project identifies rare variants in health and disease. *Nature* **526**, 82 (2015).
8. Chang, C.C. *et al.* Second-generation PLINK: rising to the challenge of larger and richer datasets. *Gigascience* **4**, 7 (2015).
9. Yang, J. *et al.* Common SNPs explain a large proportion of the heritability for human height. *Nature Genetics* **42**, 565-569 (2010).
10. Wray, N.R. Allele frequency and the r2 measure of LD. *Twin Research and Human Genetics* **8**, 87-94 (2005).
11. Yang, J. *et al.* Genetic variance estimation with imputed variants finds negligible missing heritability for human height and body mass index. *Nature Genetics* **47**, 1114 (2015).
12. Lynch, M. & Walsh, B. *Genetics and Analysis of Quantitative Traits*, (Sinauer Associates Inc., Sunderland, USA, 1998).
13. R Development Core Team. R: A language and environment for statistical computing. (R Foundation for Statistical Computing, Vienna, Austria, 2020).
14. Young, A.I. *et al.* Relatedness disequilibrium regression estimates heritability without environmental bias. *Nature Genetics* **50**, 1304-1310 (2018).
15. Falconer, D.S. & Mackay, T.F.C. *Introduction to Quantitative Genetics*, (Pearson Education Limited, Edinburgh, UK, 1996).
16. Yang, J., Lee, S.H., Goddard, M.E. & Visscher, P.M. GCTA: A tool for genome-wide complex trait analysis. *American Journal of Human Genetics* **88**, 76-82 (2011).
17. Visscher, P.M. *et al.* Statistical Power to Detect Genetic (Co)Variance of Complex Traits Using SNP Data in Unrelated Samples. *PLOS Genetics* **10**, e1004269 (2014).
18. Visscher, P.M. *et al.* Assumption-Free Estimation of Heritability from Genome-Wide Identity-by-Descent Sharing between Full Siblings. *PLOS Genetics* **2**, e41 (2006).
19. Nagylaki, T. Assortative mating for a quantitative character. *Journal of Mathematical Biology* **16**, 57-74 (1982).

20. Stulp, G., Simons, M.J.P., Grasman, S. & Pollet, T.V. Assortative mating for human height: A meta-analysis. *American Journal of Human Biology* **29**, e22917 (2017).
21. Hemani, G. *et al.* Inference of the Genetic Architecture Underlying BMI and Height with the Use of 20,240 Sibling Pairs. *The American Journal of Human Genetics* **93**, 865-875 (2013).
22. Baker, L.A., Treloar, S.A., Reynolds, C.A., Heath, A.C. & Martin, N.G. Genetics of educational attainment in Australian twins: Sex differences and secular changes. *Behavior Genetics* **26**, 89-102 (1996).
23. Price, R.A. & Vandenberg, S.G. Spouse similarity in American and Swedish couples. *Behavior Genetics* **10**, 59-71 (1980).
24. Polderman, T.J.C. *et al.* Meta-analysis of the heritability of human traits based on fifty years of twin studies. *Nature Genetics* **47**, 702 (2015).
25. Branigan, A.R., McCallum, K.J. & Freese, J. Variation in the Heritability of Educational Attainment: An International Meta-Analysis. *Social Forces* **92**, 109-140 (2013).
26. Walsh, B. & Lynch, M. *Evolution and Selection of Quantitative Traits*, (Oxford University Press, 2018).
27. Bijma, P., Muir, W.M. & Van Arendonk, J.A.M. Multilevel selection 1: Quantitative genetics of inheritance and response to selection. *Genetics* **175**, 277-288 (2007).
